# Interventions for submacular haemorrhage: A systematic review and network meta‐analysis of controversies—On behalf of the Spanish Vitreo‐Retinal Society (SERV)

**DOI:** 10.1111/aos.17570

**Published:** 2025-08-07

**Authors:** Salvador Pastor‐Idoate, Pablo Redruello‐Guerrero, Laura de Juan Hernández, Gregorio Benites‐Narcizo, Mario Rivera‐Izquierdo, José García‐Arumí, José Carlos Pastor Jimeno

**Affiliations:** ^1^ Department of Ophthalmology Clinical University Hospital Valladolid Spain; ^2^ Institute of Applied Ophthalmobiology (IOBA) University of Valladolid Valladolid Spain; ^3^ European Reference Network Dedicated to Rare Eye Diseases (ERN‐EYE) Valladolid Spain; ^4^ Networks of Cooperative Research Oriented to Health Results, RICORS‐REI, National Institute of Health Carlos III Madrid Spain; ^5^ Department of Preventive Medicine and Public Health University of Granada Granada Spain; ^6^ Centros de Investigación Biomédica en Red de Epidemiología y Salud Pública (CIBERESP) Madrid Spain; ^7^ Instituto de Investigación Biosanitaria de Granada (ibs.GRANADA) Granada Spain; ^8^ Vall d'Hebron University Hospital Barcelona Spain; ^9^ Ocular Microsurgery Institute (IMO) Barcelona Spain; ^10^ Department of Ophthalmology Autonomous University of Barcelona Barcelona Spain

**Keywords:** age‐related macular degeneration, anti‐vascular endothelial growth factor, pars plana vitrectomy, pneumatic displacement, polypoidal choroidal vasculopathy, submacular haemorrhage, subretinal haemorrhage, tissue plasminogen activator

## Abstract

**Purpose:**

This systematic review aims to evaluate and synthesize the existing literature on the interventions used for submacular haemorrhage (SMH), highlighting the controversies and differences in clinical practice.

**Method:**

A systematic review was conducted following the PRISMA guidelines. A comprehensive search was performed across multiple databases, including MEDLINE, EMBASE and Cochrane Library, to identify studies on SMH treatment. Inclusion criteria encompassed randomized controlled trials, cohort studies and case series that focused on different therapeutic interventions. Data on functional outcomes, efficacy and safety of the interventions were extracted and analysed.

**Results:**

The review included 150 studies, of which 38 were included in the network meta‐analysis. The analysis of best corrected visual acuity (BCVA) Included 26 studies, 20 interventions and 2125 eyes. Heterogeneity was moderate ( *I* ^2^ = 28.9%). Non‐vitrectomy therapies showed better BCVA outcomes and fewer complications (e.g. retinal detachment, vitreous haemorrhage), while vitrectomy‐based treatments achieved better anatomical results. According to *P*‐score ranking, “Observation” had the highest probability of being most effective for BCVA (*P*‐score = 0.8051), followed by anti‐VEGF monotherapy and non‐vitrectomy combinations. However, this result should be interpreted cautiously, as the “Observation” group was based on only two studies (26 eyes) with clinical heterogeneity. No publication bias was detected (Egger's test *p* = 0.582).

**Conclusions:**

There is no consensus on a standard evidence‐based treatment for SMH. Minimally invasive strategies are promising, but factors such as timing, lesion size and anti‐VEGF use remain critical. Further large‐scale randomised trials are needed to define optimal management.

## 1 INTRODUCTION

Submacular haemorrhage (SMH), particularly associated with neovascular age‐related macular degeneration (n‐AMD) haemorrhage, retinal arterial macroaneurysms (RAM), blunt trauma, myopia and idiopathic polypoidal choroidal vasculopathy (PCV), represents a significant clinical challenge due to its potentially devastating effects on vision and the lack of a standardized treatment protocol (Ohji, 2024). The primary goal across all treatment modalities is the rapid displacement of submacular blood to prevent photoreceptor damage (Chhatwal et al., 2023).

Current treatment options for SMH include pharmacological and surgical approaches. Pharmacological therapies involve the use of anti‐vascular endothelial growth factor (anti‐VEGF) agents and recombinant tissue plasminogen activator (rtPA). Pneumatic displacement (PD) is another widely used technique, which consists of injecting expansile intravitreal gases—such as perfluoropropane (C3F8) or sulfur hexafluoride (SF6)—combined with appropriate patient positioning to displace the haemorrhage away from the fovea. Other treatments, such as pars plana vitrectomy (PPV), may be performed either alone or in combination with subretinal or intravitreal administration of rtPA, gas or anti‐VEGF agents. Each method has shown varying degrees of success; however, functional outcomes remain generally unsatisfactory, and the optimal treatment strategy is still under debate (Mun et al., 2022).

The variability in treatment outcomes can be attributed to several factors, including the duration and size of the haemorrhage, as well as the presence of underlying conditions such as choroidal neovascularization (CNV). Early intervention appears to be critical, as studies have demonstrated that delays in treatment can result in irreversible retinal damage and poorer visual prognosis. It appears that, regardless of the treatment method, the effectiveness of intervention decreases as the duration of SMH increases. After 2–3 weeks, the potential benefits of treatment become uncertain (Chhatwal et al., 2023).

The administration of rtPA, either intravitreally or subretinally, combined with gas tamponade, has shown promise in displacing submacular blood and potentially improving visual outcomes (Veritti et al., 2024). However, even these minimally invasive approach techniques may also carry risks, including retinal toxicity (especially over 100 μg of rtPA), a retinal tear or detachment, vitreous haemorrhage (VH) and recurrent submacular haemorrhage. Surgical treatment with PPV and adjuncts has been reported to provide greater visual gain and is more appropriate for pre‐existing VH and massive SMH (Zhao et al., 2023). However, the procedure is more complex and may also bring potential complications. In addition, the visual prognosis, especially in more difficult cases or larger SMHs, remains guarded even with surgical treatment.

Despite advancements in techniques and combinations of therapies, no single approach has been universally accepted or standardised for managing SMH, underscoring the need for further research and consensus in clinical practice. This review aims to systematically evaluate and synthesise the existing literature on SMH interventions, focusing on the controversies and differences in clinical practices to highlight areas needing further research and potential standardisation.

## 2 METHODS

A comprehensive search was conducted across multiple databases, including MEDLINE, EMBASE and Cochrane Library, to identify relevant studies on the treatment of SMH. Combinations of the following search with Medical Subject Headings (MeSH) terms were used to identify potentially relevant articles: ‘submacular hemorrhage (or haemorrhage)’, ‘neovascular age‐related macular degeneration’, ‘polypoidal choroidal vasculopathy’, ‘pneumatic displacement’, ‘pars plana vitrectomy’, ‘recombinant tissue plasminogen activator’, ‘Gas and submacular hemorrhage’, ‘Intraretinal recombinant tissue plasminogen activator and and/or intravitreal anti‐VEGF injections subretinal or submacular haemorrhage’, ‘macular surgery and submacular hemorrhage’ and ‘anti‐VEGF’. The search included articles published from 2004 up to June 2024. The reference lists from retrieved articles were examined for additional citations. The findings of the systematic review were reported in accordance with the PRISMA guidelines (Page et al., 2021). Study protocol was recorded in PROSPERO (CRD42024587330). (Pastor‐Idoate et al., 2024).

### 2.1 Inclusion and exclusion criteria

To be included in the combined analysis of clinical outcomes, the articles needed to satisfy the following inclusion criteria:

- Primary research studies with original comparative data with more than one treatment group published in peer‐reviewed journals.
- Studies with well‐defined clinical characteristics identifying SMH in the context of n‐AMD, RAM, blunt trauma, myopia and idiopathic PCV.
- Studies describing treatment interventions in sufficient detail to be grouped with other studies using the same intervention.

Non‐English language articles, review articles, editorials or case reports with fewer than 5 patients were excluded. Additionally, studies that did not provide specific data on SMH or did not report on visual or anatomical outcomes were omitted from the analysis.

### 2.2 Data extraction

Data from the included studies were extracted independently by two reviewers (SPI and LJH). The searches for titles and abstracts were executed electronically, and records were managed by Microsoft Excel software. Data extracted the following information for each publication:

- Study design and characteristics.
- Sample size and patient demographics.
- Type of disease: n‐AMD, RAM, blunt trauma, myopia and idiopathic PCV.
- Type of SMH: subretinal or sub‐RPE.
- Time to intervention and duration of follow‐up.
- Type and details of the intervention(s): Intravitreal injections of anti‐VEGF agents such as bevacizumab (1.25 mg/0.05 mL), ranibizumab (0.5 mg/0.05 mL) or aflibercept (2 mg); rt‐PA administered subretinally or intravitreally in doses ranging from 10 to 100 μg/0.1 mL; gas tamponade using SF6, C3F8 or C2F6; PPV using 23G or 25G systems; and photodynamic therapy (PDT) with verteporfin followed by 689 nm laser application.
- Additional treatments.
- Outcomes related to best corrected visual acuity (BCVA) and anatomical improvements (central retinal thickness (CRT) and percentage of SHM resolution).
- Complications and adverse events.

### 2.3 Quality assessment

The quality of the included studies was assessed by two independent reviewers using GRADE (Grading of Recommendations, Assessment, Development and Evaluations) (Guyatt et al., 2008). This tool makes it possible to classify the levels of evidence with high, moderate, low and very low certainty. The criteria used for this classification are based on the risk of bias, imprecision, inconsistency, indirect data collection and publication bias. Discrepancies in quality assessments were resolved through discussion and consensus between the reviewers.

### 2.4 Data synthesis

A narrative synthesis of the results was conducted due to the heterogeneity of the included studies. The combined analysis of BCVA outcomes, expressed in LogMAR units, was performed by aggregating studies that used the most common outcome metrics, such as a gain of 2 lines of vision, and weighting them based on the size of each study.

Where possible, the median visual acuity (VA) was used to account for the uncertainty regarding an appropriate numerical allocation for counting fingers vision, light perception (LP), and no light perception vision. When the mean VA had to be used, we assigned 2.1 for counting fingers vision, 2.4 for hand motions, 2.7 for LP and 3.0 for no LP, where each increment represents a doubling of the visual angle. The primary outcomes analysed were the degree of displacement or regression of the SMH from the fovea and changes in BCVA after treatment, while the secondary outcomes included: duration of symptoms and haemorrhage size, anatomical improvements (CRT), adjuvant therapies (anti‐VEGF injections), and the incidence of complications particularly with recurrent SMH, VH and other complications.

### 2.5 Data analysis

Direct and indirect comparisons of the different therapeutic alternatives were analysed by means of a frequentist network meta‐analysis. All outcome variables were collected, and the mean difference between the last measurement taken and the measurement at diagnosis was calculated, where necessary. The effect size of the different comparisons was calculated using a standardized mean difference. The different therapeutic alternatives were compared with no intervention through observation of the patients. Cochrane's *Q* and Higgins' and Thompson's *I* ^2^ statistic were used to assess study heterogeneity and network consistency. Direct and indirect comparisons could be ascertained by creating network plots. In addition, multiple parallelism and average path length were obtained and interpreted as proposed (König et al., 2013). The effect sizes of the different comparisons were calculated. The ranking of the effectiveness of the different treatments was assessed by means of a *P*‐score, similar to the SUCRA score (Rücker & Schwarzer, 2015). It is important to note that the *P*‐score is based solely on the point estimates and their standard errors, and does not incorporate sample size, clinical heterogeneity or study quality. Therefore, *P*‐score‐based rankings may favour interventions that are not statistically inferior to others, even if they are supported by limited or heterogeneous evidence (Riley et al., 2017). The validity of the results was analysed using different approaches. On the one hand, heat plots were created to analyse the overall inconsistency of the network and to find out which studies contributed the most to it. On the other hand, network consistency was assessed by comparing direct and indirect estimates and the *p*‐value derived from this comparison. Finally, comparison‐adjusted publication bias analysis was assessed using Egger's test and the funnel plot. All analyses were performed with the meta, dmetar and netmeta (Harrer et al., n.d.) packages in R Core Team (2021).

## 3 RESULTS

### 3.1 Selection of relevant studies

**FIGURE 1:**
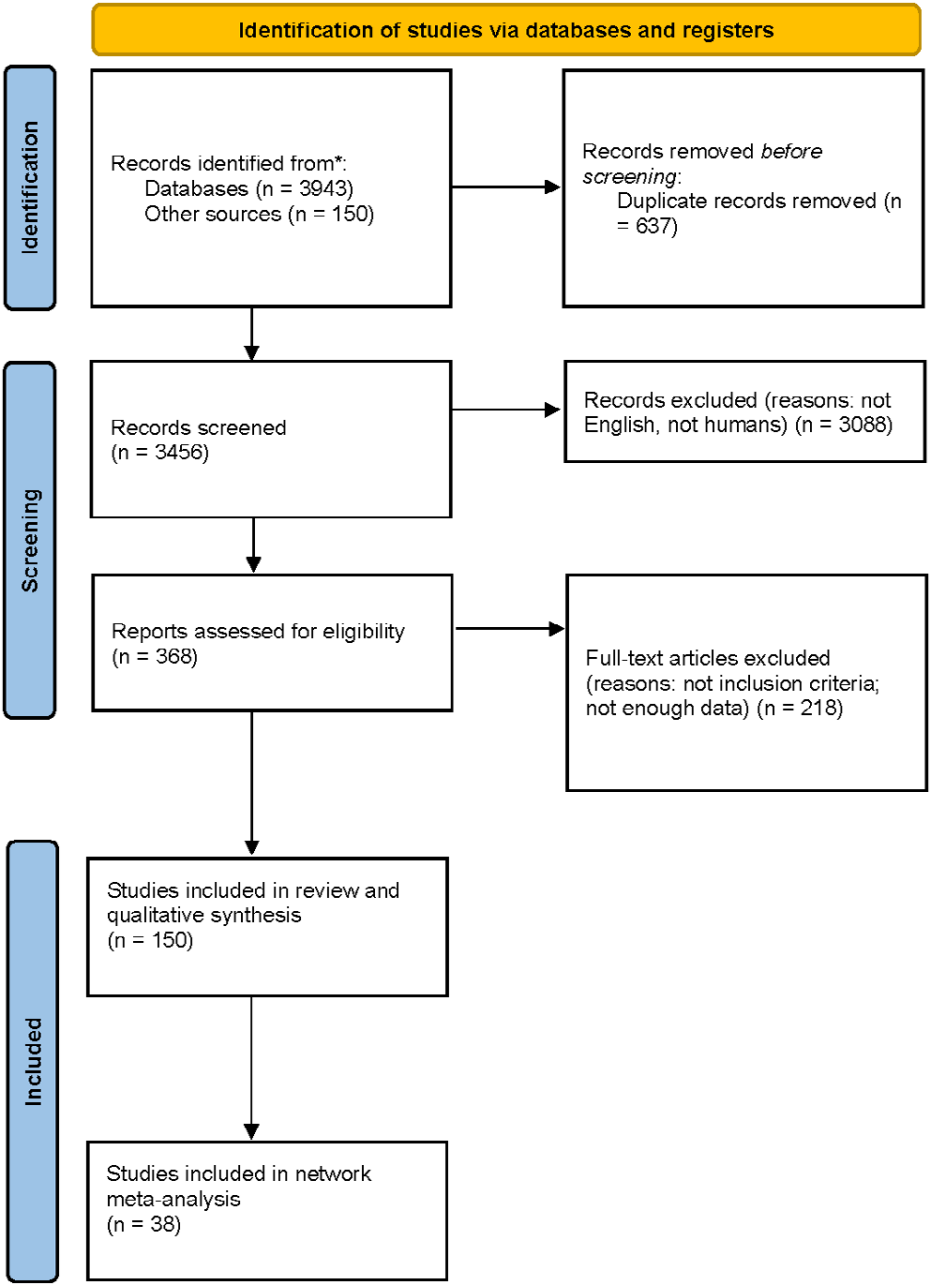
Flow chart diagram.

A total of 150 studies were included in the final analysis, collecting data from 9884 eyes from a total of 6433 patients. Figure 1 summarises the detailed flow diagram of the study identification process.

**FIGURE 2:**
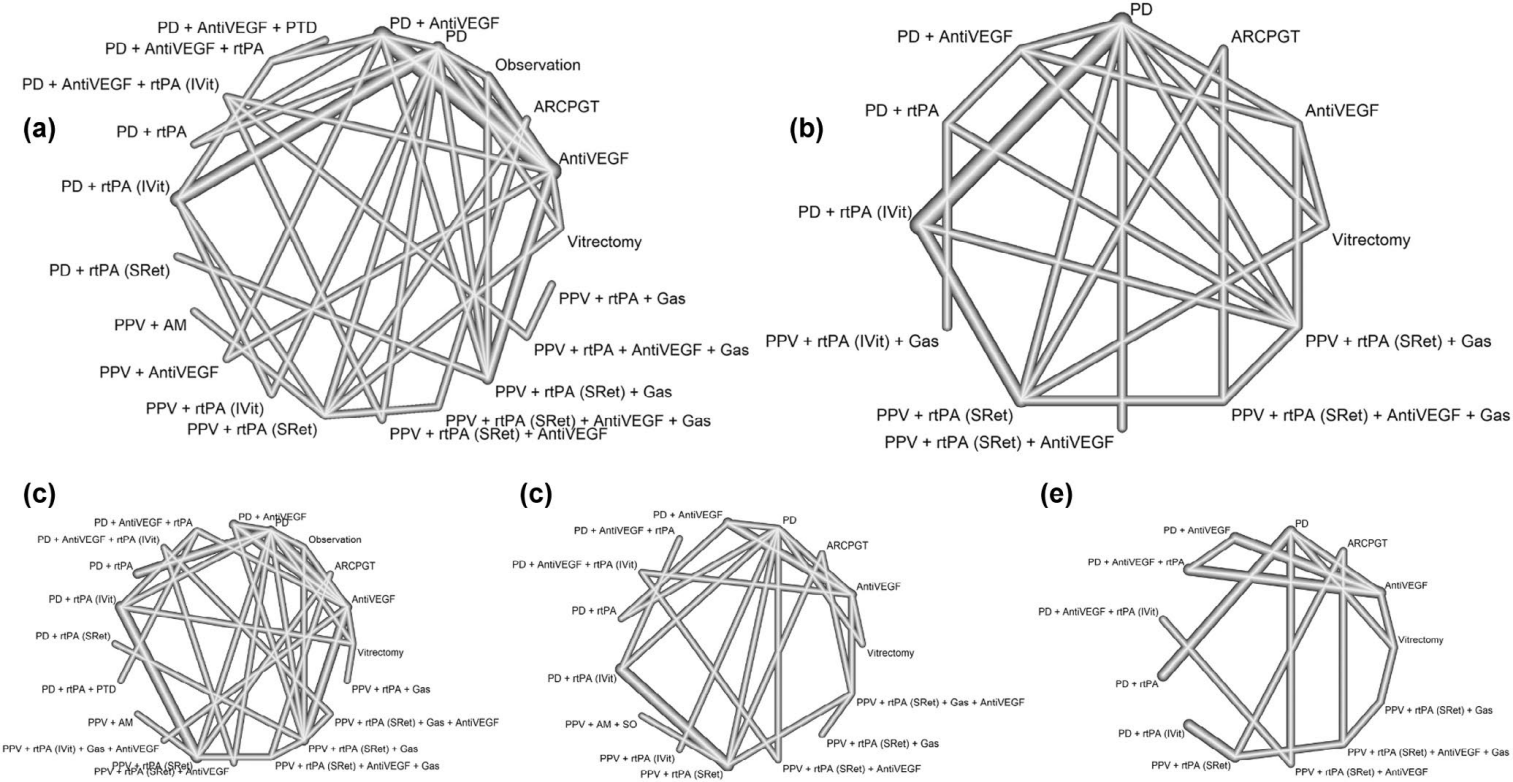
Network diagram comparing medical and surgical treatments for SMH (a, BCVA; b, SMH resolution; c, RD; d, VH; e, recurrent SMH). The thickness of the vectors shows the number of comparisons made between the different therapies. The ‘observation’ group includes only two studies (26 eyes), one of which permitted rescue vitrectomy in 38% of cases and the other included patients with macroaneurysms. This heterogeneous nature limits its classification as a pure observation arm. AM, amniotic membrane; AntiVEGF, anti‐vascular endothelial growth factor; ARCPGT, autologous retinal pigmentary retinal pigment epithelium‐choroid patch graft transplantations; IVit, intravitreal injection; PD, pneumatic displacement; PPV, pars plana vitrectomy; PTD, photodynamic therapy; RD, retinal detachment; rtPA, recombinant tissue plasminogen activator; SRet, subretinal; VH, vitreous haemorrhage.

For the network meta‐analysis, 38 articles (Barayev et al., 2024; Bell et al., 2017; Boral et al., 2023; Cakir et al., 2010; Caporossi et al., 2022; Cho et al., 2015, 2020; Fang et al., 2009; Fassbender et al., 2016; Fujikawa et al., 2013; Gabrielle et al., 2023; Grohmann et al., 2020; Gujral et al., 2019; Guthoff et al., 2011; Hesgaard et al., 2012; Inoue et al., 2022; Jeong et al., 2020; Kang et al., 2018; Kishikova et al., 2021; Kitahashi et al., 2014; Lee et al., 2021; Mayer et al., 2013; Mizutani et al., 2011; Mun et al., 2022; Nourinia et al., 2010; Papavasileiou et al., 2013; Rickmann et al., 2021; Rishi et al., 2012; Sandhu et al., 2010; Shin et al., 2015; Sniatecki et al., 2021; Szeto et al., 2024; Thompson & Sjaarda, 2005; Tiosano et al., 2023; Tranos et al., 2021; Yang et al., 2005; Ye et al., 2023) were selected that reported on any of the variables of interest. A total of 2121 eyes were included, in which 21 different treatments were applied as shown in Figure 2. In terms of BCVA, 26 studies (Boral et al., 2023; Cakir et al., 2010; Caporossi et al., 2022; Cho et al., 2015, 2020; Fassbender et al., 2016; Fujikawa et al., 2013; Gabrielle et al., 2023; Grohmann et al., 2020; Guthoff et al., 2011; Hesgaard et al., 2012; Inoue et al., 2022; Jeong et al., 2020; Kang et al., 2018; Kishikova et al., 2021; Kitahashi et al., 2014; Lee et al., 2021; Mayer et al., 2013; Mun et al., 2022; Nourinia et al., 2010; Papavasileiou et al., 2013; Rickmann et al., 2021; Rishi et al., 2012; Shin et al., 2015; Szeto et al., 2024; Ye et al., 2023) were collected in which 1546 eyes were included in which 21 different treatments were applied allowing for 43 direct comparisons with 22 numbers of designs. For the resolution of SMH, 15 studies (Boral et al., 2023; Cakir et al., 2010; Fang et al., 2009; Gujral et al., 2019; Inoue et al., 2022; Jeong et al., 2020; Kishikova et al., 2021; Kitahashi et al., 2014; Mayer et al., 2013; Rickmann et al., 2021; Rishi et al., 2012; Szeto et al., 2024; Tiosano et al., 2023; Tranos et al., 2021; Ye et al., 2023) were obtained in which 12 treatments were evaluated in 745 eyes with 14 numbers of designs for 25 direct comparisons of interventions. Adverse events included in the network meta‐analysis, based on data availability, were retinal detachment (RD), VH, and SMH recurrence. RD was reported in 24 studies, involving 1532 eyes, with 37 head‐to‐head comparisons across 20 treatments and 22 numbers of designs (Barayev et al., 2024; Bell et al., 2017; Boral et al., 2023; Caporossi et al., 2022; Fujikawa et al., 2013; Gabrielle et al., 2023; Grohmann et al., 2020; Guthoff et al., 2011; Inoue et al., 2022; Jeong et al., 2020; Kishikova et al., 2021; Kitahashi et al., 2014; Lee et al., 2021; Mun et al., 2022; Papavasileiou et al., 2013; Rickmann et al., 2021; Rishi et al., 2012; Sandhu et al., 2010; Shin et al., 2015; Sniatecki et al., 2021; Szeto et al., 2024; Thompson & Sjaarda, 2005; Tranos et al., 2021; Yang et al., 2005). VH was reported in 18 studies, covering 1121 eyes, with 24 comparisons between 15 treatments and 16 numbers of designs (Barzelay et al., 2024; Bell et al., 2017; Boral et al., 2023; Caporossi et al., 2022; Cho et al., 2015; Gabrielle et al., 2023; Guthoff et al., 2011; Inoue et al., 2022; Kitahashi et al., 2014; Lee et al., 2021; Mayer et al., 2013; Mizutani et al., 2011; Rishi et al., 2012; Sandhu et al., 2010; Shin et al., 2015; Sniatecki et al., 2021; Szeto et al., 2024; Yang et al., 2005). SMH recurrence was reported in 13 studies, involving 954 eyes, with 19 comparisons between 13 treatments and 11 numbers of designs (Barzelay et al., 2024; Bell et al., 2017; Boral et al., 2023; Fujikawa et al., 2013; Gabrielle et al., 2023; Inoue et al., 2022; Kang et al., 2018; Lee et al., 2021; Rickmann et al., 2021; Shin et al., 2015; Szeto et al., 2024; Thompson & Sjaarda, 2005; Yang et al., 2005).

**FIGURE 3:**
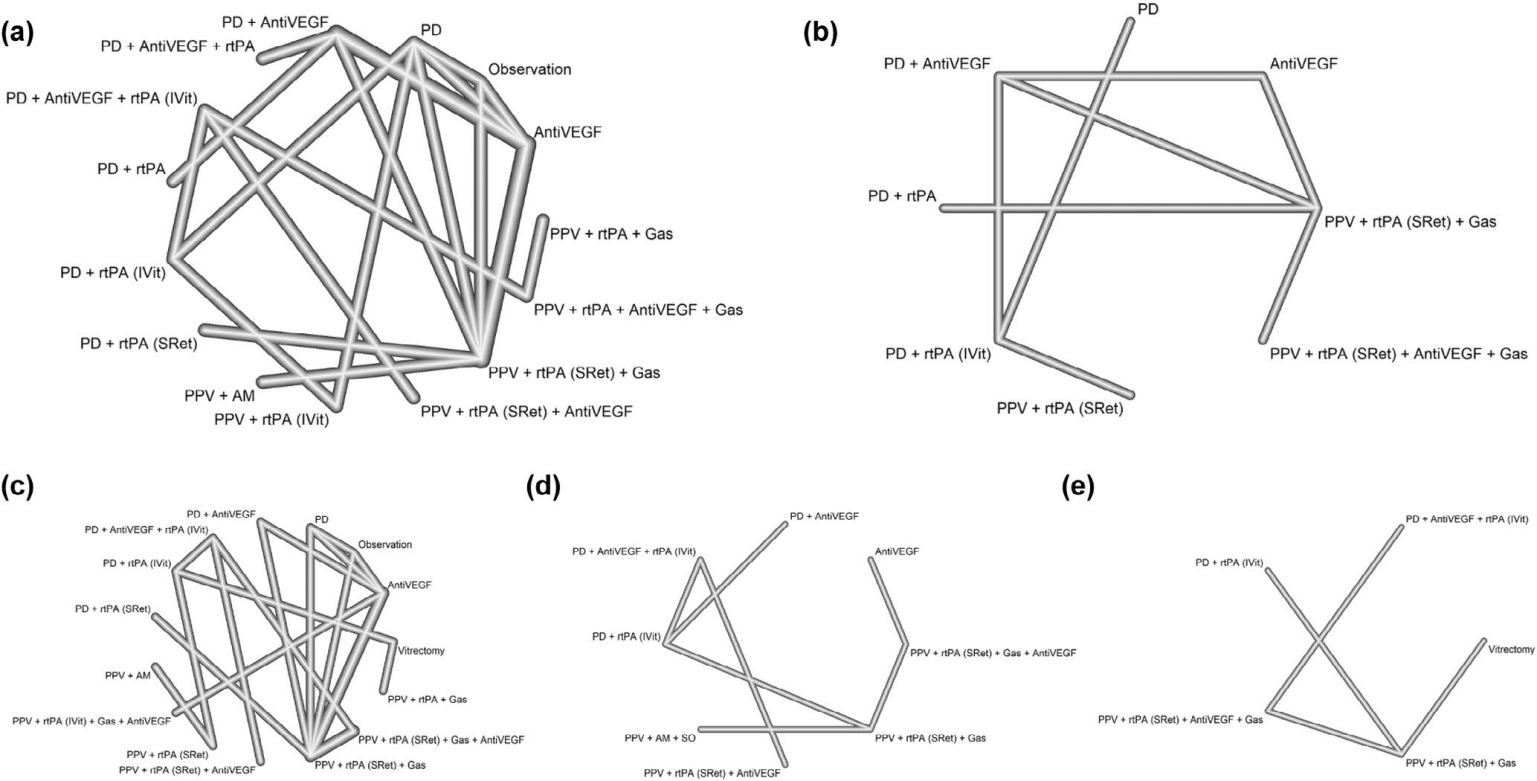
Network diagram comparing medical and surgical treatments for SMH secondary to AMD (a, BCVA; b, SMH resolution; c, RD; d, VH; e, recurrent SMH). The thickness of the vectors shows the number of comparisons made between the different therapies. The ‘observation’ group includes only one study (21 eyes), which permitted rescue vitrectomy in 38% of cases. This heterogeneous nature limits its classification as a pure observation arm. AM, amniotic membrane; AntiVEGF, anti‐vascular endothelial growth factor; ARCPGT, autologous retinal pigmentary retinal pigment epithelium–choroid patch graft transplantations; IVit, intravitreal injection; PD, pneumatic displacement; PPV, pars plana vitrectomy; PTD, photodynamic therapy; RD, retinal detachment; rtPA, recombinant tissue plasminogen activator; SRet, subretinal; VH, vitreous haemorrhage.

To reduce clinical heterogeneity and enhance the interpretability of the findings, a separate network meta‐analysis was conducted focusing exclusively on studies involving patients with n‐AMD. This subgroup was selected because n‐AMD was the only aetiology with a sufficient number of studies and treatment arms to allow for robust and clinically meaningful comparisons. A total of 19 studies (Barayev et al., 2024; Barzelay et al., 2024; Caporossi et al., 2022; Fang et al., 2009; Fassbender et al., 2016; Gabrielle et al., 2023; Grohmann et al., 2020; Guthoff et al., 2011; Hesgaard et al., 2012; Jeong et al., 2020; Kishikova et al., 2021; Mayer et al., 2013; Mun et al., 2022; Nourinia et al., 2010; Rickmann et al., 2021; Sandhu et al., 2010; Sniatecki et al., 2021; Thompson & Sjaarda, 2005; Tranos et al., 2021) were included in this n‐AMD‐specific analysis, comprising 1107 eyes. Figure 3 illustrates the network plot of treatment comparisons included in this subanalysis, showing the direct and indirect connections between the evaluated interventions.

For the n‐AMD‐focused sub‐analysis, BCVA outcomes were reported across 12 studies (Caporossi et al., 2022; Fassbender et al., 2016; Gabrielle et al., 2023; Grohmann et al., 2020; Guthoff et al., 2011; Hesgaard et al., 2012; Jeong et al., 2020; Kishikova et al., 2021; Mayer et al., 2013; Mun et al., 2022; Nourinia et al., 2010; Rickmann et al., 2021), encompassing a total of 712 eyes. These studies evaluated 15 distinct treatment strategies, enabling 21 direct comparisons across 12 different numbers of designs. Regarding SMH resolution, data from 6 studies (Fang et al., 2009; Jeong et al., 2020; Kishikova et al., 2021; Mayer et al., 2013; Rickmann et al., 2021; Tranos et al., 2021) involving 250 eyes were included, assessing 8 treatment options across 6 numbers of designs and yielding 12 direct treatment comparisons.

In terms of safety outcomes, the n‐AMD‐focused network meta‐analysis incorporated data on RD, VH, and SMH recurrence where available. RD was reported in 13 studies (Barayev et al., 2024; Caporossi et al., 2022; Gabrielle et al., 2023; Grohmann et al., 2020; Guthoff et al., 2011; Jeong et al., 2020; Kishikova et al., 2021; Mun et al., 2022; Rickmann et al., 2021; Sandhu et al., 2010; Sniatecki et al., 2021; Thompson & Sjaarda, 2005; Tranos et al., 2021) involving 864 eyes, allowing for 20 direct comparisons among 15 treatments across 12 numbers of designs. VH was reported in 7 studies (Barzelay et al., 2024; Caporossi et al., 2022; Gabrielle et al., 2023; Guthoff et al., 2011; Mayer et al., 2013; Sandhu et al., 2010; Sniatecki et al., 2021) with a combined sample of 374 eyes, providing 7 head‐to‐head comparisons among 8 treatments from 7 numbers of designs. SMH recurrence was evaluated in 4 studies (Barzelay et al., 2024; Gabrielle et al., 2023; Rickmann et al., 2021; Thompson & Sjaarda, 2005), including 325 eyes, across 5 treatments and 4 direct comparisons from 4 different numbers of designs.

### 3.2 Quality assessment and risk of bias

Therapies other than vitrectomy included observation (Cho et al., 2020; Mun et al., 2022); intravitreal injection of antiVEGF (Cho et al., 2013, 2020; Dimopoulos et al., 2015; Hosokawa et al., 2024; Iacono et al., 2014; Jain et al., 2013; Jeong et al., 2020; Kim et al., 2014, 2018, 2020; Kim, Cho, et al., 2015; Kim, Kim, et al., 2015; Kimura et al., 2022; Lee et al., 2021; Maruyama‐Inoue et al., 2023; Matsuo et al., 2021; Mehta et al., 2022; Mun et al., 2022; Sacu et al., 2009; Shienbaum et al., 2013; Shin et al., 2015; Sniatecki et al., 2021; Stifter et al., 2007; Ueda‐Arakawa et al., 2012; Wang et al., 2024); intravitreal injection of rtPA and gas (Araújo et al., 2016; Barayev et al., 2024; Barzelay et al., 2024; Bell et al., 2017; Cakir et al., 2010; Chen et al., 2007; Chew et al., 2022; Fang et al., 2009; Fassbender et al., 2016; Fujikawa et al., 2013; Gabrielle et al., 2023; Gujral et al., 2019; Guthoff et al., 2011; Handwerger et al., 2001; Hattenbach et al., 2001; Hesse et al., 2000; Kabakcı et al., 2021; Karamitsos et al., 2020; Kishikova et al., 2021; Kung et al., 2010; Lim et al., 2020; Maggio et al., 2020; Mayer et al., 2013; McAllister et al., 2010; Mizutani et al., 2011; Mozaffarieh et al., 2006; Muqit & Ghanchi, 2008; Nourinia et al., 2010; Ratanasukon & Kittantong, 2005; Rishi et al., 2012; Schulze & Hesse, 2002; Sobolewska et al., 2014; Tiosano et al., 2023; Tranos et al., 2021; Tsai et al., 2003; Tsymanava & Uhlig, 2012; Wu et al., 2011; Wu & Sheu, 2005; Yang et al., 2005); intravitreal injection of antiVEGF and gas (Abdelkader et al., 2016; Cho et al., 2015; Hesgaard et al., 2012; Jeong et al., 2020; Kabakcı et al., 2021; Kang et al., 2018; Kimura et al., 2022; Kitahashi et al., 2014; Mayer et al., 2013; Shin et al., 2015, 2016; Wakabayashi et al., 2023); PD (Abdelkader et al., 2016; Bae et al., 2016; Cakir et al., 2010; Fang et al., 2009; Fassbender et al., 2016; Fujikawa et al., 2013; Gopalakrishan et al., 2007; Gujral et al., 2019; Inoue et al., 2022; Kimura et al., 2018; Kitahashi et al., 2014; Lee et al., 2020; Lincoff et al., 2008; Matsuo et al., 2021; Mizutani et al., 2011; Mun et al., 2022; Rishi et al., 2012; Ron et al., 2007; Ura et al., 2022); intravitreal injection of rtPA, antiVEGF and gas (Bardak et al., 2018; de Jong et al., 2016; de Silva & Bindra, 2016; Grohmann et al., 2020; Guthoff et al., 2011; Karamitsos et al., 2020; Kitagawa et al., 2016, 2022; Lee et al., 2016, 2020, 2021; Lin et al., 2016; Meyer et al., 2008; Nourinia et al., 2010; Papavasileiou et al., 2013; Sacu et al., 2009); intravitreal injection of rtPA (Tsymanava & Uhlig, 2012); and the combination of PD and PDT (Chan et al., 2005). In the studies included in these therapies, n‐AMD was reported to be the most frequent aetiology. The patients in these studies were mostly men, with a mean age of 72 years and a mean of 11 days had elapsed between symptom onset and intervention. No postoperative complications were reported in 78.79% of patients. In vitrectomy‐based treatments, PPV was combined with intravitreal injection of rtPA and gas (Hillenkamp et al., 2010; Sniatecki et al., 2021); with intravitreal injection of rtPA together with antiVEGF and gas (Arias & Monés, 2010; Grohmann et al., 2020); with subretinal injection of rtPA together with gas and antiVEGF (Ali Said et al., 2021; Avcı et al., 2021; Barayev et al., 2024; Barzelay et al., 2024; Bell et al., 2017; Boiché et al., 2019; Boral et al., 2023; Caporossi et al., 2022; Chang et al., 2014; de Jong et al., 2016; Dewilde et al., 2014; Doi et al., 2020; Erdogan et al., 2020; Fassbender et al., 2016; Fine et al., 2010; Fleissig et al., 2017; Fukuda et al., 2022; Gabrielle et al., 2023; Gok et al., 2017; González‐López et al., 2016; Grohmann et al., 2020; Gujral et al., 2019; Haupert et al., 2001; Helaiwa et al., 2020; Hillenkamp et al., 2010; Hirashima et al., 2015; Iannetta et al., 2021; Iglicki et al., 2023, 2024; Inoue et al., 2015; Jeong et al., 2020; Juncal et al., 2018; Kabakcı et al., 2021; Kadonosono et al., 2015; Kamei & Tano, 2009; Kawakami et al., 2021; Khan et al., 2017; Kimura et al., 2015, 2017, 2022; Kishikova et al., 2021; Kumar et al., 2016; Limon et al., 2023; Lin et al., 2016; Miki et al., 2023; Moisseiev et al., 2014; Mun et al., 2022; Ogata et al., 2022; Olivier et al., 2004; Ozkaya et al., 2018; Patikulsila et al., 2022; Pierre et al., 2021; Plemel et al., 2019; Rickmann et al., 2021; Rishi et al., 2012; Sandhu et al., 2010; Sharma et al., 2018; Shi et al., 2024; Singh et al., 2006; Sniatecki et al., 2021; Sonmez et al., 2012; Szeto et al., 2024; Thompson & Sjaarda, 2005; Tiosano et al., 2023; Tranos et al., 2021; Treumer et al., 2010, 2012, 2017; van Zeeburg et al., 2013; Waizel et al., 2016; Wilkins et al., 2020; Wu et al., 2023; Ye et al., 2023); with removal of SMH and gas (Boral et al., 2023; Caporossi et al., 2022; Han et al., 2013; Thompson & Sjaarda, 2005; Wei et al., 2015); and with subretinal injection of physiological saline solution and gas (Handa et al., 2023). Similarly, the most frequent aetiology was n‐AMD. The patients included were mostly women, with a mean age close to 76 years. The mean time from symptom onset to surgery was 10.79 days. No postoperative complications were reported in 64.01% of patients. All these data can be consulted in Tables 1, 2, 3.

**TABLE 1:** Synthesis of the literature from 2004 to 2024.

Treatment	*N* (eyes)	Mean age (SD)^b^	Median presenting logMAR VA^a^ (*N*); [Snellen equivalent]	Mean presenting logMAR VA^a^ (SD); [Snellen equivalent]	Median final logMAR VA^a^ (SD); [Snellen equivalent]	Mean final logMAR VA^a^ (*N*); [Snellen equivalent]	Any improvement in vision, %, (*N*)	Any worsening in vision, %, (*N*)	Gain of 2 Snellen lines or equivalent, %, (*N*)	Loss of 2 Snellen lines or equivalent, %, (*N*)	Mean best VA after treatment (SD)
Non‐vitrectomy therapies											
Observation	26 (26)	66.47 (10.75)	1.60 (26) [20/800]	1.58 (0.71) [20/800]	1.17 (17) [20/300]	1.18 (0.78) [20/300]	47.05% (8)	29.41% (5)	ND	ND	0.89 (0.46) [20/160]
IVI anti‐VEGF	1.872 (1.503)	73.78 (6.08) ND (39)	1.06 (1.503) [20/200]	1.00 (0.33) [20/200]	0.75 (1.442) [>20/100]	0.70 (0.33) [20/100]	82.03% (1.183)	17.89% (258)	51.31% (740)	15.52% (223)	0.69 (0.31) [20/100]
IVI rTPA + Gas	1.854 (1.359)	71.08 (10.27) ND (9)	1.34 (1.359) [20/400]	1.39 (0.49) [20/500]	0.93 (1.349) [>20/160]	0.89 (0.30) [20/160]	78.05% (1.053)	21.79% (294)	44.32% (598)	13.34% (180)	0.87 (0.30) [<20/160]
IVI anti‐VEGF + Gas	698 (271)	74.68 (6.38) ND (34)	1.28 (271) [20/325]	1.24 (0.25) [20/325]	0.83 (215) [>20/125]	0.75 (0.34) [>20/100]	79.33% (170)	20.46% (44)	60.0% (129)	12.55% (27)	0.72 (0.31) [>20/100]
IVI Gas	1.028 (400)	69.90 (6.87) ND (122)	1.28 (400) [20/400]	1.29 (0.33) [20/400]	0.98 (377) [20/200]	0.93 (0.29) [>20/100]	70.82% (267)	16.44% (62)	46.15% (174)	8.48% (32)	0.91 (0.27) [>20/100]
ITV rTPA + anti‐VEGF + Gas	518 (379)	75.11 (6.59) ND (32)	1.09 (379) [20/200]	1.06 (0.49) [20/200]	0.61(379) [20/80]	0.61 (0.28) [20/80]	82.05% (311)	16.09% (61)	56.99% (216)	14.24% (54)	0.61 (0.28) [20/80]
IVI rTPA	110 (46)	77.70 (7.00)	1.10 (46) [20/250]	ND	1.50 (46) [20/630]	ND	13.04% (6)	30.43% (14)	8.69% (4)	30.43% (14)	ND
IVI Gas + PDT	6 (6)	53.67 (6.31)	1.14 (6) [20/140]	1.14 (0.63) [20/140]	0.45 (6) [20/50]	0.45 (0.34) [20/50]	ND	ND	ND	ND	ND
Total	6.112 (3.990)	72.67 (8.23)	1.22 (3.990) [20/320]	1.19 (0.44) [20/320]	0.84 (3.831) [20/125]	0.80 (0.32) [20/125]	78.25% (2.998)	19.26% (738)	48.57% (1.861)	13.83% (530)	0.78 (0.35) [>20/100]
Vitrectomy therapies											
PPV + IVI rTPA + Gas	63 (63)	81 (9.87)	1.31 (63) [20/400]	1.41 (0.48) [20/500]	1.00 (58) [20/200]	1.03 (0.43) [20/200]	43.10% (25)	27.58% (16)	ND	ND	ND
PPV + IVI rTPA + anti‐VEGF + Gas	87 (49)	80 (9.47)	1.6 (49) [20/800]	1.70 (0.43) [>20/800]	1.2 (49) [20/320]	1.20 (0.18) [>20/320]	67.34% (33)	20.40% (10)	24.48% (12)	16.32% (8)	1.10 (0.29) [20/250]
PPV subretinal rTPA ± Gas ± anti‐VEFG	3.388 (2.196)	76.13 (6.40) ND (37)	1.55 (2.196) [20/800]	1.59 (0.80) [20/800]	0.90 (1.916) [20/160]	1.08 (0.46) [20/200]	69.12% (1.323)	16.29% (315)	48.37% (927)	12.52% (243)	1.05 (0.43) [20/200]
PPV + removal of SMH + Gas	224 (125)	71.24 (10.23)	1.45 (125) [20/600]	1.49 (0.56) [20/630]	1.00 (76) [20/200]	1.04 (0.87) [20/200]	38.15% (29)	25.0% (19)	ND	ND	ND
PPV + Subretinal PSS + Gas	10 (10)	61.20 (16.33)	1.29 (10) [20/400]	1.29 (0.19) [20/400]	1.0 (10) [20/200]	1.0 (0.40) [20/200]	60.0% (6)	40.0% (4)	ND	ND	ND
Total	3.772 (2.443)	75.96 (7.02)	1.6 (2.443) [20/800]	1.58 (0.78) [20/800]	1.00 (2.209) [20/200]	1.08 (0.48) [20/200]	57.96% (1.395)	14.89% (364)	38.43% (939)	10.27% (251)	1.03 (0.31) [20/200]

Abbreviations: IVT, intravitreal; PPV, pars plana vitrectomy; PSS, physiological saline solution; SD, std. deviation; SMH, submacular haemorrhage; TPA, tissue plasminogen activator; VEGF, vascular endothelial growth factor.

^**a**^ Calculated using individual patient data. If individual patient data were not available then the mean was calculated using the study mean, weighted for study size such that larger studies had relatively greater effect on the mean than smaller studies or case reports. Visual acuities were converted to logarithm of the minimum angle of resolution (logMAR).

^b^ Combined mean and standard deviation (SD).

**TABLE 2:** Synthesis of the literature from 2004 to 2024.

Treatment	*N* (eyes)	Mean age (SD)^a^	Sex, %, (*N*)	Type of disease, %, (*N*)	Mean duration between onset of symptoms and treatment (SD, and range)^a^	Mean submacular haemorrhage area (SD, and range)^a^	Mean CRT preop (SD)^a^	Mean CRT posop (SD)^a^	Intraop complications, %, (*N*)	Posop complications, %, (*N*)
Non‐vitrectomy therapies										
Observation	26 (26)	66.47 (10.75)	M, 76.92%, (20) F, 23.08%, (6)	AMD, 80.76%, (21) RAM, 19.23%, (5)	ND (26)	11.14 DA (2.63) (Range: –) ND (5)	550.5 μm (159.26) ND (21)	243.0 μm (60.17) ND (21)	NC, 100%, (26)	NC, 50.0%, (13) REBLEED, 30.76%, (8) ND, 19.23%, (5)
IVI anti‐VEGF	1.872 (1.503)	73.78 (6.08) ND (39)	M, 47.63%, (716) F, 42.98%, (646) ND (141)	AMD, 65.40%, (983) PCV, 18.69%, (281) RAM, 1.13%, (17) Unclassified, 2.12%, (32) ND, 12.64%, (190)	13.47 days (7.98) (4–35) ND (230)	6.76 DA (3.30) (1.5–15) ND (230)	523.0 μm (144.2) ND (331)	287.0 μm (83.02) ND (331)	NC, 87.89%, (1.321) ND (182)	NC, 76.31%, (1.147) VH, 3.26%, (49) REBLEED, 6.52%, (98) RPE TEAR, 1.79%, (27) ND, 12.10%, (182)
IVI rTPA + Gas	1.854 (1.359)	71.08 (10.27) ND (9)	M, 44.15%, (600) F, 52.17%, (709) ND (50)	AMD, 74.90%, (1.018) PCV, 5.96%, (81) RAM, 2.13%, (29) Blunt Trauma, 3.09%, (42) Myopia, 1.17%, (16) Unclassified, 1.54%, (21) ND, 11.18%, (152)	10.43 days (4.07) (3.6–21.1) ND (178)	7.32 DA (4.07) (2.8–17.1) ND (178)	847.0 μm (153.0) ND (235)	338.0 μm (15.0) ND (235)	NC, 94.7%, (1.287) ND (72)	NC, 82.11%, (1.116) VH, 8.16%, (101) REBLEED, 3.17%, (48) RPE TEAR, 0.12%, (4) RRD, 1.08%, (15) ENDOPHTHALMITIS, 0.24%, (3) ND, 5.29%, (72)
IVI anti‐VEGF + Gas	698 (271)	74.68 (6.38) ND (34)	M, 48.33%, (131) F, 30.25%, (82) ND (58)	AMD, 44.64%, (121) PCV, 52.76%, (143) RAM, 0.36%, (1) Unclassified, 1.47%, (4) ND, 0.73%, (2)	8.03 days (3.77) (1–16) ND (34)	6.33 DA (2.58) (2.4–10.5) ND (34)	595.1 μm (181.2) ND (34)	255.2 μm (83.38) ND (34)	NC, 94.1%, (255) ND (16)	NC, 83.39%, (226) VH, 6.27%, (17) REBLEED, 1.10%, (3) RPE TEAR, 2.95%, (8) RRD, 0.36%, (1) ND, 5.90%, (16)
IVI Gas	1.028 (400)	69.90 (6.87) ND (122)	M, 49.75%, (199) F, 34.5%, (138) ND (63)	AMD, 50.5%, (202) PCV, 31.5%, (126) RAM, 9.5%, (38) Blunt Trauma, 1.25%, (5) Myopia, 0.5%, (2) Unclassified, 3.5%, (14) ND, 3.25%, (13)	9.04 days (5.70) (4–21.8) ND (23)	8.67 DA (5.47) (3–17.9) ND (23)	488.1 μm (261.8) ND (85)	222.6 μm (10.54) ND (85)	NC, 90.5%, (362) HTO, 3.34%, (7) ND (31)	NC, 87.75%, (351) VH, 10.0%, (40) REBLEED, 0.50%, (2) RPE TEAR, 0.25%, (1) RRD, 1.00%, (4) ND, 0.25%, (1) HTO, 0.25%, (1)
ITV rTPA + anti‐VEGF + Gas	518 (379)	75.11 (6.59) ND (32)	M, 45.38%, (172) F, 54.61%, (126) ND (81)	AMD, 63.85%, (242) PCV, 31.39%, (119) RAM, 0.79%, (3) Unclassified, 3.95%, (15)	7.90 days (3.57) (3–14.3) ND (71)	7.63 DA (3.39) (2.6–15.4) ND (71)	565.3 μm (136.8) ND (83)	269.3 μm (105.0) ND (83)	NC, 97.6%, (370) HTO, 2.37%, (9)	NC, 64.37%, (244) VH, 5.27%, (20) REBLEED, 2.63%, (10) RPE TEAR, 0.79%, (3) RRD, 0.79%, (3) ND, 31.22%, (99)
IVI rTPA	110 (46)	77.70 (7.00)	M, 28.3%, (13) F, 71.7%, (33)	AMD, 100%, (46)	10.0 days (2.40) (5–18)	12.5 DA (6.75) (1–38)	ND	ND	NC, 93.4%, (43) HTO, 6.97%, (3)	NC, 93.02%, (43) VH, 6.97%, (3)
IVI Gas + PDT	6 (6)	53.67 (6.31)	M, 66.0%, (4) F, 34.0%, (2)	AMD, 66.0%, (4) PCV, 34.0%, (2)	ND	ND	ND	ND	NC, 100%, (6)	NC, 66.00%, (4) REBLEED, 34.0%, (2)
Total	6.112 (3.990)	72.67 (8.23)	M, 46.49%, (1.855) F, 43.65%, (1.742)	AMD, 66.09%, (2.637) PCV, 18.84%, (752) RAM, 2.33%, (93) Blunt Trauma, 1.17%, (47) Myopia, 0.45%, (18) Unclassified, 2.15%, (86) ND, 8.94%, (357)	11.01 days (6.29) (1–35)	7.28 DA (4.05) (1–38)	641.1 μm (226.5)	293.4 μm (74.51)	NC, 99.5%, (3.971) HTO, 0.47%, (19)	NC, 78.79%, (3.144) VH, 5.76%, (230) REBLEED, 4.28%, (171) RPE TEAR, 1.07%, (43) RRD, 0.57%, (23) HTO, 0.02%, (1) ENDOPHTHALMITIS, 0.07%, (3) ND, 9.39%, (375)
Vitrectomy therapies										
PPV + IVI rTPA + Gas	63 (63)	81 (9.87)	ND (63)	AMD, 100%, (63)	7.05 days (2.89) (5–9.1)	4.75 DA (0.35) (4.5–5)	786.0 μm (282.6)	347.5 μm (99.70.)	NC, 100%, (63)	NC, 79.36%, (50) VH, 4.76%, (3) RRD, 4.76%, (3) CATARACT, 11.1%, (7)
PPV + IVI rTPA + anti‐VEGF + Gas	87 (49)	80 (9.47)	M, 6.12%, (3) F, 30.6%, (15) ND (31)	AMD, 93.87%, (46) RAM, 6.12%, (3)	9.1 days (4.6) (−) ND (7)	4.68 DA (2.8) (3.2–8.1)	642.0 μm (322) ND (18)	418 μm (364) ND (18)	NC, 100%, (49)	NC, 57.14%, (28) VH, 8.16%, (4) REBLEED, 16.32%, (8) RRD, 4.08%, (2) CATARACT, 14.28%, (7)
PPV subretinal rTPA ± Gas ± anti‐VEFG	3.388 (2.196)	76.13 (6.40) ND (37)	M, 31.76%, (699) F, 44.14%, (970) ND (527)	AMD, 83.04%, (1.825) PCV, 8.04%, (176) RAM, 1.23%, (27) Blunt Trauma, 4.52%, (99) Myopia, 0.91%, (20) Unclassified, 0.91%, (20) ND, 1.32%, (29)	10.73 days (5.91) (2–25) ND (305)	8.49 DA (5.23) (3.4–23.2) ND (305)	717.2 μm (282.4) ND (1.003)	416.0 μm (134.2) ND (1.003)	NC, 97.16%, (2.134) MH, 0.27%, (6) HTO, 0.13%, (3) IRB, 0.59%, (13) ND, 1.82%; (40)	NC, 62.65%, (1.379) VH, 4.06%, (89) REBLEED, 11.06%, (242) RPE TEAR, 1.05%, (23) RRD, 4.15%, (91) HTO, 0.91%, (20) CATARACT, 1.82%, (40) CD, 0.13%, (3) MH, 1.6% (35) CNV, 0.18%, (4) ERM, 0.36%, (8) HYPHEMA, 0.18%, (4) ND, 11.79%; (258)
PPV + removal of SMH + Gas	224 (125)	71.24 (10.23)	M, 27.2%, (34) F, 28.8%, (36) ND (55)	AMD, 96.8%, (121) PCV, 3.2%, (4)	17.05 days (1.68) (9–23) ND (41)	9.75 DA (3.35) (6.5–12) ND (63)	871.0 μm (182.6) ND (90)	214.5 μm (79.70.) ND (90)	NC, 98.4%, (123) HTO, 0.8%, (1) IRB, 0.8% (1)	NC, 80.8%, (101) VH, 4.8%, (6) REBLEED, 4.0%, (5) HYPOTENSION, 2.4% (3) RRD, 3.2%, (4) MH, 1.6% (2) CATARACT, 3.2%, (4)
PPV + Subretinal PSS + Gas	10 (10)	61.20 (16.33)	M, 50.0%, (5) F, 50.0%, (5)	AMD, 30.0%, (3) PCV, 40.0%, (4) RAM, 20.0%, (2) Unclassified, 10.0%, (1)	8.09 days (3.17) (5–14)	5.75 DA (4.56) (2.5–20.3)	ND	ND	NC, 100%, (10)	NC, 50.0%, (5) VH, 30.0%, (3) RRD, 10.0%, (1) MH, 10.0%, (1)
Total	3.772 (2.443)	75.96 (7.02)	M, 30.33%, (741) F, 41.99%, (1.026)	AMD, 84.2%, (2.058) PCV, 7.53%, (184) RAM, 1.30%, (32) Blunt Trauma, 4.05%, (99) Myopia, 0.81%, (20) Unclassified, 0.85%, (21) ND, 1.18%, (29)	10.79 days (5.88) (2–25)	8.33 DA (5.13) (2.5–23.2)	721.6 μm (280.68)	404.4 μm (140.29)	NC, 97.3%, (2.379) MH, 0.24%, (6) IRB, 0.57%, (14) HTO, 0.16%, (4) ND, 1.64%; (40)	NC, 64.01%, (1.563) VH, 4.29%, (105) REBLEED, 10.43%, (255) RPE TEAR, 0.95%, (23) RRD, 4.13%, (101) HTO, 0.82%, (20) CATARACT, 2.37%, (58) CD, 0.12%, (3) CNV, 0.16%, (4) MH, 1.57%, (38) ERM, 0.32%, (8) HYPHEMA, 0.16%, (4) HYPOTENSION, 0.12% (3) ND, 10.69%; (258)

Abbreviations: AMD, age macular degeneration; CD, choroidal detachment; CNV, choroidal neovascularization; CRT, central retinal thickness; DA, disk area; F, female; HTO, hypertension; IRB, iatrogenic retinal breaks; IVI, intravitreal; M, male; MH, macular hole; NC, non complications; ND, non declared; PCV, polypoidal choroidal vasculopathy; PPV, pars plana vitrectomy; PSS, physiological saline solution; RAM, retinal artery macroaneurysm; RRD, rhegmatogenous retinal detachment; SD, standard deviation; SMH, submacular haemorrhage; TPA, tissue plasminogen activator; VEGF, vascular endothelial growth factor; VH, vitreous haemorrhage.

^a^ Combined mean and standard deviation (SD).

Table S1 shows a qualitative summary of all the studies included in the systematic review. The countries with the highest number of studies published on this topic were Japan (16.67%), Germany (11.33%) and the USA (10%). Most studies were retrospective case series. The levels of evidence measured by GRADE showed that 37.33% of the included studies had a very low level of certainty, 54.67% had a low level of certainty, and 8% had a moderate‐high level of certainty.

**TABLE 3:** Synthesis of the literature from 2004 to 2022.

Treatment	*N* (eyes)	Complete haemorrhage resolution after treatment, %, (*N*)	Complete haemorrhage resolution final visit, %, (*N*)	Any additional treatmets, %, (*N*)	Further anti‐VEGF injections	Mean number of injections, (SD)
Non‐vitrectomy therapies						
Observation	26 (26)	20.68% (6)	73.07% (19)	VPP, 11.53%, (3)	ND	ND
IVI anti‐VEGF	1.872 (1.503)	42.98% (622) ND (55)	78.89% (1.142) ND (55)	VPP, 2.46%, (37) LASER, 0.13%, (2) PDT 0.1%, (2) ND (153)	YES	5.24 (2.35)
IVI rTPA + Gas	1.854 (1.359)	63.94% (797) ND (112)	75.36% (939) ND (112)	VPP, 4.04%, (55) LASER, 0.14%, (2) TTT, 0.22% (3) ND (168)	YES	2.93 (0.71)
IVI anti‐VEGF + Gas	698 (271)	64.85% (186)	82.84% (224)	VPP, 7.74%, (21) ND (15)	YES	3.31 (1.40)
IVI Gas	1.028 (400)	60.28% (238) ND (5)	79.90% (315) ND (5)	VPP, 7.0%, (28) PDT, 1.75%, (7) ND (9)	YES	2.13 (1.10)
ITV rTPA + anti‐VEGF + Gas	518 (379)	62.64% (236) ND (1)	77.44% (292) ND (1)	VPP, 4.22%, (16) ND (5)	YES	2.29 (1.55)
IVI rTPA	110 (46)	13.04% (6)	ND	ND	ND	ND
IVI Gas + PDT	6 (6)	83% (5)	100% (6)	PDT, 100%, (6)	ND	ND
Total	6.112 (3.990)	54.91% (2.096) ND (173)	77.78% (2.937) ND (219)	VPP, 9.04%, (160) PDT, 0.32%, (13) LASER, 0.10%, (4) TTT, 0.07% (3) ND (396)	YES	4.04 (2.33)
Vitrectomy therapies						
PPV + IVI rTPA + Gas	63 (63)	44.0%, (27)	60.0%, (37)	VPP, 9.52%, (6)	YES	2.67 (0.57)
PPV + IVI rTPA + anti‐VEGF + Gas	87 (49)	67.34 (33)	89.79 (44)	VPP, 8.16%, (4)	YES	7.0 (1.13)
PPV subretinal rTPA ± Gas ± anti‐VEFG	3.388 (2.196)	64.90%, (1.326) ND (144)	70.13%, (1.433) ND (144)	VPP, 6.05%, (133) PDT, 0.18%, (4) LASER, 0.04%, (1) ND (423)	YES ND (34)	3.38 (1.42)
PPV + removal of SMH + Gas	224 (125)	60.0%, (75) ND (50)	88.0%, (110) ND (15)	VPP, 5.6% (7) ND (25)	YES	3.68 (1.34)
PPV + Subretinal PSS + Gas	10 (10)	90.0% (9)	ND	VPP, 10% (1)	ND	ND
Total	3.772 (2.443)	60.17% (1.470)	66.47% (1.624)	VPP, 6.18%, (151) PDT, 0.16%, (4) LASER, 0.04%, (1) ND (448)	YES	3.45 (1.49)

Abbreviations: IVT, intravitreal; PPV, pars plana vitrectomy; PSS, physiological saline solution; SD, std. deviation; SMH, submacular haemorrhage; TPA, tissue plasminogen activator; TTT, transpupillary thermotherapy; VEGF, vascular endothelial growth factor.

### 3.3 Overall results of network meta‐analysis

The overall results of the network meta‐analysis revealed differences in the effectiveness of the various interventions for SMH. Given the clinical heterogeneity across studies, a detailed overview of the specific characteristics of each intervention is essential for contextualising the findings. Table 4 provides a comprehensive synthesis of the clinical data associated with each included study.

**TABLE 4:** Characteristics and treatment protocols in studies on SMH.

Author	Treatment	Patients	Aetiology	Types of SMH	Sex	Age (mean ± standard desviation)	Time to treatment in days (mean ± sd)	Follow‐up time in months (mean ± sd)
Barayev et al. (2024)	SF6/C3F8 0.4–0.5 mL + rtPA 25 μg/0.1 ml (IVit)	25	AMD	Unspecified	12 M, 13F	88.0 ± 7.04 [IQR 81.5, 91.0]	0 ± 1.1 [IQR 0, 1.50]	2.8 ± 2.3 years (IQR: 1.7–4.8)
	PPV + SF6 0.4–0.5 mL	18	AMD		8 M, 10F	88.5 ± 9.04 [IQR 83.0, 95.2]	1.00 ± 2.96 [IQR 0, 4.00]	
Barzelay et al. (2024)	SF6 0.5 mL + rtPA 50 μg/0.1 mL (IVit)	42	AMD	Unspecified	25 M, 17F	83.33 ± 6.51	4.92 ± 4.55	12
	PPV + rtPA 25 μg/0.1 mL (SRet)	55	AMD		33 M, 22F	81.67 ± 7.46	3.79 ± 4.27	
Bell et al. (2017)	C3F8 0.3 mL + rtPA 25 μg/0.1 mL (IVit)	18	16 AMD, 1 RAM, 1 Myopic CNV	Unspecified	Unspecified	74.7 ± 14.9	<14	12
	PPV + rtPA 12.5 μg/0.1 mL (SRet)	14	12 AMD, 2 RAM			76 ± 6.2		
Boral et al. (2023)	PPV + rtPA 100 μg/0.1 mL (SRet) + Ranibizumab 1 mg/0.1 mL + SF6	62	nAMD or PCV	Subretinal haemorrhage	58 M, 45F	64.06 ± 10.18	<4 weeks	11.12 ± 6.13
	PPV + rtPA 100 μg/0.1 mL (SRet)	31				66.55 ± 10.02	4–8 weeks	
	ARCPGT	10				69.1 ± 8.85	>8 weeks	
Cakir et al. (2010)	C3F8 0.4–0.5 mL	14	10 AMD, 1 Myopic CNV, 2 RAM	Subretinal haemorrhage	5 M, 9F	68.6 ± 7.5	5.8 ± 0.8	7.2 ± 0.4 (range: 6–12)
	C3F8 0.4–0.5 mL + rtPA 25 μg/0.1 mL (IVit)	7	6 AMD, 1 Traumatic SRH		2 M, 5F	64.4 ± 3.9	4.7 ± 0.9	7.1
Caporossi et al. (2022)	PPV + rtPA 5 μg/0.1 mL	22	AMD	Subretinal haemorrhage	11 M, 11F	80.05 ± 6.51	25.9 ± 36	12
	PPV + AM	22	AMD		11 M, 11F	78.76 ± 6.13	27.64 ± 32.09	
Cho et al. (2020)	Observation	5	RAMs	Subretinal haemorrhage	2 M, 3F	66.20 ± 10.89	Unspecified	6–24
	Bevacizumab 2.5 mg/0.1 mL (IVit)	13	RAMs		5 M, 8F	71.62 ± 12.12	Unspecified	
Cho et al. (2015)	Ranibizumab 1 mg/0.1 mL (IVit)	58	27 AMD, 31 PCV	Unspecified	33 M, 25F	68.1 ± 7.5	8.5 ± 5.9	12
	SF6/C3F8 0.1–0.4 mL + Ranibizumab 1 mg/0.1 mL (IVit)	35	15 AMD, 20 PCV		20 M, 15F	65.3 ± 8.9	7.9 ± 5.7	
Fang et al. (2009)	SF6/C3F8 0.3–0.4 mL	25	AMD	Subretinal haemorrhage	16 M, 9F	67.64 ± 2.19	21.80 ± 3.81	10.80 ± 0.68
	SF6/C3F8 0.3–0.4 mL + rtPA 25–50 μg/0.1 mL (IVit)	28	AMD		15 M, 13F	64.86 ± 2.05	26.82 ± 5.06	12.82 ± 0.96
Fassbender et al. (2016)	C3F8 0.3 mL	9	AMD	Unspecified	1 M, 8F	79 ± 3	6 ± 2.2	14 ± 8
	C3F8 0.3 mL + rtPA 5 μg/0.1 mL (IVit)	10	AMD		6 M, 5F	80 ± 7	6 ± 4.2	12 ± 5
	PPV + rtPA 5 μg/0.1 mL (SRet) + SF6	18	AMD		5 M, 13F	83 ± 8	5 ± 4.6	12 ± 3
Fujikawa et al. (2013)	C3F8 0.4 mL	30	8 AMD, 17 PCV, 5 RAM	Unspecified	18 M, 12F	65.5 ± 8.1	8.2 ± 5.6	Up to 6 years
	C3F8 0.3 mL + rtPA 25 mg/0.1 mL	38	6 AMD, 27 PCV, 5 RAM		22 M, 16F	70.2 ± 9.5	8.2 ± 4.9	
Gabrielle et al. (2023)	SF6 0.3 mL + Ranibizumab 1 mg/mL + rtPA 10 μg/0.1 mL (IVit)	45	AMD	Subretinal and subRPE haemorrhage	19 M, 26F	82.29 ± 8.0	7.4 ± 4.7	6
	PPV + rtPA 10 μg/0.1 mL (SRet) + Ranibizumab 1 mg/mL + SF6 0.3 mL	45	AMD		45 M, 33F	84.3 ± 8.3	7.6 ± 5.1	
Grohmann et al. (2020)	C2F6 IVit + Bevacizumab 1.25 mg + rtPA 20 μg/0.1 mL	32	AMD	Subretinal haemorrhage	32 M, 53F	85.36 ± 36.3 (range 53–102)	Unspecified	6.1 (range 5–7)
	PPV + rtPA 20 μg/0.1 mL (SRet) + Bevacizumab 1.25 mg + C2F6	42					Unspecified	
	PPV + rtPA 20 μg/0.1 ml (IVit) + Bevacizumab 1.25 mg + C2F6	11					Unspecified	
Gujral et al. (2019)	C3F8 0.3 mL	4	Blunt trauma	Subretinal haemorrhage	4 M	32.25 ± 5.61	5.5 ± 1.91	6
	C3F8 0.3 mL + rtPA 100 mg/0.1 mL (IVit)	15	Blunt trauma		10 M, 5F	27.93 ± 7.72	10.29 ± 7.14	
	PPV + rtPA 25 μg/0.1 mL (SRet) + C3F8 0.3 mL	1	Blunt trauma		1 M	55 ± 0	30 ± 0	
Guthoff et al. (2011)	SF6 0.3–0.5 mL + Bevacizumab 25 mg/mL (IVit) + rtPA 200 mg/mL	12	AMD	Subretinal haemorrhage	5 M, 7F	81 ± 5.2	11.25 ± 6.23	7
	SF6 0.3–0.5 mL + rtPA 200 mg/mL (IVit)	26	AMD		4 M, 22F	83 ± 6.3	10.9 ± 8.89	
Hesgaard et al. (2012)	C3F8 0.3 mL + Ranibizumab 0.5 mg (IVit)	8	AMD	Unspecified	8F	84.9 ± 6	5.25 ± 8.88	11.65 ± 3.89
	Ranibizumab 0.5 mg (IVit)	7	AMD		3 M, 4F	81.2 ± 7.74	13.29 ± 5.53	14.93 ± 8.23
Inoue et al. (2022)	SF6/C3F8 ± tPA	49	32 AMD, 94 PCV, 1 RAP	Subretinal haemorrhage	88 M, 39F	74.2 ± 9.2	Unspecified	18.1 ± 12.1
	Aflibercept/Ranibizumab/Bevacizumab	42					Unspecified	
	Vitrectomy ± tPA	36					Unspecified	
Jeong et al. (2020)	Aflibercept/Ranibizumab/Bevacizumab (IVit)	29	AMD	Subretinal haemorrhage	54 M, 23F	72.87 ± 10.47	25 ± 37.7	18.3 ± 18.5
	C3F8 0.3 mL + Bevacizumab/Ranibizumab/Aflibercept (IVit)	25	AMD				10.27 ± 11.3	15.87 ± 16.1
	PPV + rtPA (SRet) + SF6	23	AMD				10.07 ± 17.6	19.8 ± 19.1
Kang et al. (2018)	Bevacizumab (IVit)	22	PCV	Subretinal haemorrhage	13 M, 9F	68.0 ± 9.3	5.5 ± 9.4	30.8 ± 32.1
	C3F8 0.3–0.5 mL + Bevacizumab Ivit	14	PCV		9 M, 5F	64.9 ± 8.0	1.0 ± 1.7	31.2 ± 28.9
	SF6 + Bevacizumab IVit + tPA 50 μg/0.1 mL	12	PCV		7 M, 5F	61.7 ± 8.1	5.3 ± 7.6	35.8 ± 40.4
Kishikova et al. (2021)	PPV + rtPA 25 μg/0.1 mL (SRet) + SF6	18	AMD	Subretinal and subRPE haemorrhage	Unspecified	78 ± 6	14	6
	SF6 0.3 mL + rtPA 50 μg in 0.1 ML (SRet)	11	AMD			78 ± 6	14	
Kitahashi et al. (2014)	SF6 0.3–0.5 mL	10	10 PCV	Unspecified	8 M, 2F	69.7 ± 8.7	6.1 ± 4.9	6
	SF6 0.3–0.5 mL + Bevacizumab 1.25 mg	22	22 PCV		17 M, 5F	66.9 ± 8.9	8.1 ± 3.7	
Lee et al. (2021)	C3F8 0.3 mL + Bevacizumab/Ranibizumab/Aflibercept + rtPA 100 μg/0.01 mL (IVit)	32	9 AMD, 23 PCV	Unspecified	17 M, 14F	68.2 ± 11.0	13.5 ± 12.1	6
	Aflibercept/Ranibizumab/Bevacizumab	50	23 AMD, 25 PCV, 2 RAP		30 M, 20F	72.9 ± 10.4	12.2 ± 10.3	
Mayer et al. (2013)	SF6 0.4 mL + Bevacizumab 25 mg/mL (IVit)	13	AMD	Unspecified	7 M, 6F	83.4	<8	12
	SF6 0.4 mL + rtPA 100 μg/0.1 mL	32	AMD		14 M, 18F	79.2	<8	
Mizutani et al. (2011)	SF6 0.3–0.6 mL	13	39 AMD, 14 RAM	Unspecified	Unspecified	72.6 ± 10.2 (range, 50–90)	Unspecified	18.4 ± 16.6 (range: 3–61)
	SF6 0.3–0.6 mL + rtPA 40 kUI (IVit)	50						
Mun et al. (2022)	Observation	21	AMD	Unspecified	17 M, 4F	66.73 ± 10.69	Unspecified	12 ± 0
	Aflibercept 2 mg/Ranibizumab 0.5 mg/Bevacizumab 1.25 mg	161	AMD		102 M, 59F	70.17 ± 10.39	8.56 ± 17.7	9.6 ± 3.29
	SF6 (0.5 mL)/C3F8 (0.25 mL) ± Bevacizumab 1.25 mg/Ranibizumab 0.5 mg/Aflibercept 2 mg ± rtPA	31	AMD		22 M, 9F	69.74 ± 11.32	7.45 ± 8.76	10 ± 3.46
	rtPA (SRet) ± Bevacizumab 1.25 mg/Ranibizumab 0.5 mg/Aflibercept 2 mg	23	AMD		14 M, 9F	75.96 ± 9.25	11.1 ± 19.0	5.57 ± 3.21
Nourinia et al. (2010)	SF6 0.3 mL + Bevacizumab 1.25 mg	3	AMD	Subretinal haemorrhage	2 M, 1F	74.3 ± 8.1	7.33 ± 3.79	12
	SF6 0.3 mL + Bevacizumab 1.25 mg + rtPA 50 μg	2	AMD		1 M, 1F	77.5 ± 11.3	5 ± 2.83	
Papavasileiou et al. (2013)	C3F8 0.3 mL + rtPA 100 μg/0.1 mL + Ranibizumab 0.5 mg	7	AMD	Subretinal haemorrhage	4 M, 3F	79.43 ± 3.38	6.29 ± 1.98	19 ± 18.38
	C3F8 0.3 mL + rtPA 100 μg/0.1 mL + PDT	2	IPCV		2F	62.5 ± 13.44	5 ± 2.82	14 5 ± 10.07
Rickmann et al. (2021)	PPV + rtPA 100 μg/0.1 mL (IVit)/rtPA 10 μg/0.1 mL (SRet) + Gas	17	AMD	Subretinal and subRPE haemorrhage	6 M, 11F	81.7 ± 5.2	3.4 ± 1.5	3
	PPV + rtPA 100 μg/0.1 mL (IVit)/rtPA 10 μg/0.1 mL (SRet) + Bevacizumab 1.25 mg + Gas	14	AMD		4 M, 10F	83.4 ± 4.6	3.3 ± 1.6	
Rishi et al. (2012)	C3F8 0.3 mL	7	1 AMD, 1 Trauma, 4 PCV, 1 RAM	Subretinal haemorrhage	31 M, 15F	53.14 ± 22.1	8 ± 6.67 [IQR: 9]	2.5 ± 16.30
	C3F8 0.2–0.3 mL + rtPA 50 μg/0.1 mL (IVit)	25	15 AMD, 7 Trauma, 1 High myopia, 1 PCV, 1 RAM			53.64 ± 15.8	10 ± 8.89 [IQR: 12]	6.5 ± 17.04
	PPV + rtPA 10 μg/0.1 mL (SRet)	14	11 AMD, 2 Trauma, 1 High myopia			55.57 ± 15.3	8 ± 7.41 [IQR: 10]	31.5 ± 31.85
Tiosano et al. (2023)	SF6 + rtPA 25 μg/0.1 mL (SRet)	51	41 AMD, 9 RAM, 1 myopic CNV, 1 central retinal vein occlusion; 3 no aetiology identified	Subretinal haemorrhage	26 M, 25F	80.24 (IQR 75.09, 86.75)	0 ± 0.93 [0–1.25]	3.28 years ±31.85 months [IQR: 1.81–4.54 years]
	PPV + rtPA 25 μg/0.1 mL (IVit) + SF6/C3F8 0.4–0.5 mL	56	43 AMD, 3 CNV not related to AMD, 2 RAM, 1 trauma, 2 no aetiology identified		25 M, 31F	80.7 (IQR 74.76, 85.13)	1 ± 2.96 [0–4]	4.9 years ±31.85 months [IQR: 2.3–7.4 years]
Thompson and Sjaarda (2005)	PPV + Retinotomy + Neovascular membrane removal + Gas	27	AMD	Subretinal haemorrhage	19 M, 27F	78.8 ± 1.4	0.87 ± 0.17 months	2.92 ± 0.48 years
	PPV + rtPA 12.5/0.1 mL + Gas	15	AMD		9 M, 15F	82.5 ± 1.5	0.96 ± 0.25 months	2.30 ± 0.25 years
Tranos et al. (2021)	SF6/C3F8 + Bevacizumab 1.25 mg + rtPA (IVit)	11	AMD	Subretinal (14 eyes) and/or subRPE (6 eyes) haemorrhage	6 M, 5F	80 (range 60–96)	6.5 ± 3.7	12
	PPV + rtPA (SRet) + SF6/C3F8 + Bevacizumab 1.25 mg	14	AMD		8 M, 6F		7.5 ± 7.1	
Sandhu et al. (2010)	PPV + rtPA 12.5 μg/0.1 mL (SRet) + Gas + Ranibizumab 0.05 mg (IVit)	12	AMD	Subretinal and subRPE haemorrhage	6 M, 10F	81 (range 76–88)	15 (range: 3–42)	12
	PPV + rtPA 12.5 μg/0.1 mL (SRet) + Gas	4	AMD					
Sniatecki et al. (2021)	PPV + rtPA 100 μg/0.01 mL (IVit) + C3F8	36	AMD	Unspecified	12 M, 24 F	82 (range 63–97)	5 ± 8.88 (range 1–13)	12
	Aflibercept/Ranibizumab/Bevacizumab	18	AMD		4 M, 14 F	82 (range 63–94)		
Shin et al. (2015)	Ranibizumab 0.5 mg (IVit)/Bevacizumab 1.25 mg (IVit)	42	17 AMD, 25 PCV	Subretinal (40 eyes) and subRPE (11 eyes) haemorrhage	27 M, 15F	74.6 ± 6.8	13.8 ± 11.5	6
	SF6/C3F8 0.3 mL + Ranibizumab 0.5 mg (IVit)/Bevacizumab 1.25 mg (IVit)	40	21 AMD, 19 PCV	Subretinal (37 eyes) and subRPE (13 eyes) haemorrhage	26 M, 14F	72 ± 8.3	11.4 ± 10.4	
Szeto et al. (2024)	SF6/C3F8 0.3–0.4 mL	40	11 AMD, 29 PCV	Subretinal and subRPE haemorrhage	21 M, 19F	71.4 ± 10.8	5.53 ± 5.882	12
	PPV + rtPA 125 μg/mL (SRet) + Aflibercept 40 mg/mL/Ranibizumab 10 mg/mL (SRet)	23	13 AMD, 10 PCV		15 M, 8F	67.8 ± 11.4	10.65 ± 4.04	
Yang et al. (2005)	SF6/C3F8 0.3–0.4 mL	16	13 AMD, 1 RAM, 1 IPCV, 1 PDR	Subretinal haemorrhage	12 M, 4F	52.25 ± 16.83	12.31 ± 9.80	15.06 ± 10.90
	SF6/C3F8 0.3–0.4 mL + rtPA 25–33 μg/0.1 mL	8	5 AMD, 2 traumatic choroidal rupture, 1 RAM		7 M, 1F	65.31 ± 10.63	4.75 ± 4.10	16.38 ± 7.46
Ye et al. (2023)	PPV + rtPA 6.9 μg/0.1 mL (SRet)	16	PCV	Unspecified	Unspecified	Unspecified	<30	6
	PPV	16	PCV					

Abbreviations: AM, amniotic membrane; AMD, age‐related macular degeneration; ARCPGT, autologous retinal pigmentary retinal pigment epithelium‐choroid patch graft transplantations; C2F6, perfluoroethane; C3F8, perfluoropropane; CNV, choroidal neovascularization; F, female; IPCV, idiopathic polypoidal choroidal vasculopathy; IQR, interquartile range; IVit, intravitreal; M, male; PCV, polypoidal choroidal vasculopathy; PD, pneumatic displacement; PDR, proliferative diabetic retinopathy; PDT, photodynamic therapy; PPV, Pars Plana vitrectomy; RAM, retinal arterial macroaneurysm; RAP, retinal angiomatous proliferation; rtPA, recombinant tissue plasminogen activator; SD, standard deviation; SMH, submacular haemorrhage; SRet, subretinal; subRPE, sub‐retinal pigment epithelium haemorrhage.

**FIGURE 4:**
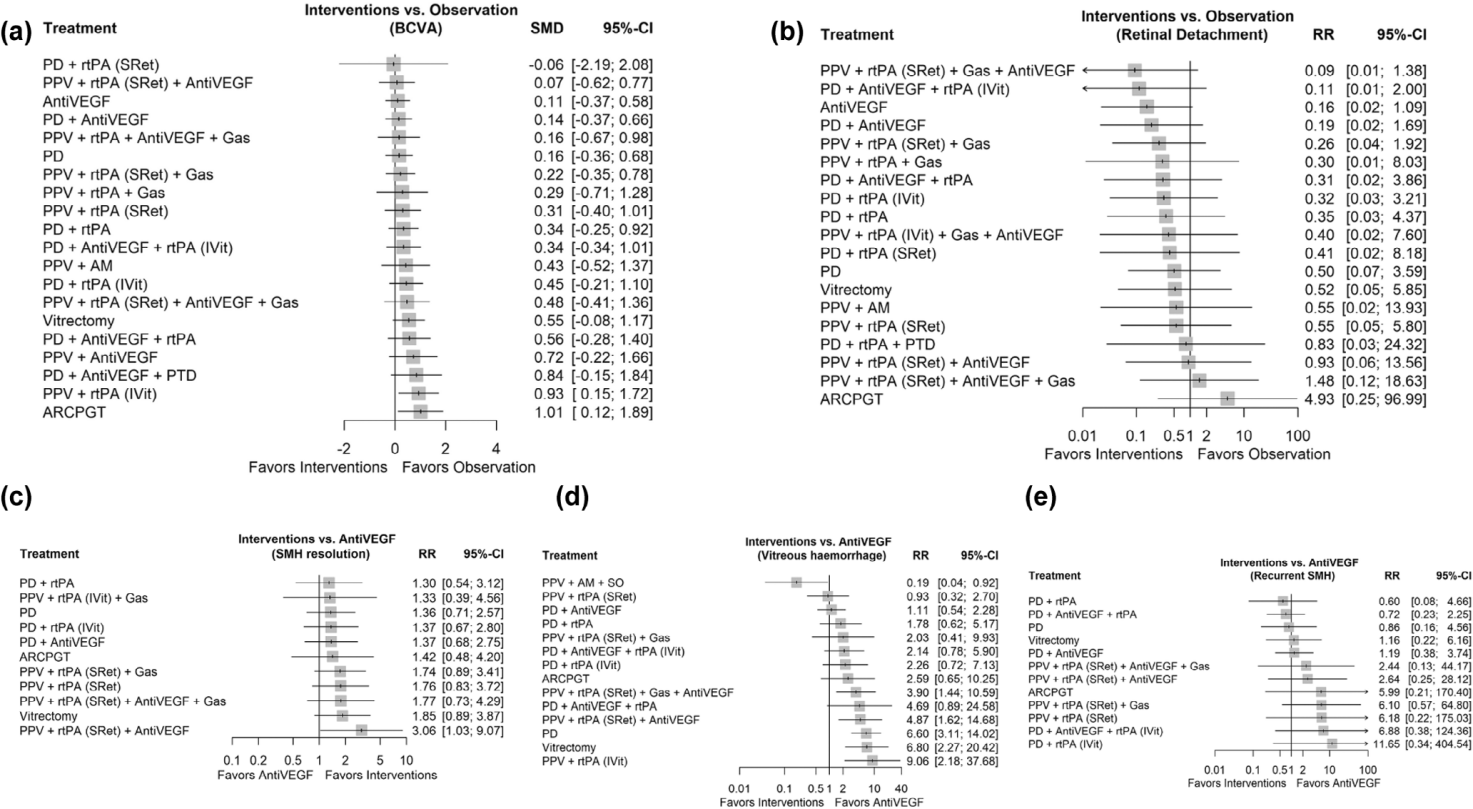
Forest plot summering the effects of different interventions. (a) Standardized mean differences of BCVA comparing interventions with observation. (b) Relative risk of RD comparing interventions with observation. (c) Relative risk of SMH resolution comparing interventions with antiVEGF therapy. (d) Relative risk of VH comparing interventions with antiVEGF therapy. (e) Relative risk of recurrent SMH comparing interventions with antiVEGF therapy. The ‘observation’ group includes only two studies (26 eyes), one of which permitted rescue vitrectomy in 38% of cases and the other included patients with macroaneurysms. This heterogeneous nature limits its classification as a pure observation arm. AM, amniotic membrane; AntiVEGF, anti‐vascular endothelial growth factor; ARCPGT, autologous retinal pigmentary retinal pigment epithelium‐choroid patch graft transplantations; BCVA, best corrected visual acuity; IVit, intravitreal injection; PD, pneumatic displacement; PPV, pars plana vitrectomy; PTD, photodynamic therapy; RD, retinal detachment; rtPA, recombinant tissue plasminogen activator; SMD, standardized mean difference; SRet, subretinal; VH, vitreous hemorraghe; 95% CI, 95% confidence interval.

The primary BCVA results in terms of logMAR at the end of follow‐up are shown in Figure 4a. The *I* ^2^ estimator for the random‐effects model showed moderate heterogeneity across studies (*I* ^2^: 28.9% [95% CI: 0.0%—61.7%]). In addition, consistency within the different treatment comparison designs showed no significant evidence of heterogeneity (*p* = 0.4396). On the other hand, network consistency showed a slight tendency towards inconsistency, although it was not statistically significant (*p* = 0.1140). None of the treatments tested proved to be more effective than observation. Estimates where the proportion of direct evidence for each network estimate is <60% show that the mean path length was greater than two (Figure S1). Direct and indirect comparisons of effect estimates for each of the interventions are shown in Table S2.

The results concerning the comparison of the different therapies for the resolution of SMH are reflected in Figure 4c. The *I* ^2^ estimator for the random effects model showed high heterogeneity between studies (*I* ^2^: 90.7% [95% CI: 85.0%–94.2%]). In addition, the network inconsistency between the different study designs was remarkable (*p* < 0.0001). The therapeutic combination of vitrectomy, subretinal rtPA and antiVEGF was superior in the resolution of SMH compared to antiVEGF monotherapy with a RR 3.0636 (95% CI: 1.0348–9.0700). Direct and indirect comparisons of effect estimates for each of the interventions are shown in Table S3. Estimates where the proportion of direct evidence for each network estimate is <0%, the mean path length was greater than two (Figure S2).

Comparisons of the different therapies for reported adverse events are shown in Figure 4b,d,e. Regarding the risk of RD, all therapies presented a statistically non‐significant risk compared to observation. However, the heterogeneity of studies with the same design and network inconsistency was significant (*p* < 0.0001). Furthermore, comparisons in which the direct evidence is <20%, the mean path length was greater than two (Figure S3). The risk of VH as an adverse event was significant for PD (RR: 6.6044 [95% CI: 3.1116–14.0181]) or more invasive procedures such as isolated vitrectomy (RR: 6.8029 [95% CI: 2.2660–20.4229]) or combined with: intravitreal rtPA (RR: 9.0649 [95% CI: 2.1806–37.6840]), subretinal rtPA and anti‐VEGF injection (RR: 4.8707 [95% CI: 1.6159–14.6816]), or subretinal rtPA and anti‐VEGF injection with PD (RR: 3.9031 [95% CI: 1.4381–10.5933]); all compared to anti‐VEGF monotherapy. Heterogeneity between the same designs and between studies was high (*I* ^2^ = 98.2% [95% CI: 97.6%–98.7%]). Estimates in which the proportion of direct evidence for each network estimate was <60%, the mean path length was greater than two (Figure S4). SMH recurrence showed no statistically significant differences between the different therapies and anti‐VEGF monotherapy. Network inconsistency and heterogeneity between studies was high (*I* ^2^ = 99.2% [95% CI: 98.9%–99.4%]). Estimates in which the proportion of direct evidence for each network estimate was zero, the mean path length was greater than two (Figure S5). All direct and indirect comparisons can be found in Tables S4–S6. The inconsistency of the direct and indirect estimates can be analysed in the forest plots of the Figures S6–S10.

**FIGURE 5:**
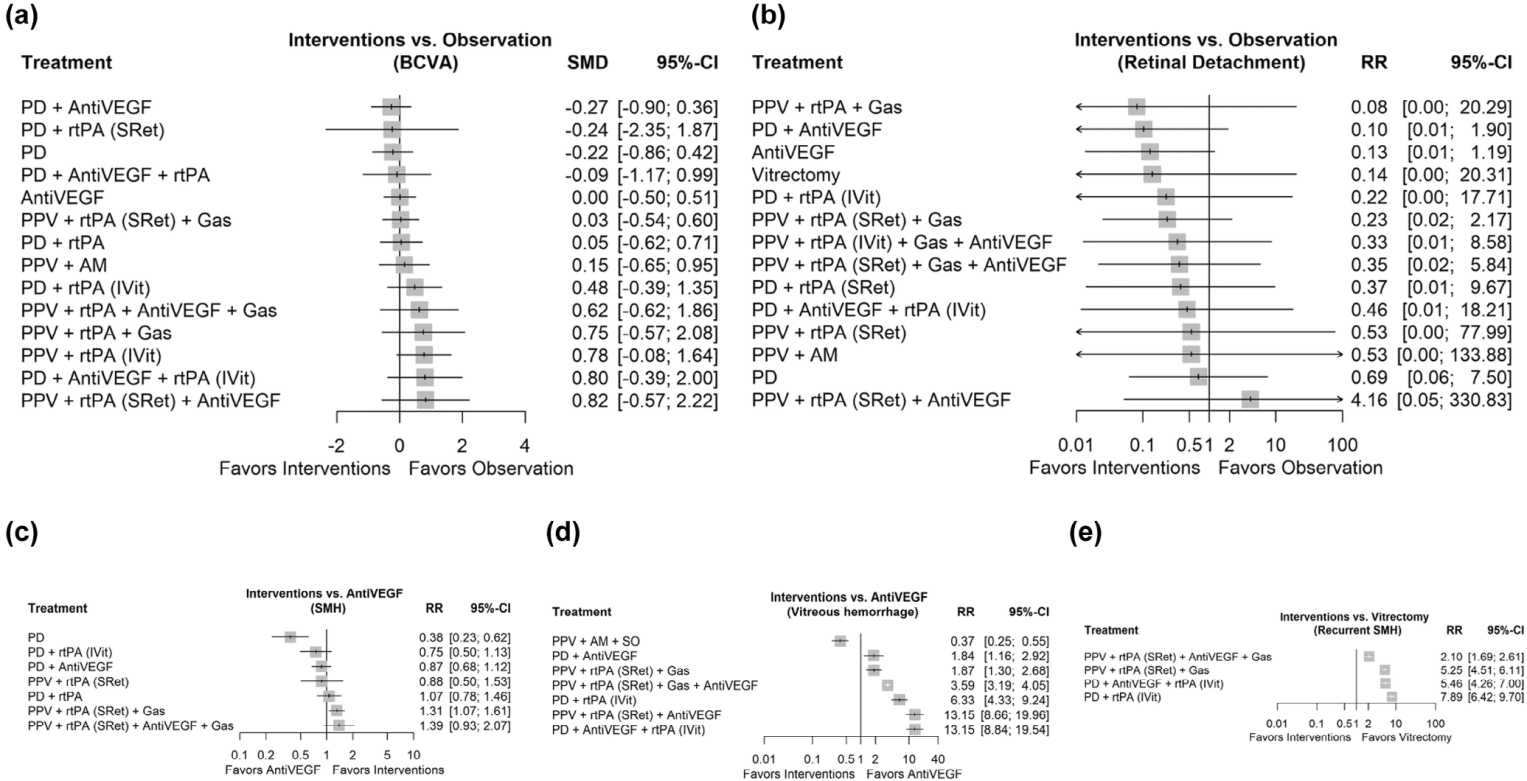
Forest plot summering the effects of different interventions for SMH secondary to AMD. (a) Standardized mean differences of BCVA comparing interventions with observation. (b) Relative risk of RD comparing interventions with observation. (c) Relative risk of SMH resolution comparing interventions with antiVEGF therapy. (d) Relative risk of VH comparing interventions with antiVEGF therapy. (e) Relative risk of recurrent SMH comparing interventions with antiVEGF therapy. The ‘observation’ group includes only one study (21 eyes), which permitted rescue vitrectomy in 38% of cases. This heterogeneous nature limits its classification as a pure observation arm. AM, amniotic membrane; AntiVEGF, anti‐vascular endothelial growth factor; ARCPGT, autologous retinal pigmentary retinal pigment epithelium‐choroid patch graft transplantations; BCVA, best corrected visual acuity; IVit, intravitreal injection; PD, pneumatic displacement; PPV, pars plana vitrectomy; PTD, photodynamic therapy; RD, retinal detachment; rtPA, recombinant tissue plasminogen activator; SMD, standardized mean difference; SRet, subretinal; VH, vitreous hemorraghe; 95% CI, 95% confidence interval.

Figure 5a presents the primary outcomes for BCVA in logMAR for the n‐AMD sub‐analysis at the end of follow‐up. The heterogeneity across the network was minimal (*I* ^2^ = 0%), and the design‐by‐treatment inconsistency test was not significant (*Q* = 0.09, df = 2, *p* = 0.96). Overall, no treatment demonstrated statistically significant superiority over observation in the network estimates. The findings related to the effectiveness for resolving SMH for the n‐AMD sub‐analysis are illustrated in Figure 5c. Notably, the treatment combination of PPV with subretinal injection of rtPA and PD demonstrated significantly better outcomes than Anti‐VEGF, with a RR of 1.31 (95% CI: 1.07–1.61, *p* = 0.01), suggesting superior visual outcomes. In contrast, the addition of Anti‐VEGF to this same regimen did not confer additional benefit (RR = 1.39, 95% CI: 0.93–2.07, *p* = 0.11). As part of the n‐AMD sub‐analysis, Figure 5b,d,e illustrate the comparisons between different therapies in relation to reported adverse events. In terms of RD risk, none of the treatments showed a statistically significant difference when compared to observation. Anti‐VEGF monotherapy demonstrated a relative risk of 0.1280 (95% CI: 0.0137–1.1928), with a *p*‐value of 0.071. However, there was notable heterogeneity among studies (*I* ^2^: 99.7%) with similar designs, and the network analysis revealed significant inconsistency (*p* < 0.0001). The risk of VH as an adverse event was significant for PD associated with intravitreal injection of rtPA and anti‐VEGF, RR of 13.15 (95% CI: 8.84–19.54). Also, the combination of PPV and subretinal injection of rtPA and anti‐VEGF showed significant risk of VH, RR of 13.15 (95% CI: 8.66–19.96). Conversely, PPV with amniotic membrane (AM) implantation demonstrated a significantly lower risk than anti‐VEGF monotherapy, with an RR of 0.37 (95% CI: 0.25–0.55; *p* < 0.0001). However, given the limited number of comparisons involving this treatment, these results should be interpreted with caution. SMH recurrence showed statistically significant differences between treatment pairs. For instance, the combination of PD with intravitreal injection of rtPA showed a markedly superior outcome compared to vitrectomy, with a RR of 7.89 (95% CI: 6.42–9.70; *p* < 0.0001). Similarly, PD associated with intravitreal injection of anti‐VEGF and rtPA was also highly effective, with an RR of 5.46 (95% CI: 4.26–7.00; *p* < 0.0001). Among the vitrectomy‐based interventions, PPV associated with subretinal injection of rtPA and PD demonstrated a strong effect with an RR of 5.25 (95% CI: 4.51–6.11; *p* < 0.0001). The more complex combination of PPV with subretinal injection of rtPA, intravitreal injection of anti‐VEGF and PD also showed a statistically significant benefit, with an RR of 2.10 (95% CI: 1.69–2.61; *p* < 0.0001). However, given the limited number of comparisons involving this treatment, these results should be interpreted with caution.

### 3.4 *P* score

**FIGURE 6:**
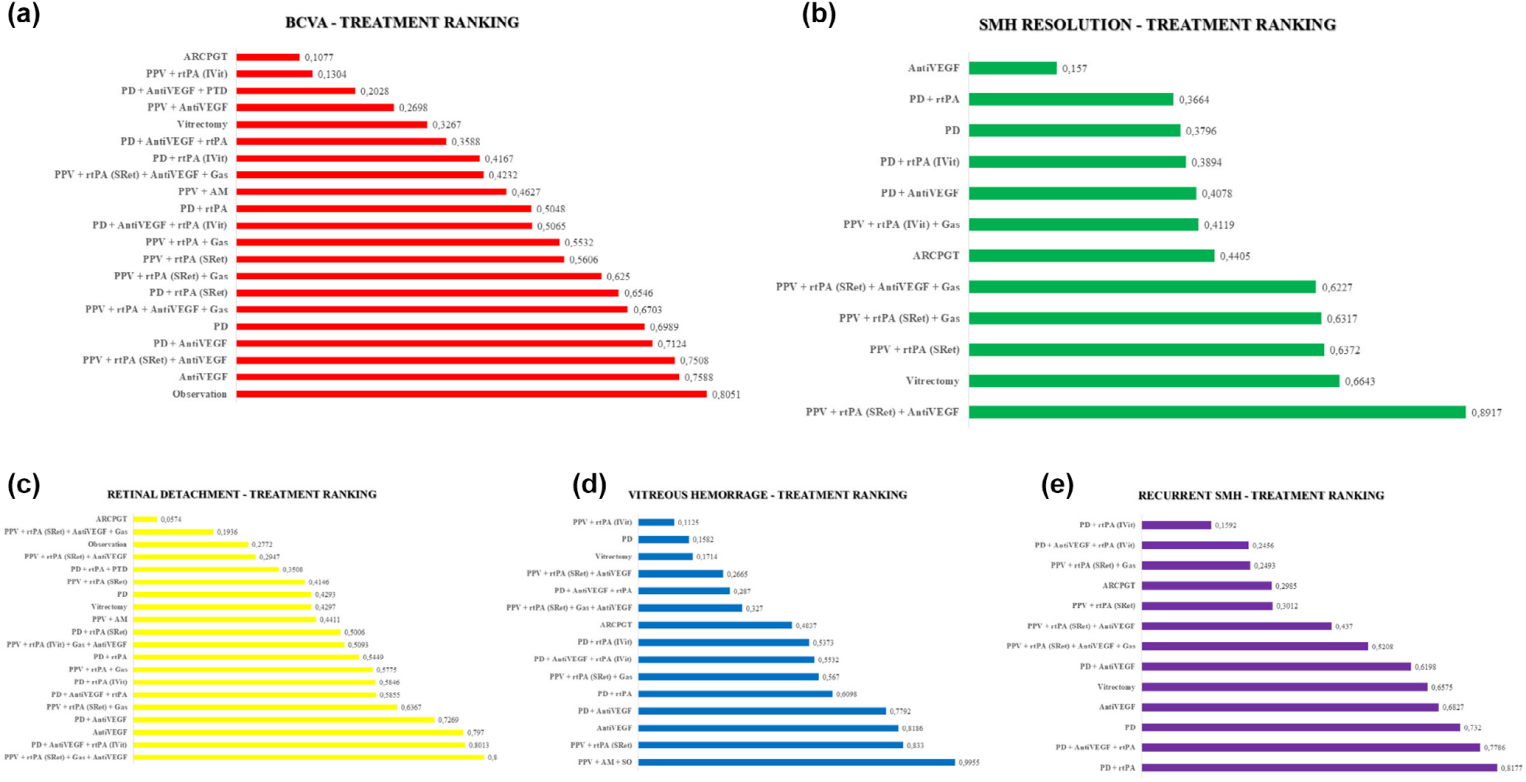
Ranking of treatments according to efficacy (a, BCVA; b, SMH resolution) and security (c, retinal detachment; d, vitreous hemorrage; e, recurrent SMH) for SMH treatment via *P*‐score. The ‘observation’ group includes only two studies (26 eyes), one of which permitted rescue vitrectomy in 38% of cases and the other included patients with macroaneurysms. This heterogeneous nature limits its classification as a pure observation arm. *P*‐score‐based rankings should be interpreted with caution, as they do not adjust for sample size, quality, or clinical heterogeneity. AM, amniotic membrane; AntiVEGF, anti‐vascular endothelial growth factor; ARCPGT, autologous retinal pigmentary retinal pigment epithelium‐choroid patch graft transplantations; IVit, intravitreal injection; PD, pneumatic displacement; PPV, pars plana vitrectomy; PTD, photodynamic therapy; RD, retinal detachment; rtPA, recombinant tissue plasminogen activator; SRet, subretinal; VH, vitreous hemorraghe.

The ranking of treatments assessed by *P*‐score can be found in Figure 6. The interventions that showed the greatest benefit for BCVA were observation (*P* score: 0.8051), antiVEGF intravitreal injection (*P* score: 0.7588), vitrectomy together with subretinal injection of rtPA and antiVEGF (*P* score: 0.7508) and PD with antiVEGF intravitreal injection (*P* score: 0.7124). The interventions showing the least benefit were the combined therapy of PD with antiVEGF and PDT (*P* score: 0.2028), vitrectomy together with subretinal injection of rtPA (*P* score: 0.1304) and autologous retinal pigment epithelium patch graft transplantation together with choroid (*P* score: 0.1077). The anatomic outcomes assessed by SMH resolution are in favour of more invasive therapies. Among these, PPV in combination with subretinal rtPA and antiVEGF (*P* score: 0.8917), vitrectomy alone (*P* score: 0.6643) or with other combinations stand out.

Therapies associated with the lowest risk of RD were PPV in combination with AM and silicone oil (*P*‐score: 0.9955), followed by PPV in combination with subretinal rtPA (*P*‐score: 0.8330). Among non‐vitrectomy options, antiVEGF monotherapy and the combination of PD and antiVEGF also showed favourable safety profiles, with *P*‐scores of 0.8186 and 0.7792, respectively. Regarding VH, the therapies with the lowest associated risk were PPV in combination with subretinal rtPA with PD and intravitreal injection of antiVEGF (*P*‐score: 0.8473), followed by PD in combination with intravitreal injection of antiVEGF and rtPA (*P*‐score: 0.8013) and antiVEGF monotherapy (*P*‐score: 0.7970). The combination of PD and antiVEGF also ranked favourably (*P*‐score: 0.7269). However, those therapies that showed lower risk of recurrent SMH were PD in combination with rtPA (*P* score: 0.8177), PD in combination with rtPA and antiVEGF (*P* score: 0.7786) and PD (*P* score: 0.7320).

**FIGURE 7:**
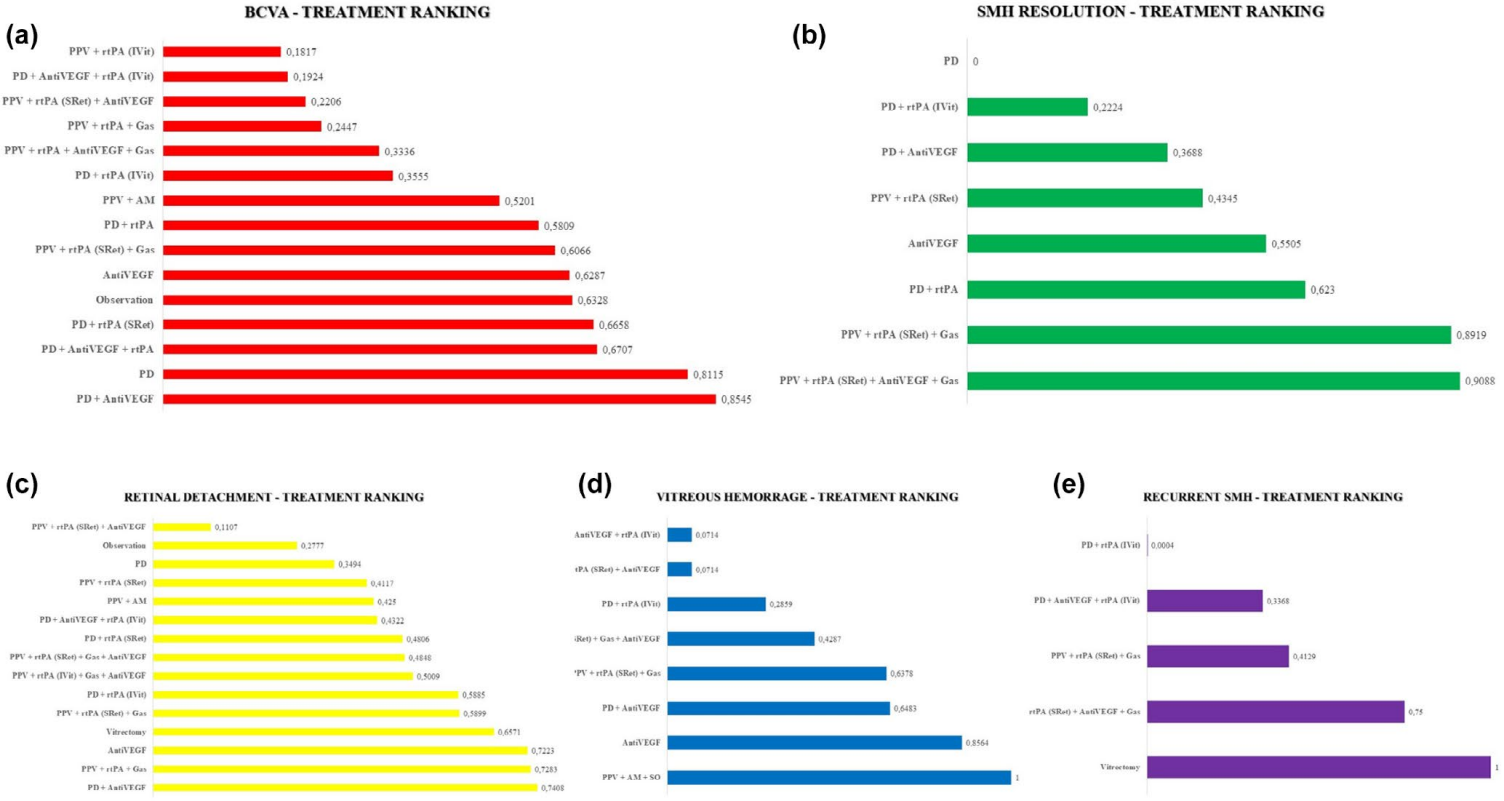
Ranking of treatments according to efficacy (a, BCVA; b, SMH resolution) and security (c, retinal detachment; d, vitreous hemorrage; e, recurrent SMH) for SMH secondary to n‐AMD treatment via *P*‐score. The ‘observation’ group includes only one study (21 eyes), which permitted rescue vitrectomy in 38% of cases. This heterogeneous nature limits its classification as a pure observation arm. *P*‐score‐based rankings should be interpreted with caution, as they do not adjust for sample size, quality, or clinical heterogeneity. AM, amniotic membrane; AntiVEGF, anti‐vascular endothelial growth factor; ARCPGT, autologous retinal pigmentary retinal pigment epithelium‐choroid patch graft transplantations; IVit, intravitreal injection; PD, pneumatic displacement; PPV, pars plana vitrectomy; PTD, photodynamic therapy; RD, retinal detachment; rtPA, recombinant tissue plasminogen activator; SRet, subretinal; VH, vitreous haemorrhage.

The ranking of treatments according to their effectiveness in improving BCVA for the n‐AMD sub‐analysis is presented in Figure 7. The highest *P*‐scores were observed for PD associated with antiVEGF (0.8545), PD alone (0.8115), and the combination of PD with injection of antiVEGF and rtPA (0.6707). In contrast, the lowest *P*‐scores were associated with more complex or invasive combinations such as the combination of PPV with intravitreal injection of rtPA (0.1817). When evaluating anatomical outcomes based on SMH resolution, more invasive surgical approaches appeared to be more effective. The highest *P*‐scores were observed for PPV associated with subretinal injection of rtPA, intravitreal injection of antiVEGF and PD (0.9088), and the combination of PPV with subretinal injection of rtPA and PD (0.8919), indicating strong performance in resolving SMH. While PD associated with intravitreal injection of rtPA (0.2224) and PD alone (0.0000) ranked lowest in this outcome domain. In terms of RD, the therapies associated with the lowest risk were PD associated with antiVEGF (*P*‐score: 0.7408), the combination of PPV with rtPA and PD (0.7283), and antiVEGF monotherapy (0.7223), all showing favourable safety profiles. In contrast, more complex combinations such as PPV associated with subretinal injection of rtPA and intravitreal injection of antiVEGF (0.1107) and the combination of PD with intravitreal injection of rtPA and antiVEGF (0.4322) ranked lower, suggesting a higher associated risk. For VH, the safest intervention was PPV with AM implantation (*P*‐score: 1.0000). The lowest *P*‐scores were seen with the combination of PD and intravitreal injection of antiVEGF and rtPA and PPV associated with subretinal injection of rtPA and intravitreal injection of antiVEGF (both 0.0714), indicating a higher risk of haemorrhagic complications. Regarding SMH recurrence, PPV alone ranked highest (*P*‐score: 1.0000), suggesting the lowest recurrence risk. PD‐based approaches such as PD associated with intravitreal injection of antiVEGF and rtPA (0.3368) and PD associated with intravitreal injection of rtPA (0.0004) ranked lower, indicating a higher likelihood of recurrence in these cases.

### 3.5 Assessment of network validity and publication bias

The validity of the results was evaluated through the net heat plot, which allows us to know the designs that favour the inconsistency of the network. These data can be found in the Figures S11–S15. The comparison‐adjusted funnel plot showed that there was no publication bias in the designs of the different interventions for BCVA (*p* = 0.1516), resolution of SMH (*p* = 0.7504), VH (*p* = 0.8796) and recurrent SMH (*p* = 0.3284). However, the RD results did show publication bias between the designs of the different interventions (*p* = 0.0197). All funnel plots are available in Figures S16–S20.

## 4 DISCUSSION

Effective management of SMH up to date remains controversial; multiple studies have been performed with several levels of complexity using isolated or mixed forms of treatment. Even so, few prospective trials exist compared to retrospective ones (Confalonieri et al., 2024; Hattenbach et al., 2020). The present systematic review and network‐meta‐analysis findings offer a clearer comprehension of the treatment efficacy, suggesting that conservative methods have better outcomes. However, great heterogeneity is present between studies, in addition to inconsistency between direct and indirect estimates across several outcomes. Nevertheless, interpretation should be cautious due to the network's structural limitations and lack of multi‐arm or looping studies. Future research including direct comparisons among all key interventions within the same trial would strengthen the confidence in these findings.

While prior meta‐analysis reported variable evidence regarding the outcomes presented here, this is the first comprehensive synthesis that includes conservative, medical and surgical approaches for SMH management. Across both the overall analysis and the n‐AMD‐specific sub‐analysis, the findings demonstrated a preference for non‐vitrectomy approaches over vitrectomy‐based interventions in terms of BCVA outcomes. These results revealed low to moderate overall heterogeneity (*I* ^2^: 28.29%) between studies, with minimal heterogeneity within treatment designs and a non‐significant trend towards network inconsistency, possibly due to varied study methodologies, population characteristics, sample sizes and potential biases. In our analysis, the ‘Observation’ group comprised only two studies, totalling 26 eyes. Notably, one of these studies included patients with retinal macroaneurysms, while the other permitted salvage vitrectomy in 38% of cases. These factors undermine the classification of this group as representing true ‘pure observation’. Consequently, the findings related to the ‘Observation’ group should be interpreted with caution due to the limited sample size and marked clinical heterogeneity. Moreover, the studies that included an observation arm did not report statistically significant differences in outcomes when compared to other treatment strategies (Mun et al., 2022). We presented a ranking of treatments as well, based on direct and indirect results regarding SMH resolution favouring invasive therapies, and evaluating the risk of complications. To the best of our knowledge, this network meta‐analysis is the first of its kind in the management of SMH. It highlights a mean path length greater than two across outcomes, indicating increased uncertainty in effect estimates—likely due to variations in study populations, methodologies and treatment effects across indirect comparisons. Future research involving direct head‐to‐head comparisons of key SMH treatments could enhance the reliability of these findings and reduce dependence on extended indirect evidence.

In assessing SMH resolution between treatments, we found a clear positive effect towards mixed interventions against anti‐VEGF alone, with high heterogeneity between studies and a great network inconsistency between the different study designs. Specifically, the combination of vitrectomy, rtPA and anti‐VEGF was statistically superior. These findings are strongly aligned with the results reported by Veritti et al. (2024), who observed significant improvements in BCVA at both 1‐month and 6‐month follow‐ups following the administration of combined tPA and anti‐VEGF therapy. Similarly, He and collaborators (2023) found a significant improvement in BCVA at 1, 3 and 6 months, with a statistically significant reduction in foveal thickness with the combination therapy of anti‐VEGF and rtPA. Conversely, Shaheen et al. (2024) compared anti‐VEGFs against a surgical approach, finding no significant difference between groups; additionally, their surgical studies showed a low level of certainty of evidence and low reliability of evidence. All these studies described reported persistent moderate to high heterogeneity between trials.

Adverse events derived from SMH management are reported at different rates of frequency; the commonest reported are RD, VH and the recurrence of the SMH (Boral et al., 2023; Confalonieri et al., 2024; Szeto et al., 2024). While others have been described as hyphema (Boral et al., 2023; Szeto et al., 2024; Thompson & Sjaarda, 2005), rise in intraocular pressure (Kishikova et al., 2021), retinal tears/breaks (Lee et al., 2021; Shin et al., 2015; Tranos et al., 2021), macular holes (Rickmann et al., 2021), cataracts (Kishikova et al., 2021; Sniatecki et al., 2021; Thompson & Sjaarda, 2005), macular neovascularization (Caporossi et al., 2022), vitreous opacity (Fujikawa et al., 2013) and hypotonia (Boral et al., 2023). When assessing complications, RD risk appears to be lower with anti‐VEGF monotherapy or when anti‐VEGF is combined with PD or PPV and subretinal injection of rtPA and PD. Furthermore, complications such as VH were more commonly associated with isolated vitrectomy, and especially with complex PPV‐based combinations, including the use of rtPA and AM implantation. In terms of SMH recurrence, PD‐based therapies, especially when combined with rtPA and/or anti‐VEGF, showed a lower risk of submacular rebleeding. Other studies reporting on SMH complication risks, such as Shaheen et al. (2024), similarly found significant inconsistencies and thus focused on overall incidence rates, indicating lower rates of RD, cataracts and proliferative vitreoretinopathy with anti‐VEGF compared to surgical approaches. He et al. (2023) observed complication rates ranging from 2.4% to 20% when comparing tPA combined with anti‐VEGF to other treatments. Giansanti et al. emphasised the higher incidence of RD and cataracts requiring surgery with submacular procedures. Confalonieri et al. (2024) documented complication rates but did not analyse incidence or risk systematically.

Our study has some limitations. First, the high heterogeneity of the included articles should be interpreted with caution. Inconsistency in the representation of some data could affect the validity and interpretation of the results of some studies. These may have incurred a reporting bias. In addition, this heterogeneity has also been detected by other authors before (Veritti et al., 2024). On the other hand, most of the studies had a retrospective methodological design. This could have led to limitations in data collection and analysis. Another limitation encountered is the surgeon's preference in the choice of procedure due to the lack of consensus to date. In addition, the low incidence of the disease makes it difficult to develop randomised clinical trials with large sample sizes. Regarding the methodological limitations of this study, we declare that we were unable to contact the authors of the included papers to obtain additional data of interest that could not be taken into account. Furthermore, we also did not explore the articles published in the grey literature. The search equation may not have been sufficiently sensitive and specific. This limitation was demonstrated by the fact that the pooled search showed several studies that had not been considered in the initial literature search. Finally, data on RAM‐ and trauma‐related SMH were clearly limited in both quantity and clinical consistency, precluding robust subgroup analyses. As for PCV, although moderately represented in some treatment arms, the total number of studies exclusively assessing this aetiology did not allow for a connected and analysable PCV‐specific network. Therefore, only n‐AMD was selected for a specific network analysis.

The strengths of this review include the need to develop consensus guidelines for the therapeutic approach to SMH. To enhance the interpretability of the findings, we conducted a subanalysis based on aetiology, specifically focusing on n‐AMD, in an effort to homogenise the study populations and reduce clinical heterogeneity. In addition, consideration of the patient's baseline VA and the size of the SMH are potential confounders that should be taken into account in the design of future studies. This stratified approach aims to provide more tailored insights for clinical decision‐making in n‐AMD‐related SMH. Furthermore, the complications associated with each therapeutic approach should be further studied to determine the risk–benefit of each intervention.

There is currently no consensus regarding evidence‐based standard treatment for SMH, although there is a trend towards minimally invasive approaches. While some surgical interventions demonstrate potential benefits, the lack of consensus and standardised protocols remains a challenge. Regardless of the choice of the primary treatment approach, factors such as the time to treatment, size of the lesion, and accompanying intravitreal treatment with VEGF inhibitors seem to be decisive for the functional outcome. Further large‐scale, randomised controlled trials are essential to establish optimal treatment guidelines and improve patient outcomes.

## CONFLICT OF INTEREST STATEMENT

The authors have declared no conflicts of interest.

## Supporting information


Data S1.
