# Supplementary material for: Interventions for submacular haemorrhage: A systematic review and network meta‐analysis of controversies—On behalf of the Spanish Vitreo‐Retinal Society (SERV)
Source: Acta Ophthalmol. 2025 Aug 7;104(2):139–63. doi: 10.1111/aos.17570 (PMC12888954; doi:10.1111/aos.17570)
Supplement: Supplementary file 1 — Data S1. [file AOS-104-139-s001.docx]

**Supplementary Material**

**Interventions for Submacular Hemorrhage: A Systematic Review and Network Meta-Analysis of Controversies – On Behalf of the Spanish Vitreo-Retinal Society (SERV)**

**eFigure 1.** Proportion of direct and indirect evidence for each network estimate adjusted by the random effects model in the BCVA assessment. (p. 5)

**eFigure 2.** Proportion of direct and indirect evidence for each network estimate adjusted by the random effects model in the SMH resolution assessment. (p. 7)

**eFigure 3.** Proportion of direct and indirect evidence for each network estimate adjusted by the random effects model in the retinal detachment assessment. (p. 8)

**eFigure 4.** Proportion of direct and indirect evidence for each network estimate adjusted by the random effects model in the vitreous hemorraghe assessment. (p. 9)

**eFigure 5.** Proportion of direct and indirect evidence for each network estimate adjusted by the random effects model in the recurrent SMH assessment. (p. 10)

**eFigure 6.** Forest plot comparing the direct and indirect evidence for the different comparisons between the therapies evaluated in BCVA. (p. 11)

**eFigure 7.** Forest plot comparing the direct and indirect evidence for the different comparisons between the therapies evaluated in SMH resolution. (p. 13)

**eFigure 8.** Forest plot comparing the direct and indirect evidence for the different comparisons between the therapies evaluated in terms of retinal detachment. (p. 14)

**eFigure 9.** Forest plot comparing the direct and indirect evidence for the different comparisons between the therapies evaluated in terms of vitreous hemorraghe. (p. 16)

**eFigure 10.** Forest plot comparing the direct and indirect evidence for the different comparisons between the therapies evaluated in terms of recurrent SMH. (p. 17)

**eFigure 11.** Heat plot of the network assessing the inconsistency of the designs that make up the network for BCVA evaluation. (p. 18)

**eFigure 12.** Heat plot of the network assessing the inconsistency of the designs that make up the network for SMH resolution evaluation. (p. 19)

**eFigure 13.** Heat plot of the network assessing the inconsistency of the designs that make up the network for retinal detachment evaluation. (p. 20)

**eFigure 14.** Heat plot of the network assessing the inconsistency of the designs that make up the network for vitreous hemorraghe evaluation. (p. 21)

**eFigure 15.** Heat plot of the network assessing the inconsistency of the designs that make up the network for recurrent SMH evaluation. (p. 22)

**eFigure 16.** Comparison-adjusted funnel plot to assess the risk of publication bias in BCVA analysis. (p. 23)

**eFigure 17.** Comparison-adjusted funnel plot to assess the risk of publication bias in SMH resolution analysis. (p. 24)

**eFigure 18.** Comparison-adjusted funnel plot to assess the risk of publication bias in retinal detachment analysis. (p. 25)

**eFigure 19.** Comparison-adjusted funnel plot to assess the risk of publication bias in vitreous hemorraghe analysis. (p. 26)

**eFigure 20.** Comparison-adjusted funnel plot to assess the risk of publication bias in recurrent SMH analysis. (p. 27)

**eTable 1.** Summary of the main characteristics and GRADE evaluation of the included studies. (p. 28)

**eTable 2.** Direct and indirect estimates comparing the efficacy of different treatments in BCVA for SMH. (p. 33)

**eTable 3.** Direct and indirect estimates comparing the efficacy of different treatments in SMH resolution. (p. 35)

**eTable 4.** Direct and indirect estimates comparing the security of different treatments in terms of retinal detachment. (p. 36)

**eTable 5.** Direct and indirect estimates comparing the security of different treatments in terms of vitreous hemorraghe. (p. 39)

**eTable 6.** Direct and indirect estimates comparing the security of different treatments in terms of recurrent SMH. (p. 41)


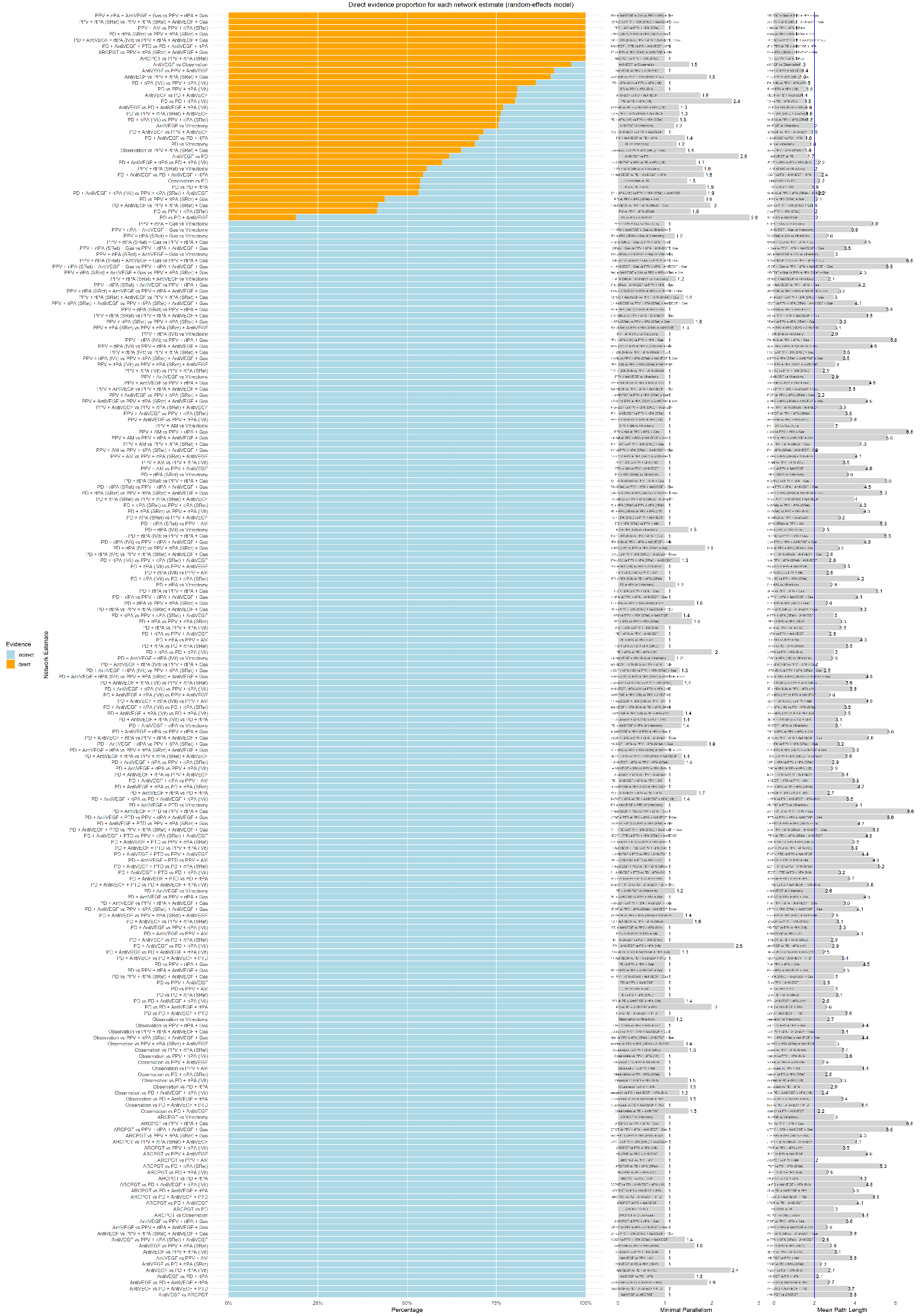
**eFigure 1.** Proportion of direct and indirect evidence for each network estimate adjusted by the random effects model in the BCVA assessment. AM: amniotic membrane; AntiVEGF: Anti-vascular endothelial growth factor; ARCPGT: autologous retinal pigmentary retinal pigment epithelium-choroid patch graft transplantations; PD: pneumatic displacement; PPV: pars plana vitrectomy; PTD: Photodynamic Therapy; rtPA: recombinant tissue plasminogen activator; SRet: subretinal; IVit: intravitreal injection.

(Continue)


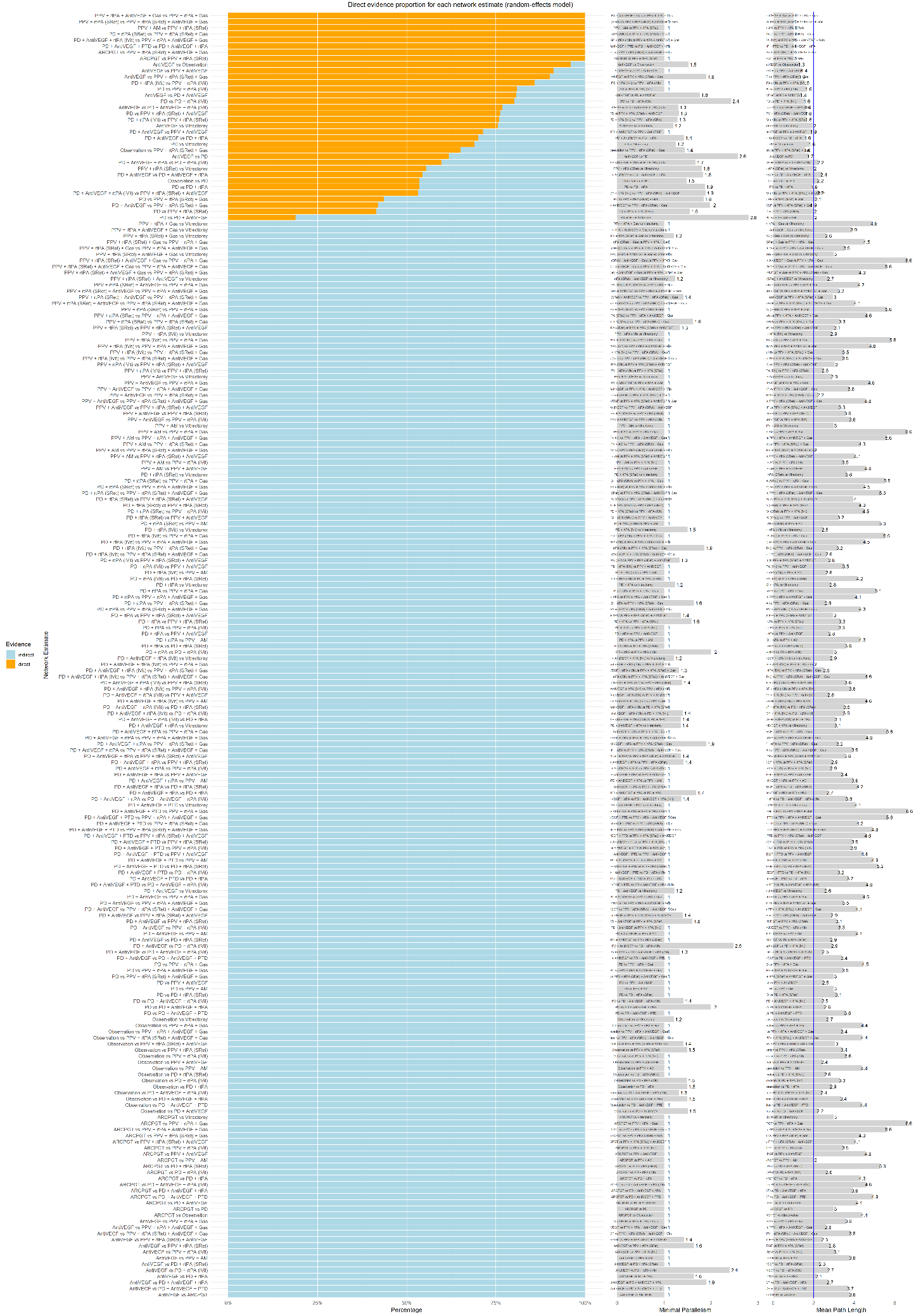


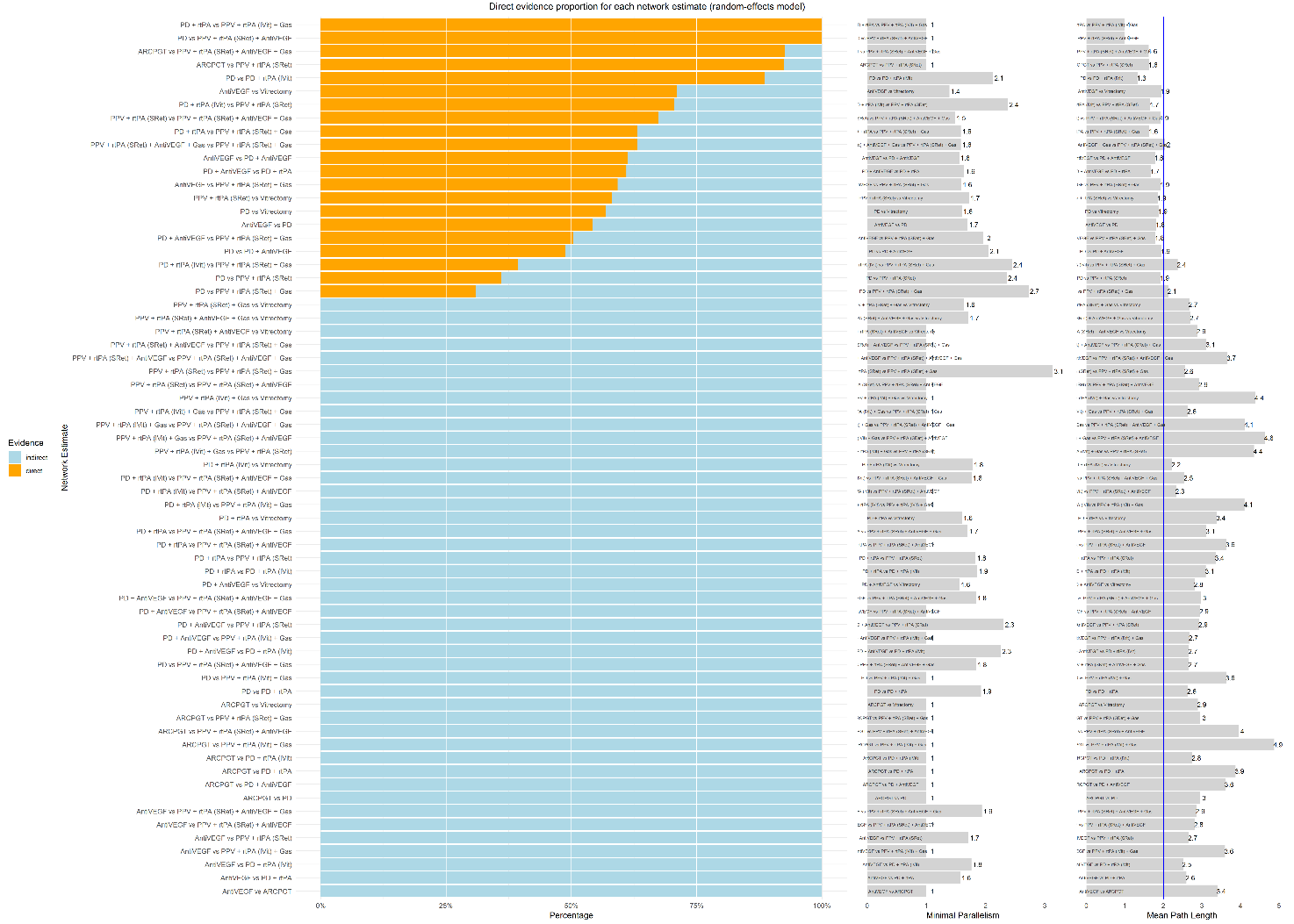
**eFigure 2.** Proportion of direct and indirect evidence for each network estimate adjusted by the random effects model in the SMH resolution assessment. AM: amniotic membrane; AntiVEGF: Anti-vascular endothelial growth factor; ARCPGT: autologous retinal pigmentary retinal pigment epithelium-choroid patch graft transplantations; PD: pneumatic displacement; PPV: pars plana vitrectomy; rtPA: recombinant tissue plasminogen activator; SRet: subretinal; IVit: intravitreal injection.


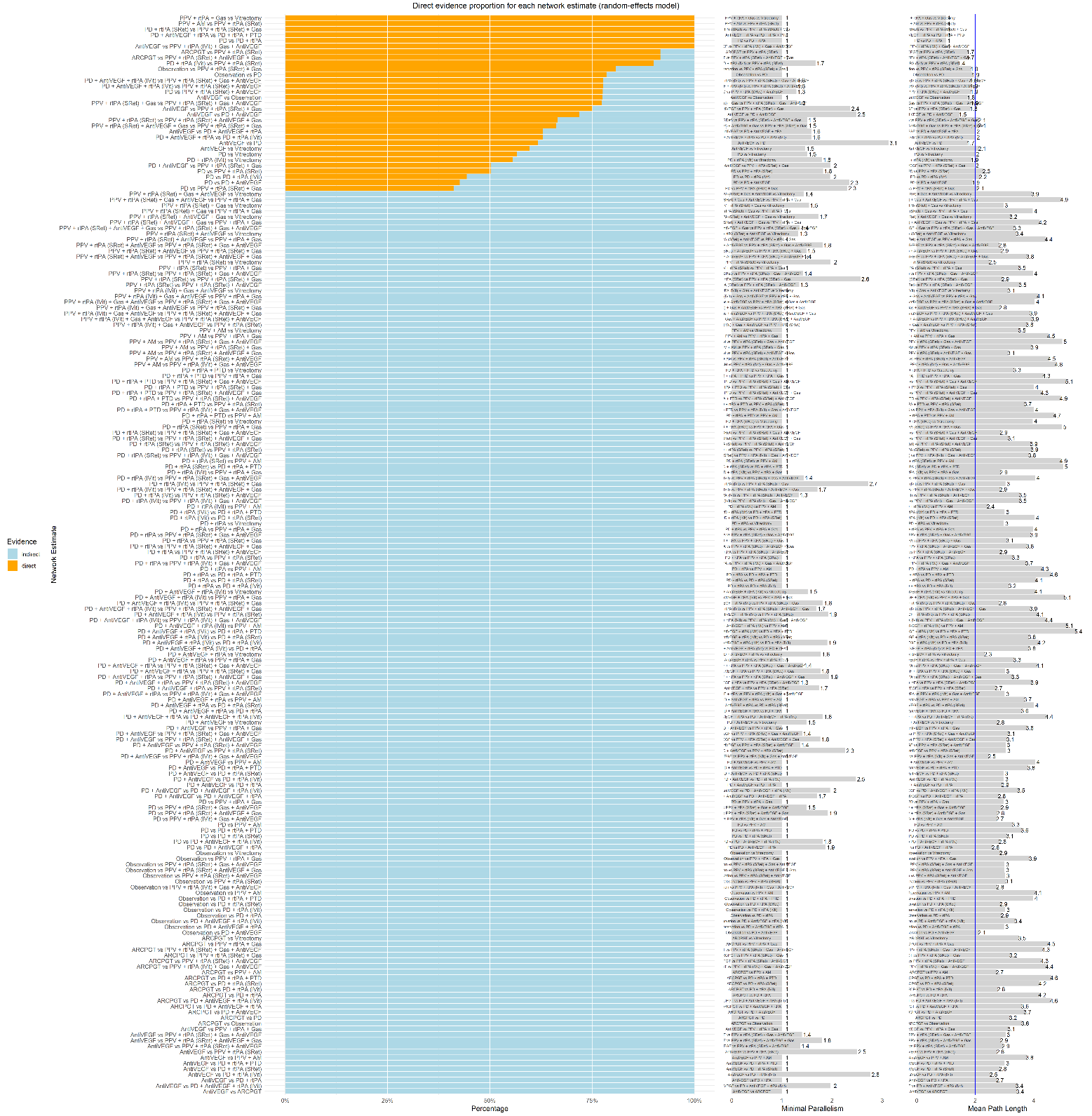
**eFigure 3.** Proportion of direct and indirect evidence for each network estimate adjusted by the random effects model in the retinal detachment assessment. AntiVEGF: Anti-vascular endothelial growth factor; ARCPGT: autologous retinal pigmentary retinal pigment epithelium-choroid patch graft transplantations; PD: pneumatic displacement; PPV: pars plana vitrectomy; rtPA: recombinant tissue plasminogen activator; SRet: subretinal; IVit: intravitreal injection.


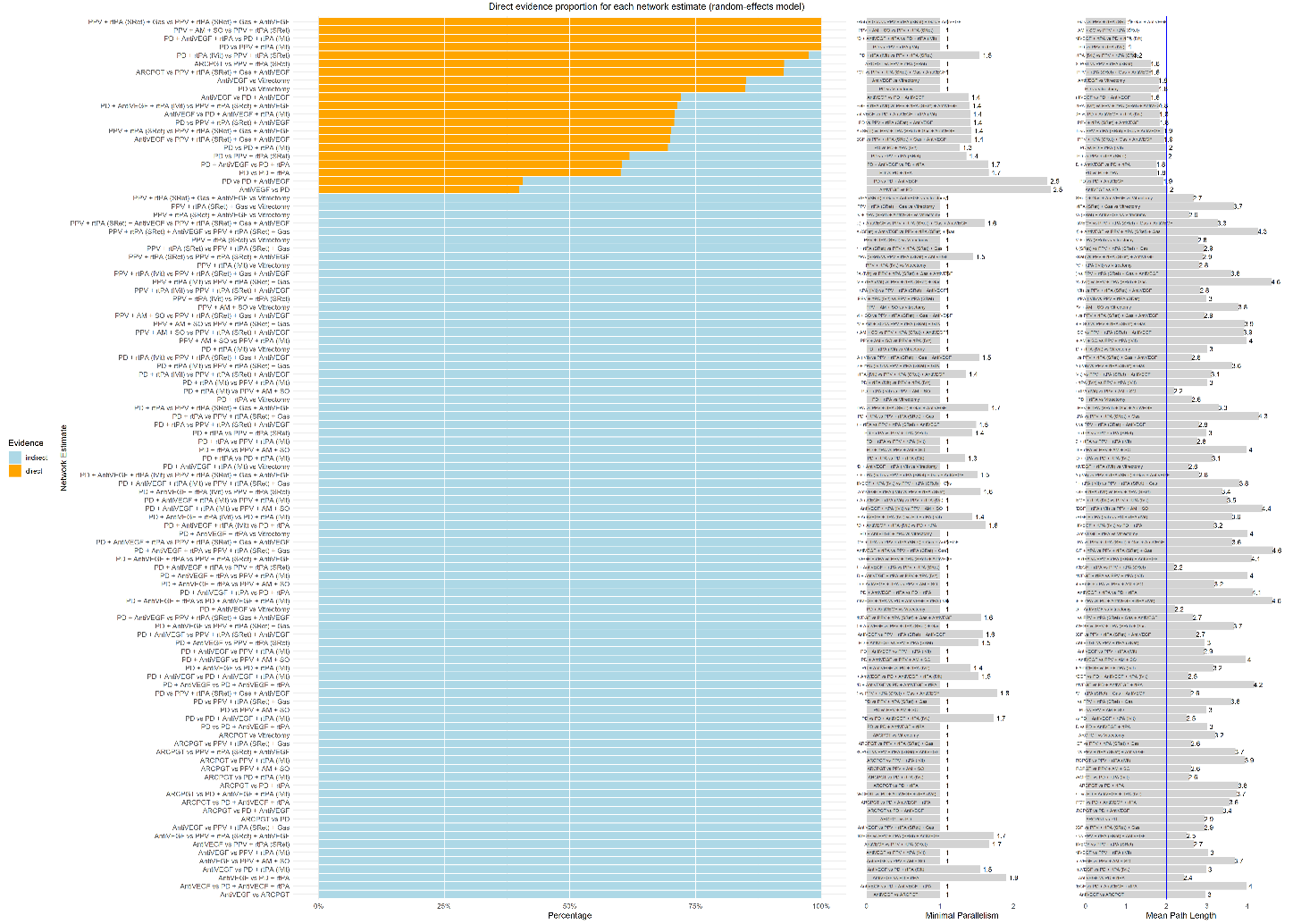
**eFigure 4.** Proportion of direct and indirect evidence for each network estimate adjusted by the random effects model in the vitreous hemorraghe assessment. AM: amniotic membrane; AntiVEGF: Anti-vascular endothelial growth factor; ARCPGT: autologous retinal pigmentary retinal pigment epithelium-choroid patch graft transplantations; PD: pneumatic displacement; PPV: pars plana vitrectomy; rtPA: recombinant tissue plasminogen activator; SRet: subretinal; IVit: intravitreal injection.


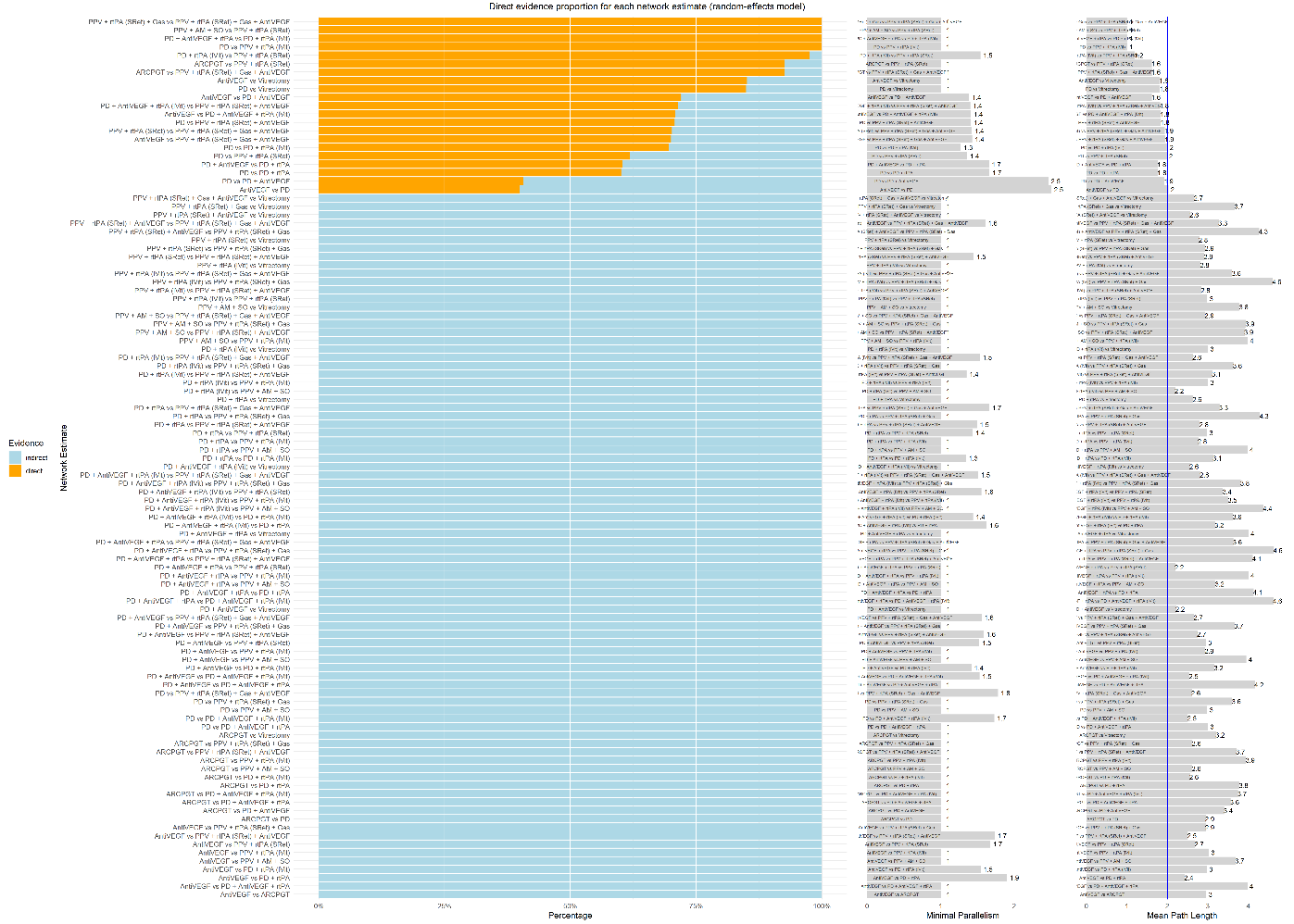
**eFigure 5.** Proportion of direct and indirect evidence for each network estimate adjusted by the random effects model in the recurrent SMH assessment. AntiVEGF: Anti-vascular endothelial growth factor; ARCPGT: autologous retinal pigmentary retinal pigment epithelium-choroid patch graft transplantations; PD: pneumatic displacement; PPV: pars plana vitrectomy; rtPA: recombinant tissue plasminogen activator; SRet: subretinal; IVit: intravitreal injection.


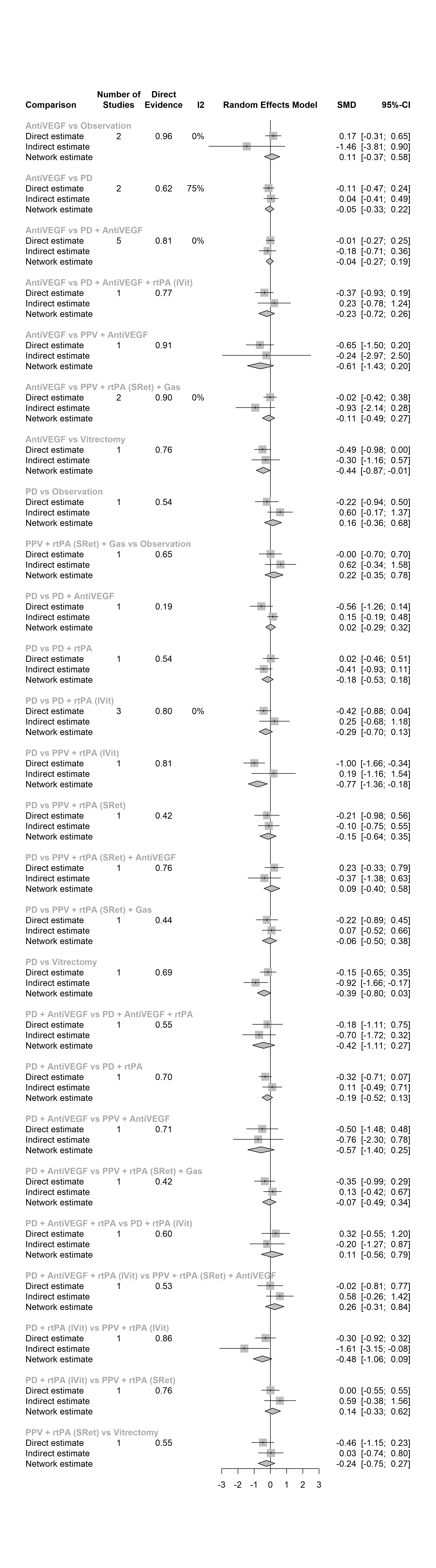
**eFigure 6.** Forest plot comparing the direct and indirect evidence for the different comparisons between the therapies evaluated in BCVA. AM: amniotic membrane; AntiVEGF: Anti-vascular endothelial growth factor; ARCPGT: autologous retinal pigmentary retinal pigment epithelium-choroid patch graft transplantations; PD: pneumatic displacement; PPV: pars plana vitrectomy; PTD: Photodynamic Therapy; rtPA: recombinant tissue plasminogen activator; SRet: subretinal; IVit: intravitreal injection.


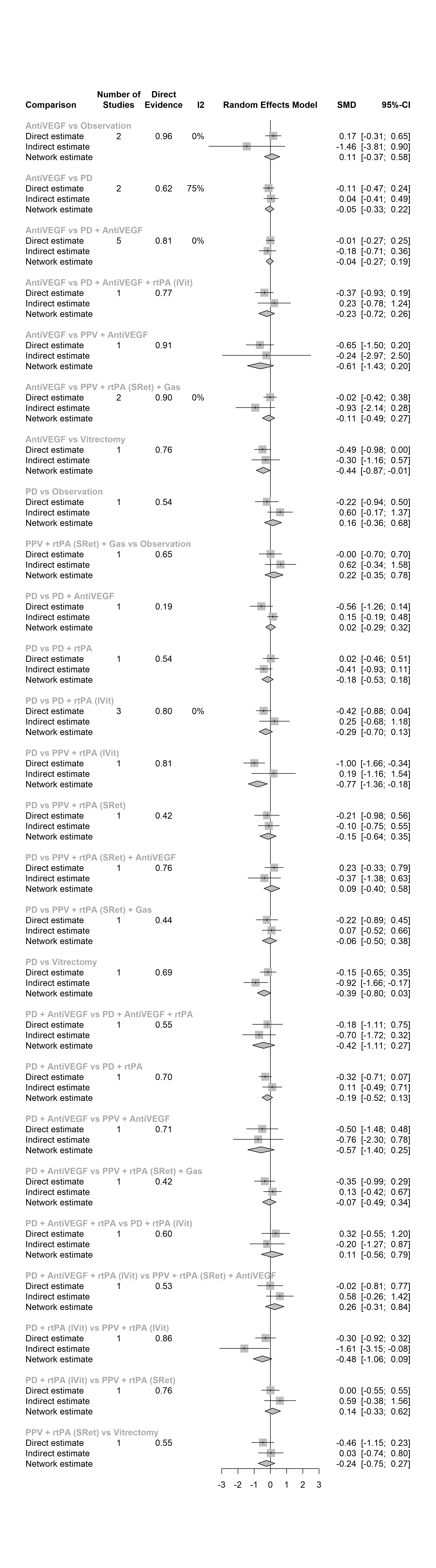
(Coninue)


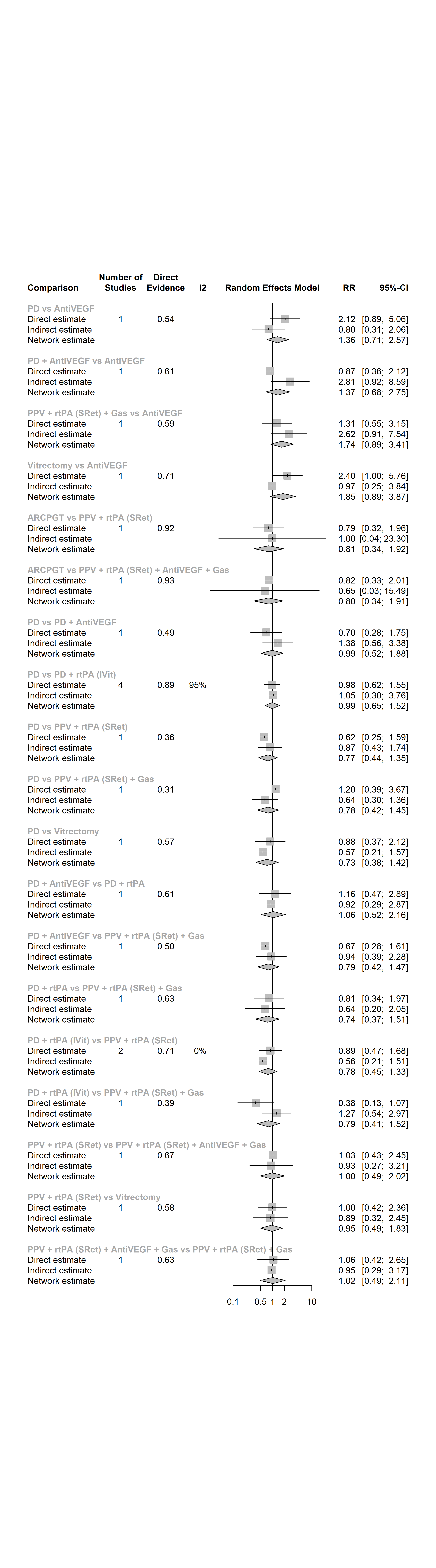
**eFigure 7.** Forest plot comparing the direct and indirect evidence for the different comparisons between the therapies evaluated in SMH resolution. AntiVEGF: Anti-vascular endothelial growth factor; ARCPGT: autologous retinal pigmentary retinal pigment epithelium-choroid patch graft transplantations; PD: pneumatic displacement; PPV: pars plana vitrectomy; rtPA: recombinant tissue plasminogen activator; SRet: subretinal; IVit: intravitreal injection.

**eFigure 8.** Forest plot comparing the direct and indirect evidence for the different comparisons between the therapies evaluated in terms of retinal detachment. AntiVEGF: Anti-vascular endothelial growth factor; ARCPGT: autologous retinal pigmentary retinal pigment epithelium-choroid patch graft transplantations; PD: pneumatic displacement; PPV: pars plana vitrectomy; rtPA: recombinant tissue plasminogen activator; SRet: subretinal; IVit: intravitreal injection.


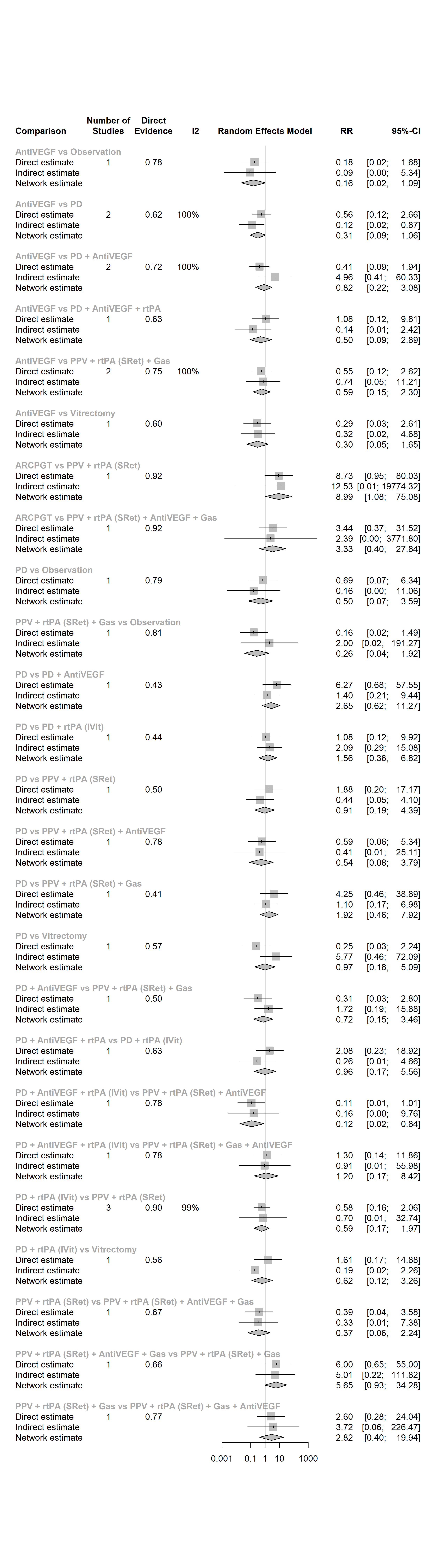


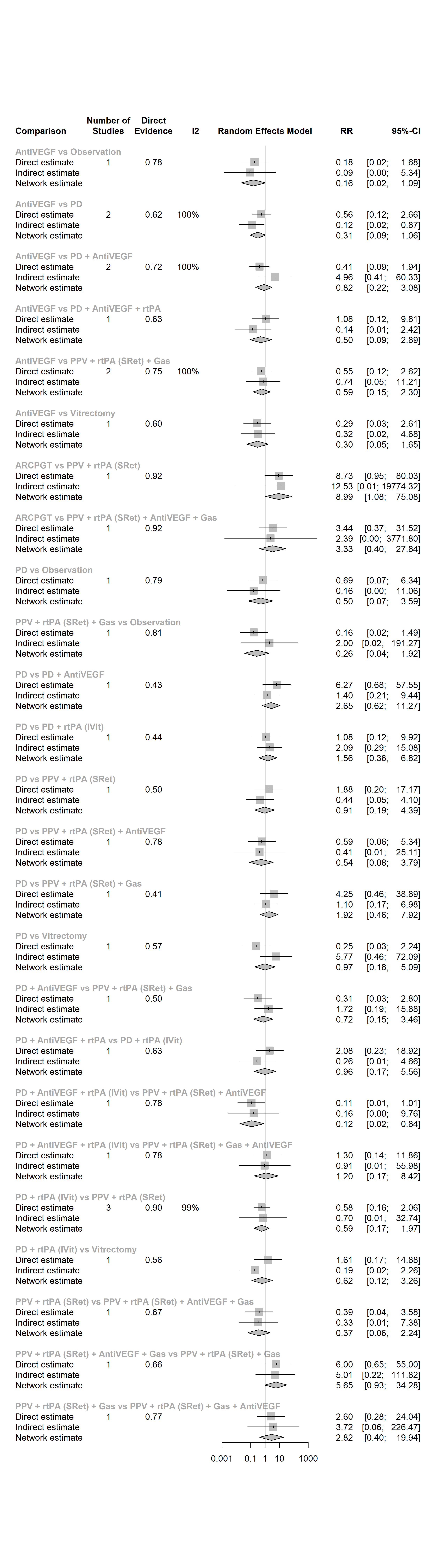
(Continue)


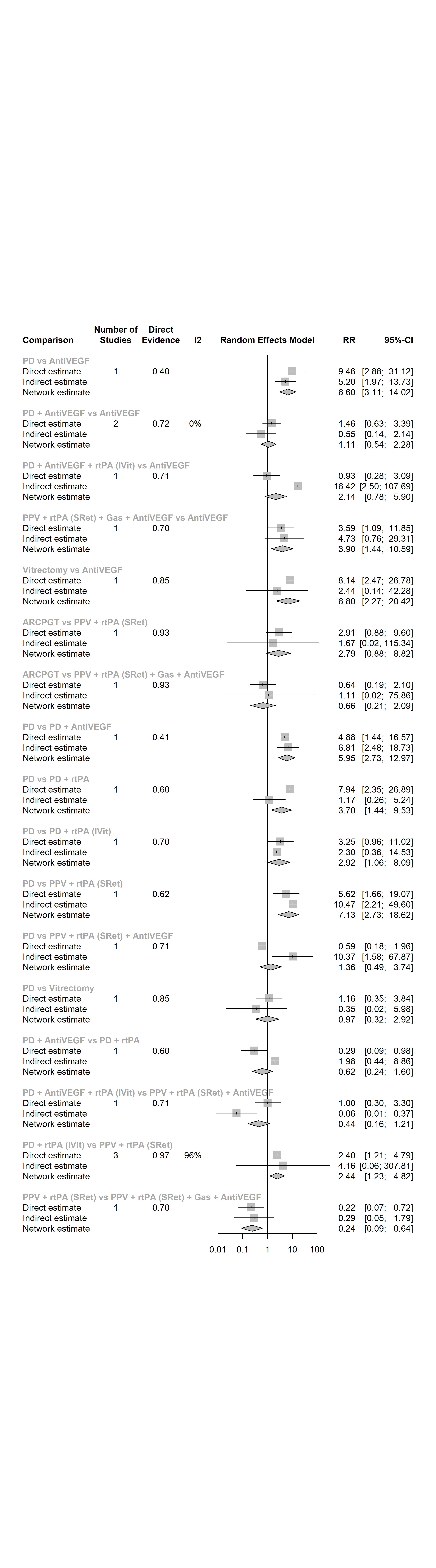
**eFigure 9.** Forest plot comparing the direct and indirect evidence for the different comparisons between the therapies evaluated in terms of vitreous hemorraghe. AntiVEGF: Anti-vascular endothelial growth factor; ARCPGT: autologous retinal pigmentary retinal pigment epithelium-choroid patch graft transplantations; PD: pneumatic displacement; PPV: pars plana vitrectomy; rtPA: recombinant tissue plasminogen activator; SRet: subretinal; IVit: intravitreal injection.

**eFigure 10.** Forest plot comparing the direct and indirect evidence for the different comparisons between the therapies evaluated in terms of recurrent SMH. AntiVEGF: Anti-vascular endothelial growth factor; ARCPGT: autologous retinal pigmentary retinal pigment epithelium-choroid patch graft transplantations; PD: pneumatic displacement; PPV: pars plana vitrectomy; rtPA: recombinant tissue plasminogen activator; SRet: subretinal; IVit: intravitreal injection.


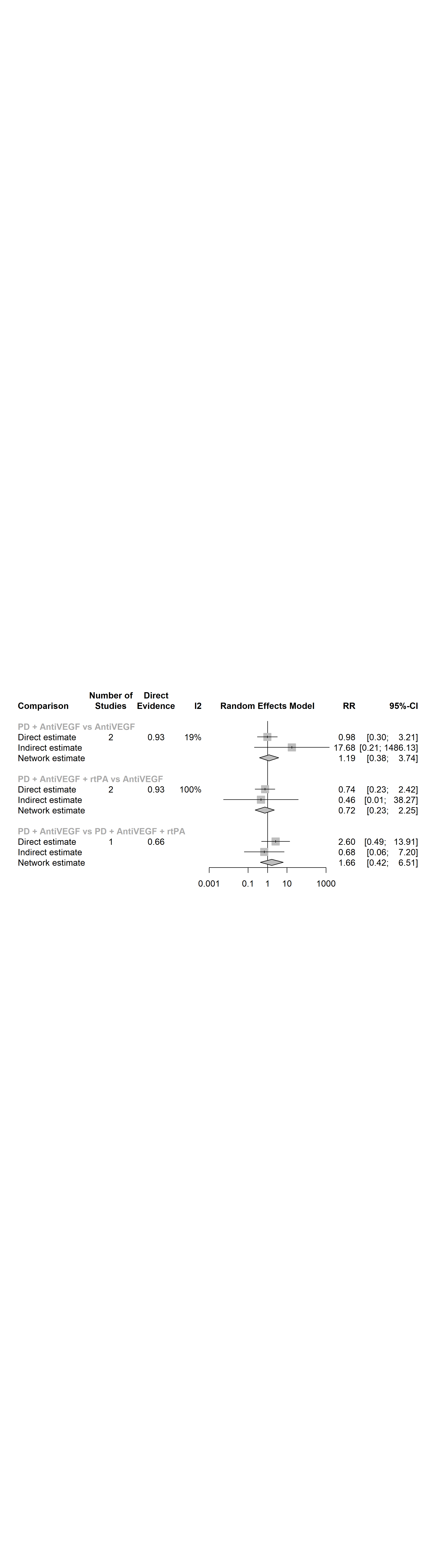


**eFigure 11.** Heat plot of the network assessing the inconsistency of the designs that make up the network for BCVA evaluation. The size of the square indicates the significance of the comparison based on the direct evidence available. The colour gradation shows the intensity of the inconsistency (warm colours indicate higher inconsistency and cool colours indicate lower inconsistency). AM: amniotic membrane; AntiVEGF: Anti-vascular endothelial growth factor; ARCPGT: autologous retinal pigmentary retinal pigment epithelium-choroid patch graft transplantations; PD: pneumatic displacement; PPV: pars plana vitrectomy; PTD: Photodynamic Therapy; rtPA: recombinant tissue plasminogen activator; SRet: subretinal; IVit: intravitreal injection.





**eFigure 12.** Heat plot of the network assessing the inconsistency of the designs that make up the network for SMH resolution evaluation. The size of the square indicates the significance of the comparison based on the direct evidence available. The colour gradation shows the intensity of the inconsistency (warm colours indicate higher inconsistency and cool colours indicate lower inconsistency). AntiVEGF: Anti-vascular endothelial growth factor; ARCPGT: autologous retinal pigmentary retinal pigment epithelium-choroid patch graft transplantations; PD: pneumatic displacement; PPV: pars plana vitrectomy; rtPA: recombinant tissue plasminogen activator; SRet: subretinal; IVit: intravitreal injection.





**eFigure 13.** Heat plot of the network assessing the inconsistency of the designs that make up the network for retinal detachment evaluation. The size of the square indicates the significance of the comparison based on the direct evidence available. The colour gradation shows the intensity of the inconsistency (warm colours indicate higher inconsistency and cool colours indicate lower inconsistency). AntiVEGF: Anti-vascular endothelial growth factor; ARCPGT: autologous retinal pigmentary retinal pigment epithelium-choroid patch graft transplantations; PD: pneumatic displacement; PPV: pars plana vitrectomy; rtPA: recombinant tissue plasminogen activator; SRet: subretinal; IVit: intravitreal injection.



**eFigure 14.** Heat plot of the network assessing the inconsistency of the designs that make up the network for vitreous hemorraghe evaluation. The size of the square indicates the significance of the comparison based on the direct evidence available. The colour gradation shows the intensity of the inconsistency (warm colours indicate higher inconsistency and cool colours indicate lower inconsistency). AntiVEGF: Anti-vascular endothelial growth factor; ARCPGT: autologous retinal pigmentary retinal pigment epithelium-choroid patch graft transplantations; PD: pneumatic displacement; PPV: pars plana vitrectomy; rtPA: recombinant tissue plasminogen activator; SRet: subretinal; IVit: intravitreal injection.


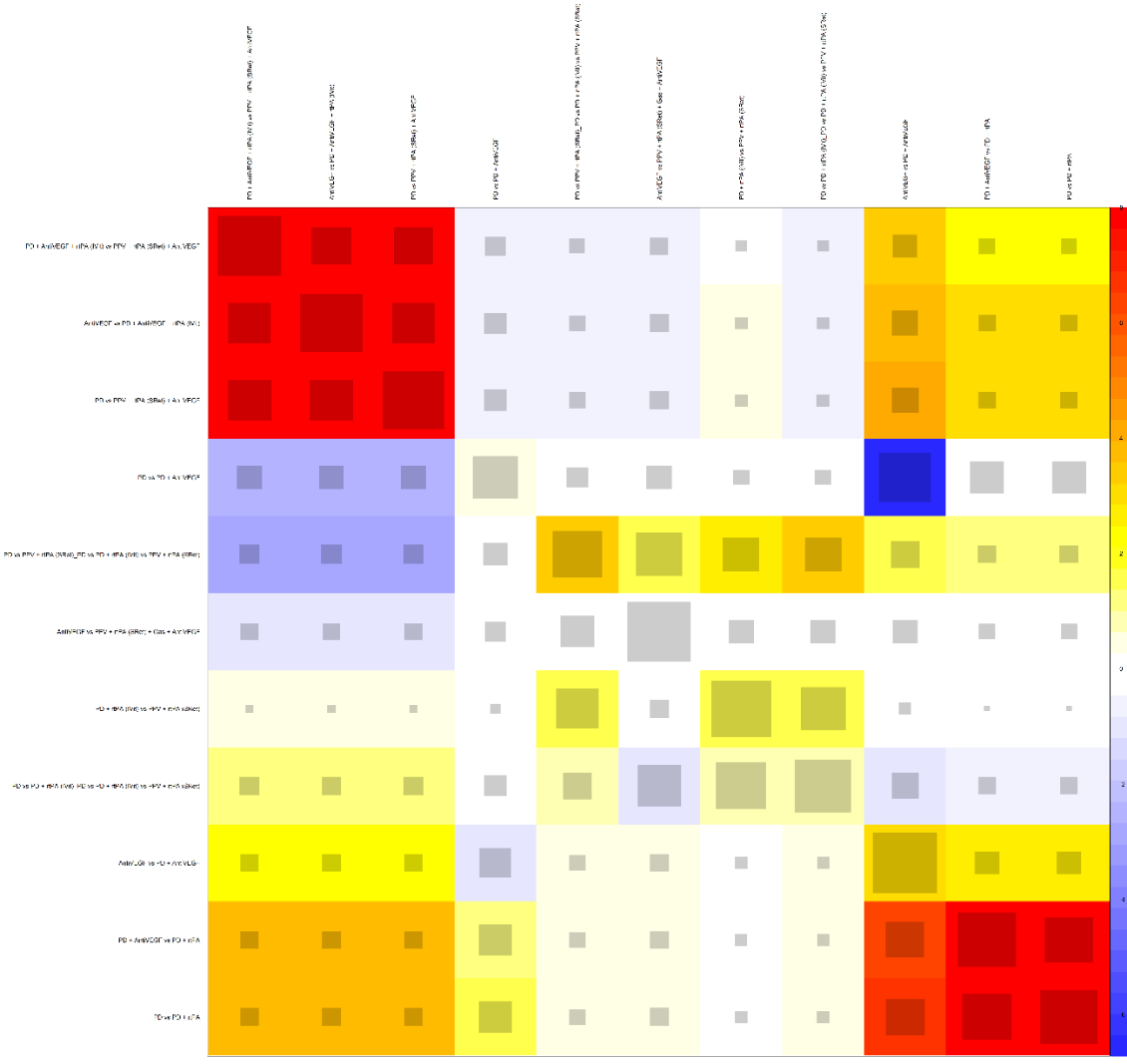


**eFigure 15.** Heat plot of the network assessing the inconsistency of the designs that make up the network for recurrent SMH evaluation. The size of the square indicates the significance of the comparison based on the direct evidence available. The colour gradation shows the intensity of the inconsistency (warm colours indicate higher inconsistency and cool colours indicate lower inconsistency). AntiVEGF: Anti-vascular endothelial growth factor; ARCPGT: autologous retinal pigmentary retinal pigment epithelium-choroid patch graft transplantations; PD: pneumatic displacement; PPV: pars plana vitrectomy; rtPA: recombinant tissue plasminogen activator; SRet: subretinal; IVit: intravitreal injection.

**

**


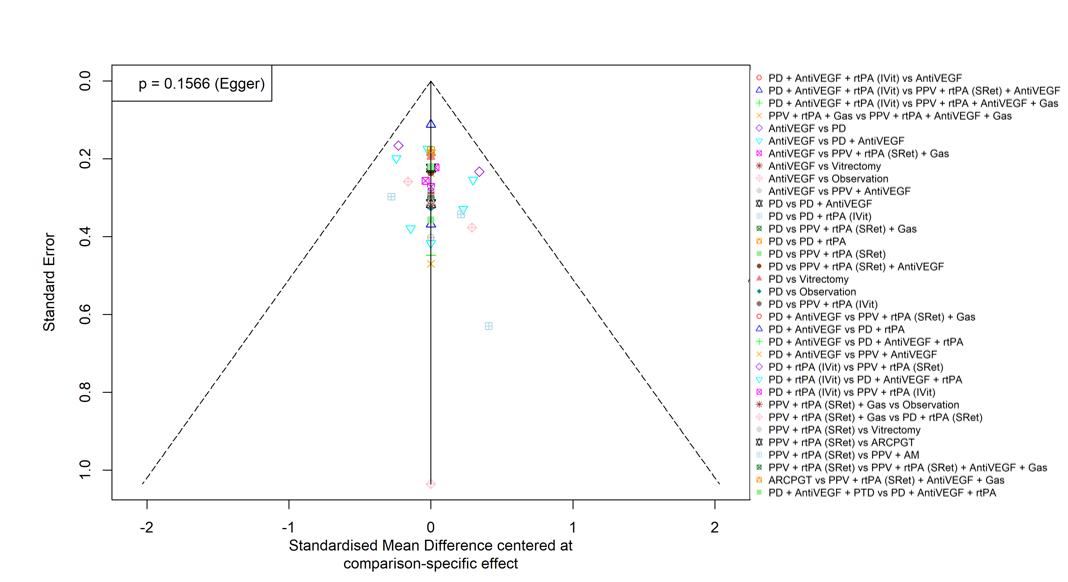
**eFigure 16.** Comparison-adjusted funnel plot to assess the risk of publication bias in BCVA analysis. AM: amniotic membrane; AntiVEGF: Anti-vascular endothelial growth factor; ARCPGT: autologous retinal pigmentary retinal pigment epithelium-choroid patch graft transplantations; PD: pneumatic displacement; PPV: pars plana vitrectomy; PTD: Photodynamic Therapy; rtPA: recombinant tissue plasminogen activator; SRet: subretinal; IVit: intravitreal injection.


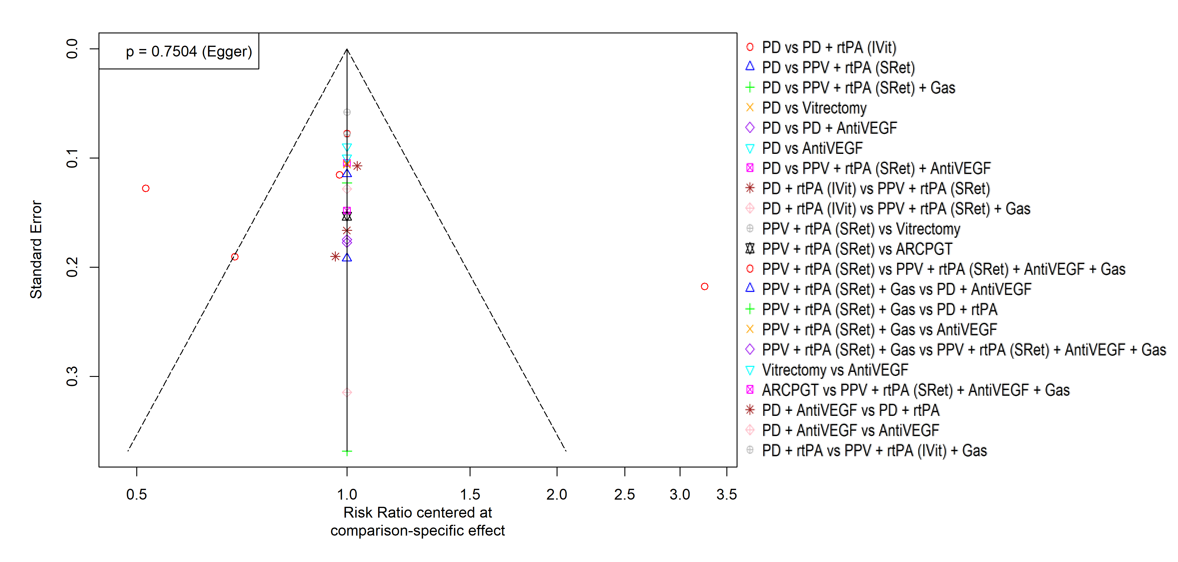
**eFigure 17.** Comparison-adjusted funnel plot to assess the risk of publication bias in SMH resolution analysis. AntiVEGF: Anti-vascular endothelial growth factor; ARCPGT: autologous retinal pigmentary retinal pigment epithelium-choroid patch graft transplantations; PD: pneumatic displacement; PPV: pars plana vitrectomy; rtPA: recombinant tissue plasminogen activator; SRet: subretinal; IVit: intravitreal injection.

**eFigure 18.** Comparison-adjusted funnel plot to assess the risk of publication bias in retinal detachment analysis. AntiVEGF: Anti-vascular endothelial growth factor; ARCPGT: autologous retinal pigmentary retinal pigment epithelium-choroid patch graft transplantations; PD: pneumatic displacement; PPV: pars plana vitrectomy; rtPA: recombinant tissue plasminogen activator; SRet: subretinal; IVit: intravitreal injection.


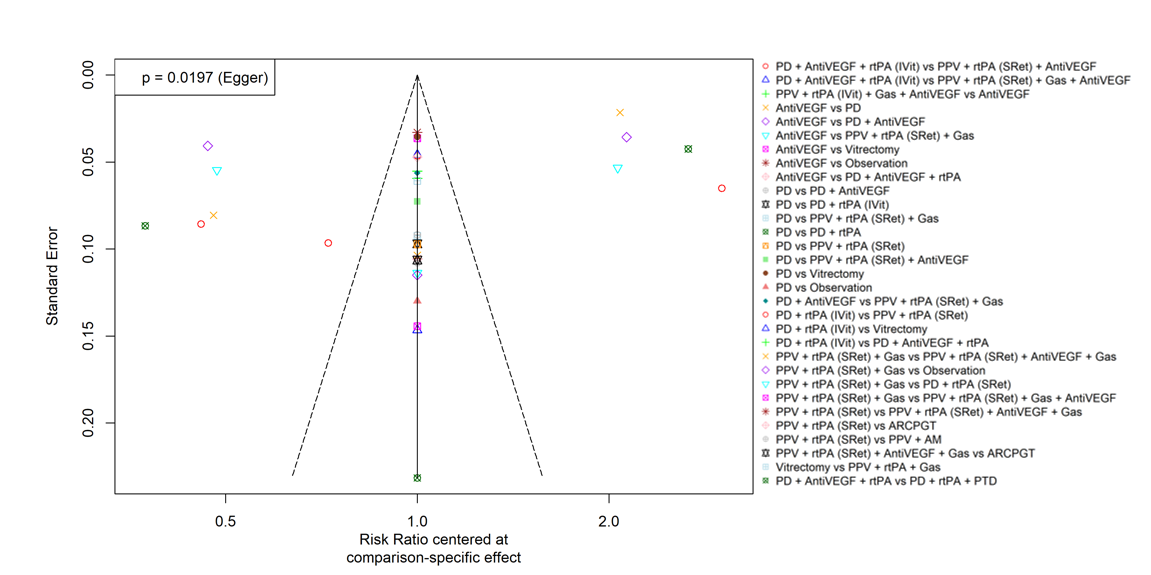


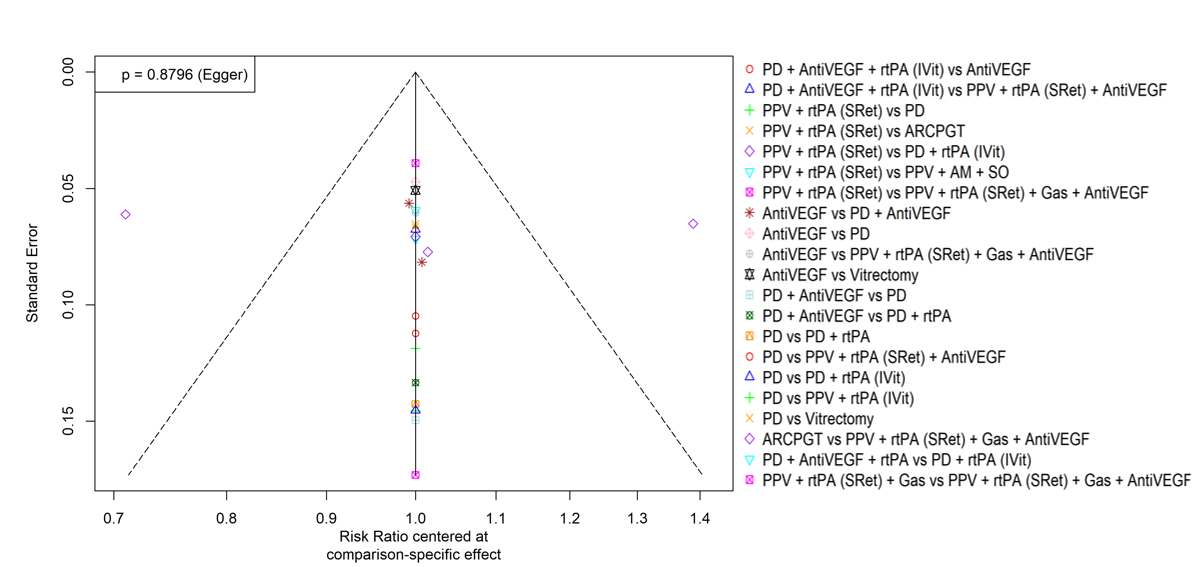
**eFigure 19.** Comparison-adjusted funnel plot to assess the risk of publication bias in vitreous hemorraghe analysis. AntiVEGF: Anti-vascular endothelial growth factor; ARCPGT: autologous retinal pigmentary retinal pigment epithelium-choroid patch graft transplantations; PD: pneumatic displacement; PPV: pars plana vitrectomy; rtPA: recombinant tissue plasminogen activator; SRet: subretinal; IVit: intravitreal injection.


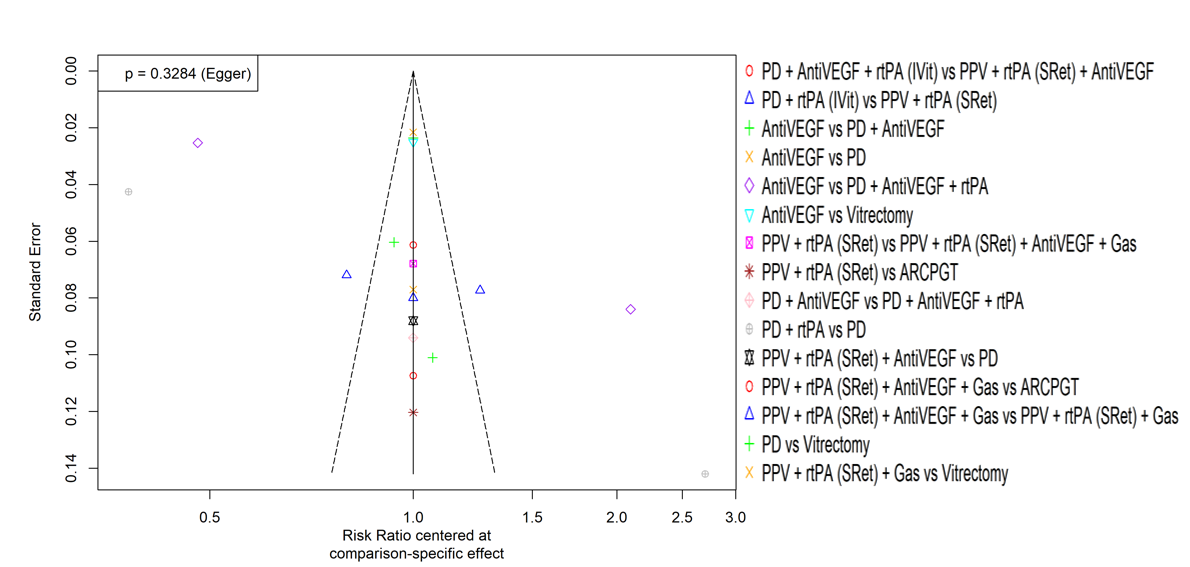
**eFigure 20.** Comparison-adjusted funnel plot to assess the risk of publication bias in recurrent SMH analysis. AntiVEGF: Anti-vascular endothelial growth factor; ARCPGT: autologous retinal pigmentary retinal pigment epithelium-choroid patch graft transplantations; PD: pneumatic displacement; PPV: pars plana vitrectomy; rtPA: recombinant tissue plasminogen activator; SRet: subretinal; IVit: intravitreal injection.

**eTable 1. Summary of GRADE evaluation of the included studies.** PROSP: prospective; Retro: restrospective; CONSEC: Consecutive series; COMPR: comparative; ITRV: interventional; NComp: non-comparative; RCT: randomized clinical trial.

| **eTable 1. Synthesis of the literature from 2004-2024** | | | |
| --- | --- | --- | --- |
| **Author, Year** | **Country** | **Study Design** | **GRADE*** |
| Hesse et al. 2000 | Germany | PROSP, CONSEC, single-center, Ncomp, ITRV, case series | Low |
| Handwerger et al. 2001 | USA | Retro, case series | Very Low |
| Haupert et al. 2001 | USA | Retro, case series | Very low |
| Hattenbach et al. 2001 | Germany | PROSP, CONSEC, single-center, Ncomp, ITRV, case series | Low |
| Schulze et al. 2002 | Germany | Retro, case series | Very low |
| Tsai et al. 2003 | Taiwan | Retro, case series | Very low |
| Olivier et al. 2010 | USA | Retro, CONSEC, COMPR, ITRV, case series | Low |
| Ratanasukon et al. 2005 | Thailand | Retro, case series | Very low |
| Wu et al. 2005 | Taiwan | PROSP, CONSEC, single-center, Ncomp, ITRV, case series | Low |
| Chan et al. 2005 | China | PROSP, CONSEC, single-center, Ncomp, ITRV, case series | Low |
| Yang et al. 2005 | China | Retro, COMPR, ITRV, case series | Low |
| Singh et al. 2006 | USA | Retro, CONSEC, Ncomp, ITRV, case series | Low |
| Mozaffarieh et al. 2006 | Austria | Retro, CONSEC, Ncomp, ITRV, case series | Very low |
| Thompson et al. 2005 | USA | Retro, CONSEC, COMPR, ITRV, case series | Low |
| Ron Y et al. 2007 | Israel | Retro, case series | VeryLow |
| Chen et al. 2007 | Australia | Retro, case series | Very low |
| Gopalakrishan et al. 2007 | India | PROSP, CONSEC, single-center, Ncomp, ITRV, case series | Low |
| Stifter et al. 2007 | Austria | Retro, case series | Very low |
| Fang et al. 2009 | Taiwan | Retro, COMPR, ITRV, case series | Low |
| Lincoff et al. 2008 | USA | Retro, Ncomp, case series | Very Low |
| Meyer et al. 2008 | Germany | Retro, CONSEC, ITRV, case series | Low |
| Muqit et al. 2008 | UK | PROSP, CONSEC, single-center, Ncomp, ITRV, case series | Low |
| Sacu et al. 2009 | Austria | Retro, COMPR, ITRV, case series | Low |
| Kamei et al. 2009 | Japan | Retro, COMPR, ITRV, case series | Low |
| Hillenkamp et al. 2010 | Germany | Retro, CONSEC, COMPR, ITRV, case series | Low |
| Treumer et al. 2010 | Germany | PROSP, CONSEC, Ncomp, ITRV, case series | Low |
| Cakir et al. 2010 | Turkey | Retro, COMPR, ITRV, case series | Low |
| McAllister et al. 2010 | Australia | PROSP, CONSEC, single-center, Ncomp, ITRV, case series | Low |
| Arias et al. 2010 | Spain | Retro, CONSEC, ITRV, case series | Low |
| Sandhu et al. 2010 | UK | PROSP, CONSEC, single-center, Ncomp, ITRV, case series | Low |
| Fine et al. 2010 | USA | Retro, case series | Low |
| Guthoff et al. 2011 | Germany | Retro, CONSEC, COMPR, ITRV, case series | Low |
| Kung et al. 2010 | Taiwan | Retro, case series | Very low |
| Tsymanava et al. 2012 | Germany | Retro, COMPR, ITRV, case series | Low |
| Mizutani et al. 2011 | Japan | Retro, case series | Very low |
| Wu et al. 2011 | China | Retro, CONSEC, COMPR, ITRV, case series | Low |
| Mayer et al. 2013 | Germany | Retro, CONSEC, COMPR, ITRV, case series | Low |
| Treumer et al. 2012 | Germany | Retro, CONSEC, Ncomp, ITRV, case series | Low |
| Hesgaard et al. 2012 | Denmark | Retro, COMPR, ITRV, case series | Low |
| Sonmez et al. 2012 | Turkey | PROSP, CONSEC, Ncomp, ITRV, case series | Low |
| Nourinia et al. 2010 | Iran | PROSP, CONSEC, single-center, Ncomp, ITRV, case series | Low |
| van Zeeburg et al. 2013 | Netherlands | Retro, case series | Very low |
| Ueda-Arakawa et al. 2012 | Japan | Retro, CONSEC, ITRV, case series | Very low |
| Rishi et al. 2012 | India | Retro, COMPR, ITRV, case series | Low |
| Papavasileiou et al. 2013 | UK | Retro, case series | Very low |
| Shienbaum et al. 2013 | USA | Retro, CONSEC, ITRV, case series | Very low |
| Fujikawa et al. 2013 | Japan | Retro, COMPR, ITRV, case series | Low |
| Cho et al. 2013 | Taiwan | Retro, case series | Very low |
| Iacono et al. 2014 | Italy | PROSP, CONSEC, single-center, Ncomp, ITRV, case series | Low |
| Jain et al. 2013 | USA | Retro, case series | Very low |
| Kim et al. 2014 | South Korea | Retro, case series | Very low |
| Chang et al. 2014 | USA | Retro, CONSEC, COMPR, ITRV, case series | Low |
| Kitahashi et al. 2014 | Japan | Retro, Ncomp, case series | Very Low |
| Sobolewska et al. 2014 | Germany | Retro, case series | Very low |
| Dewilde et al. 2014 | Belgium | Retro, case series | Very low |
| Moisseiev et al. 2014 | Israel | Retro, case series | Very low |
| Cho et al. 2015 | Korea | Retro, COMPR, ITRV, case series | Low |
| Kadonosono et al. 2015 | Japan | PROSP, CONSEC, single-center, Ncomp, ITRV, case series | Low |
| Inoue et al. 2014 | Japan | PROSP, CONSEC, Ncomp, ITRV, case series | Very low |
| Kimura et al. 2015 | Japan | PROSP, CONSEC, single-center, Ncomp, ITRV, case series | Low |
| Shin et al. 2015 | Korea | Retro, COMPR, ITRV, case series | Low |
| Hirashima et al. 2015 | Japan | Retro, case series | Very low |
| Wei et al. 2015 | China | PROSP, CONSEC, Ncomp, ITRV, case series | Low |
| Kim et al. 2015 | South Korea | Retro, case series | Very low |
| Kumar et al. 2016 | India | Retro, case series | Very low |
| Dimopoulos et al. 2015 | Germany | Retro, case series | Very low |
| Araújo et al. 2016 | Portugal | Retro, case series | Very low |
| Kim et al. 2015 | South Korea | Retro, case series | Very low |
| Shin et al. 2016 | South Korea | Retro, case series | Very low |
| Lin et al. 2016 | Taiwan | PROSP, CONSEC, single-center, COMPR, ITRV, case series | Moderate |
| de Jong et al. 2016 | Netherlands | RCT | High |
| Fassbender et al. 2016 | USA | Retro, COMPR, ITRV, case series | Low |
| Kitagawa et al. 2016 | Japan | PROSP, CONSEC, single-center, Ncomp, ITRV, case series | Low |
| González-López et al. 2016 | UK | Retro, case series | Very low |
| de Silva et al. 2016 | UK | PROSP, CONSEC, single-center, Ncomp, ITRV, case series | Low |
| Lee et al. 2016 | South Korea | Retro, case series | Very low |
| Waizel et al. 2016 | Switzerland | Retro, case series | Very low |
| Khan et al. 2017 | USA | Retro, case series | Very low |
| Bae K et al. 2016 | Korea | Retro, case series | Very Low |
| Abdelkader E et al. 2016 | UK | Retro, case series | Very Low |
| Bell et al. 2017 | USA | Retro, COMPR, ITRV, case series | Low |
| Waizel et al. 2017 | Germany | Retro, case series | Very low |
| Treumer et al. 2017 | Germany | PROSP, CONSEC, Ncomp, ITRV, case series | Low |
| Fleissig et al. 2017 | Israel | Retro, CONSEC, Ncomp, ITRV, case series | Low |
| Gok et al. 2017 | Turkey | Retro, CONSEC, Ncomp, ITRV, case series | Low |
| Kimura et al. 2017 | Japan | Retro, case series | Very low |
| Plemel et al. 2019 | Canada | Retro, CONSEC, Ncomp, ITRV, case series | Low |
| Bardak et al. 2018 | Turkey | PROSP, CONSEC, single-center, Ncomp, ITRV, case series | Low |
| Kim et al. 2018 | South Korea | Retro, case series | Very low |
| Kang et al. 2018 | Korea | Retro, CONSEC, COMPR, ITRV, case series | Low |
| Kimura et al. 2018 | Japan | Retro, CONSEC, COMPR, ITRV, case series | Low |
| Juncal et al. 2018 | Canada | Retro, CONSEC, Ncomp, ITRV, case series | Low |
| Ozkaya et al. 2018 | Turkey | Retro, case series | Very low |
| Doi et al. 2020 | Japan | PROSP, CONSEC, Ncomp, ITRV, case series | Low |
| Sharma et al. 2018 | USA | Retro, CONSEC, Ncomp, ITRV, case series | Low |
| Boiché et al. 2019 | France | Retro, CONSEC, Ncomp, ITRV, case series | Low |
| Kim et al. 2020 | South Korea | RCT | High |
| Erdogan et al. 2020 | Turkey | Retro, CONSEC, Ncomp, ITRV, case series | Low |
| Sniatecki et al. 2021 | UK | Retro, COMPR, ITRV, case series | Low |
| Gujral et al. 2019 | India | Retro, COMPR, ITRV, case series | Low |
| Maggio et al. 2020 | Italy | Retro, case series | Very low |
| Lee et al. 2020 | Korea | Retro, Ncomp, case series | Very Low |
| Cho et al. 2020 | Taiwan | Retro, case series | Very low |
| Helaiwa et al. 2020 | Germany | PROSP, CONSEC, single-center, Ncomp, ITRV, case series | Low |
| Wilkins et al. 2020 | USA | Retro, case series | Very low |
| Grohmann et al. 2020 | Germany | PROSP, CONSEC, single-center, COMPR, ITRV, case series | Moderate |
| Jeong et al. 2020 | Korea | Retro, COMPR, ITRV, case series | Low |
| Kishikova et al. 2021 | UK | PROSP, single-center, COMPR, ITRV, case series | Low |
| Karamitsos et al. 2020 | Greece | Retro, case series | Very low |
| Lim et al. 2020 | South Korea | Retro, case series | Very low |
| Lee et al. 2021 | Korea | Retro, COMPR, ITRV, case series | Low |
| Avcı et al. 2021 | Turkey | Retro, case series | Very low |
| Rickmann et al. 2021 | Germany | Retro, CONSEC, COMPR, ITRV, case series | Low |
| Tranos et al. 2021 | Greece | Retro, COMPR, ITRV, case series | Low |
| Matsuo et al. 2021 | Japan | Retro, case series | Very Low |
| Chew et al. 2022 | UK | Retro, CONSEC, Ncomp, ITRV, case series | Low |
| Iannetta et al. 2021 | Italy | Retro, case series | Very low |
| Caporossi et al. 2022 | Italy | Retro, CONSEC, COMPR, ITRV, case series | Moderate |
| Mehta et al. 2022 | UK | Secondary analyses of an RCT of image and clinical data | High |
| Kawakami et al. 2021 | Japan | Retro, case series | Very Low |
| Pierre et al. 2021 | France, Spain, Canada | Retro, CONSEC, Ncomp, ITRV, case series | Low |
| Ali Said et al. 2021 | Belgium | Retro, CONSEC, Ncomp, ITRV, case series | Low |
| Jackson et al. 2022 | Austria, Belgium, Bulgaria, Denmark, France, Germany, Hungary, Ireland, Italy, Luxemburg, Netherlands, Poland, Portugal, Slovenia, Spain, Switzerland and UK | RCT | High |
| Fukuda et al. 2022 | Japan | Retro, case series | Very low |
| Ura et al. 2022 | Japan | Retro, CONSEC, COMPR, ITRV, case series | Low |
| Kitagawa et al. 2022 | Japan | Secondary analyses of a Prospective Trial | Moderate |
| Tiosano et al. 2023 | Israel | Retro, COMPR, ITRV, case series | Low |
| Ye et al. 2023 | China | RCT | High |
| Inoue et al. 2022 | Japan | Retro, CONSEC, COMPR, ITRV, case series | Low |
| Iglicki et al. 2022 | Argentine | Retro, COMPR, ITRV, case series | Low |
| Mun et al. 2022 | Korea | Retro, COMPR, ITRV, case series | Low |
| Kimura et al. 2022 | Japan | Retro, COMPR, ITRV, case series | Low |
| Patikulsila et al. 2022 | Thailand | PROSP, CONSEC, Ncomp, ITRV, case series | Low |
| Wakabayashi et al. 2023 | Japan | Retro, case series | Very low |
| Ogata et al. 2022 | Japan | Retro, case series | Very low |
| Maruyama-Inoue et al. 2023 | Japan | PROSP, CONSEC, single-center, Ncomp, ITRV, case series | Low |
| Gabrielle et al. 2023 | France | RCT | High |
| Boral et al. 2023 | India | Retro, COMPR, ITRV, case series | Moderate |
| Wu et al. 2023 | China | Retro, COMPR, ITRV, case series | Low |
| Handa et al. 2023 | India | PROSP, CONSEC, Ncomp, ITRV, case series | Low |
| Miki et al. 2023 | Japan | PROSP, CONSEC, Ncomp, ITRV, case series | Low |
| Iglicki et al. 2024 | Argentine | Retro, CONSEC, COMPR, ITRV, case series | Low |
| Barayev et al. 2024 | Israel | Retro, COMPR, ITRV, case series | Low |
| Limon et al. 2023 | Turkey | Retro, case series | Very low |
| Szeto et al. 2024 | China | Retro, CONSEC, COMPR, ITRV, case series | Low |
| Shi et al. 2024 | China | PROSP, CONSEC, COMPR, ITRV, case series | Moderate |
| Hosokawa et al. 2024 | Japan | Retro, CONSEC, ITRV, case series | Low |
| Barzelay et al. 2024 | Israel | Retro, CONSEC, COMPR, ITRV, case series | Low |
| Wang et al. 2024 | China | Retro, case series | Very low |
| Kabakci et al. 2021 | Turkey | Retro, COMPR, ITRV, case series | Low |

**eTable 2.** Direct and indirect estimates comparing the efficacy of different treatments in BCVA for SMH. AM: amniotic membrane; AntiVEGF: Anti-vascular endothelial growth factor; ARCPGT: autologous retinal pigmentary retinal pigment epithelium-choroid patch graft transplantations; PD: pneumatic displacement; PPV: pars plana vitrectomy; PTD: Photodynamic Therapy; rtPA: recombinant tissue plasminogen activator; SRet: subretinal; IVit: intravitreal injection.

| **INDIRECT EVIDENCE** | **AntiVEGF** | . | 0.17 (-0.31; 0.65) | -0.11 (-0.47; 0.24) | -0.01 (-0.27; 0.25) | . | . | -0.37 (-0.93; 0.19) | . | . | . | . | -0.65 (-1.50; 0.20) | . | . | . | . | -0.02 (-0.42; 0.38) | . | . | -0.49 (-0.98; 0.00) | **DIRECT EVIDENCE** |
| --- | --- | --- | --- | --- | --- | --- | --- | --- | --- | --- | --- | --- | --- | --- | --- | --- | --- | --- | --- | --- | --- | --- |
|  | -0.90 (-1.66; -0.14) | **ARCPGT** | . | . | . | . | . | . | . | . | . | . | . | . | 0.70 (0.16; 1.24) | . | 0.53 (0.06; 1.00) | . | . | . | . |  |
|  | 0.11 (-0.37; 0.58) | 1.01 (0.12; 1.89) | **Observation** | 0.22 (-0.50; 0.94) | . | . | . | . | . | . | . | . | . | . | . | . | . | 0.00 (-0.70; 0.70) | . | . | . |  |
|  | -0.05 (-0.33; 0.22) | 0.85 (0.11; 1.58) | -0.16 (-0.68; 0.36) | **PD** | -0.56 (-1.26; 0.14) | . | . | . | 0.02 (-0.46; 0.51) | -0.42 (-0.88; 0.04) | . | . | . | -1.00 (-1.66; -0.34) | -0.21 (-0.98; 0.56) | 0.23 (-0.33; 0.79) | . | -0.22 (-0.89; 0.45) | . | . | -0.15 (-0.65; 0.35) |  |
|  | -0.04 (-0.27; 0.19) | 0.86 (0.09; 1.64) | -0.14 (-0.66; 0.37) | 0.02 (-0.29; 0.32) | **PD + AntiVEGF** | . | -0.18 (-1.11; 0.75) | . | -0.32 (-0.71; 0.07) | . | . | . | -0.50 (-1.48; 0.48) | . | . | . | . | -0.35 (-0.99; 0.29) | . | . | . |  |
|  | -0.74 (-1.62; 0.15) | 0.16 (-0.93; 1.26) | -0.84 (-1.84; 0.15) | -0.68 (-1.56; 0.20) | -0.70 (-1.57; 0.18) | **PD + AntiVEGF + PTD** | 0.28 (-0.26; 0.82) | . | . | . | . | . | . | . | . | . | . | . | . | . | . |  |
|  | -0.46 (-1.16; 0.25) | 0.44 (-0.51; 1.40) | -0.56 (-1.40; 0.28) | -0.40 (-1.10; 0.29) | -0.42 (-1.11; 0.27) | 0.28 (-0.26; 0.82) | **PD + AntiVEGF + rtPA** | . | . | 0.32 (-0.55; 1.20) | . | . | . | . | . | . | . | . | . | . | . |  |
|  | -0.23 (-0.72; 0.26) | 0.67 (-0.22; 1.56) | -0.34 (-1.01; 0.34) | -0.18 (-0.70; 0.35) | -0.19 (-0.72; 0.34) | 0.51 (-0.49; 1.51) | 0.23 (-0.62; 1.07) | **PD + AntiVEGF + rtPA (IVit)** | . | . | . | . | . | . | . | -0.02 (-0.81; 0.77) | . | . | 0.18 (-0.29; 0.65) | . | . |  |
|  | -0.23 (-0.60; 0.14) | 0.67 (-0.14; 1.48) | -0.34 (-0.92; 0.25) | -0.18 (-0.53; 0.18) | -0.19 (-0.52; 0.13) | 0.51 (-0.41; 1.42) | 0.23 (-0.52; 0.97) | 0.00 (-0.59; 0.59) | **PD + rtPA** | . | . | . | . | . | . | . | . | . | . | . | . |  |
|  | -0.34 (-0.82; 0.13) | 0.56 (-0.17; 1.28) | -0.45 (-1.10; 0.21) | -0.29 (-0.70; 0.13) | -0.30 (-0.79; 0.18) | 0.39 (-0.47; 1.26) | 0.11 (-0.56; 0.79) | -0.11 (-0.77; 0.54) | -0.11 (-0.64; 0.42) | **PD + rtPA (IVit)** | . | . | . | -0.30 (-0.92; 0.32) | 0.00 (-0.55; 0.55) | . | . | . | . | . | . |  |
|  | 0.16 (-1.93; 2.25) | 1.06 (-1.16; 3.28) | 0.06 (-2.08; 2.19) | 0.22 (-1.89; 2.32) | 0.20 (-1.90; 2.30) | 0.90 (-1.37; 3.16) | 0.62 (-1.58; 2.82) | 0.39 (-1.75; 2.54) | 0.39 (-1.72; 2.51) | 0.50 (-1.63; 2.64) | **PD + rtPA (SRet)** | . | . | . | . | . | . | -0.27 (-2.33; 1.78) | . | . | . |  |
|  | -0.32 (-1.16; 0.51) | 0.58 (-0.26; 1.42) | -0.43 (-1.37; 0.52) | -0.27 (-1.08; 0.54) | -0.28 (-1.13; 0.56) | 0.42 (-0.73; 1.56) | 0.14 (-0.88; 1.15) | -0.09 (-1.04; 0.86) | -0.09 (-0.96; 0.78) | 0.02 (-0.78; 0.82) | -0.48 (-2.73; 1.77) | **PPV + AM** | . | . | 0.12 (-0.52; 0.76) | . | . | . | . | . | . |  |
|  | -0.61 (-1.43; 0.20) | 0.29 (-0.82; 1.40) | -0.72 (-1.66; 0.22) | -0.56 (-1.41; 0.29) | -0.57 (-1.40; 0.25) | 0.12 (-1.07; 1.31) | -0.16 (-1.22; 0.91) | -0.38 (-1.33; 0.56) | -0.38 (-1.26; 0.49) | -0.27 (-1.21; 0.66) | -0.77 (-3.01; 1.47) | -0.29 (-1.45; 0.87) | **PPV + AntiVEGF** | . | . | . | . | . | . | . | . |  |
|  | -0.83 (-1.47; -0.18) | 0.07 (-0.81; 0.96) | -0.93 (-1.72; -0.15) | -0.77 (-1.36; -0.18) | -0.79 (-1.44; -0.13) | -0.09 (-1.10; 0.92) | -0.37 (-1.22; 0.48) | -0.60 (-1.38; 0.19) | -0.60 (-1.28; 0.09) | -0.48 (-1.06; 0.09) | -0.99 (-3.17; 1.19) | -0.51 (-1.46; 0.44) | -0.21 (-1.24; 0.82) | **PPV + rtPA (IVit)** | . | . | . | . | . | . | . |  |
|  | -0.20 (-0.74; 0.34) | 0.70 (0.16; 1.24) | -0.31 (-1.01; 0.40) | -0.15 (-0.64; 0.35) | -0.16 (-0.71; 0.39) | 0.54 (-0.41; 1.49) | 0.26 (-0.53; 1.04) | 0.03 (-0.67; 0.73) | 0.03 (-0.56; 0.62) | 0.14 (-0.33; 0.62) | -0.36 (-2.51; 1.79) | 0.12 (-0.52; 0.76) | 0.41 (-0.55; 1.38) | 0.63 (-0.07; 1.33) | **PPV + rtPA (SRet)** | . | -0.17 (-0.72; 0.38) | . | . | . | -0.46 (-1.15; 0.23) |  |
|  | 0.03 (-0.50; 0.56) | 0.93 (0.05; 1.81) | -0.07 (-0.77; 0.62) | 0.09 (-0.40; 0.58) | 0.07 (-0.48; 0.62) | 0.77 (-0.23; 1.77) | 0.49 (-0.35; 1.33) | 0.26 (-0.31; 0.84) | 0.26 (-0.33; 0.85) | 0.37 (-0.26; 1.01) | -0.13 (-2.28; 2.02) | 0.35 (-0.59; 1.29) | 0.65 (-0.32; 1.61) | 0.86 (0.09; 1.63) | 0.23 (-0.46; 0.92) | **PPV + rtPA (SRet) + AntiVEGF** | . | . | . | . | . |  |
|  | -0.37 (-1.14; 0.40) | 0.53 (0.06; 1.00) | -0.48 (-1.36; 0.41) | -0.32 (-1.05; 0.42) | -0.33 (-1.11; 0.45) | 0.37 (-0.73; 1.46) | 0.09 (-0.87; 1.04) | -0.14 (-1.03; 0.75) | -0.14 (-0.95; 0.67) | -0.03 (-0.75; 0.70) | -0.53 (-2.75; 1.69) | -0.05 (-0.89; 0.79) | 0.24 (-0.87; 1.35) | 0.46 (-0.43; 1.34) | -0.17 (-0.72; 0.38) | -0.40 (-1.28; 0.48) | **PPV + rtPA (SRet) + AntiVEGF + Gas** | . | . | . | . |  |
|  | -0.11 (-0.49; 0.27) | 0.79 (-0.05; 1.63) | -0.22 (-0.78; 0.35) | -0.06 (-0.50; 0.38) | -0.07 (-0.49; 0.34) | 0.62 (-0.33; 1.58) | 0.34 (-0.44; 1.13) | 0.12 (-0.49; 0.73) | 0.12 (-0.38; 0.62) | 0.23 (-0.36; 0.82) | -0.27 (-2.33; 1.78) | 0.21 (-0.70; 1.11) | 0.50 (-0.39; 1.39) | 0.72 (-0.01; 1.45) | 0.09 (-0.55; 0.73) | -0.14 (-0.78; 0.49) | 0.26 (-0.58; 1.10) | **PPV + rtPA (SRet) + Gas** | . | . | . |  |
|  | -0.05 (-0.73; 0.63) | 0.85 (-0.16; 1.86) | -0.16 (-0.98; 0.67) | 0.00 (-0.70; 0.71) | -0.01 (-0.72; 0.70) | 0.69 (-0.42; 1.79) | 0.41 (-0.56; 1.37) | 0.18 (-0.29; 0.65) | 0.18 (-0.58; 0.94) | 0.29 (-0.52; 1.10) | -0.21 (-2.41; 1.98) | 0.27 (-0.79; 1.33) | 0.56 (-0.49; 1.62) | 0.78 (-0.14; 1.69) | 0.15 (-0.70; 1.00) | -0.08 (-0.83; 0.66) | 0.32 (-0.69; 1.33) | 0.06 (-0.71; 0.83) | **PPV + rtPA + AntiVEGF + Gas** | -0.13 (-0.69; 0.43) | . |  |
|  | -0.18 (-1.06; 0.70) | 0.72 (-0.44; 1.87) | -0.29 (-1.28; 0.71) | -0.13 (-1.03; 0.78) | -0.14 (-1.05; 0.77) | 0.56 (-0.69; 1.80) | 0.28 (-0.84; 1.40) | 0.05 (-0.69; 0.79) | 0.05 (-0.89; 0.99) | 0.16 (-0.82; 1.15) | -0.34 (-2.61; 1.93) | 0.14 (-1.06; 1.34) | 0.43 (-0.77; 1.63) | 0.65 (-0.43; 1.72) | 0.02 (-1.00; 1.04) | -0.21 (-1.15; 0.72) | 0.19 (-0.97; 1.35) | -0.07 (-1.03; 0.89) | -0.13 (-0.69; 0.43) | **PPV + rtPA + Gas** | . |  |
|  | -0.44 (-0.87; -0.01) | 0.46 (-0.29; 1.21) | -0.55 (-1.17; 0.08) | -0.39 (-0.80; 0.03) | -0.40 (-0.86; 0.06) | 0.29 (-0.65; 1.24) | 0.01 (-0.76; 0.79) | -0.21 (-0.84; 0.42) | -0.21 (-0.73; 0.31) | -0.10 (-0.63; 0.43) | -0.60 (-2.73; 1.53) | -0.12 (-0.94; 0.70) | 0.17 (-0.74; 1.09) | 0.38 (-0.31; 1.08) | -0.24 (-0.75; 0.27) | -0.47 (-1.10; 0.16) | -0.07 (-0.82; 0.68) | -0.33 (-0.89; 0.23) | -0.39 (-1.18; 0.40) | -0.26 (-1.23; 0.71) | **Vitrectomy** |  |

**eTable 3.** Direct and indirect estimates comparing the efficacy of different treatments in SMH resolution. AntiVEGF: Anti-vascular endothelial growth factor; ARCPGT: autologous retinal pigmentary retinal pigment epithelium-choroid patch graft transplantations; PD: pneumatic displacement; PPV: pars plana vitrectomy; rtPA: recombinant tissue plasminogen activator; SRet: subretinal; IVit: intravitreal injection.

| **INDIRECT EVIDENCE** | **AntiVEGF** | . | 0.47 (0.20;1.13) | 1.15 (0.47;2.79) | . | . | . | . | . | . | 0.76 (0.32;1.83) | 0.42 (0.17;1.00) | **DIRECT EVIDENCE** |
| --- | --- | --- | --- | --- | --- | --- | --- | --- | --- | --- | --- | --- | --- |
|  | 0.70 (0.24; 2.08) | **ARCPGT** | . | . | . | . | . | 0.79 (0.32;1.96) | . | 0.82 (0.33;2.01) | . | . |  |
|  | 0.74 (0.39; 1.40) | 1.05 (0.39; 2.81) | **PD** | 0.70 (0.28;1.75) | . | 0.98 (0.62;1.55) | . | 0.62 (0.25;1.59) | 0.44 (0.18;1.06) | . | 1.20 (0.39;3.67) | 0.88 (0.37;2.12) |  |
|  | 0.73 (0.36; 1.46) | 1.04 (0.35; 3.08) | 0.99 (0.52; 1.88) | **PD + AntiVEGF** | 1.16 (0.47;2.89) | . | . | . | . | . | 0.67 (0.28;1.61) | . |  |
|  | 0.77 (0.32; 1.86) | 1.10 (0.34; 3.59) | 1.05 (0.45; 2.42) | 1.06 (0.52; 2.16) | **PD + rtPA** | . | 0.98 (0.41;2.32) | . | . | . | 0.81 (0.34;1.97) | . |  |
|  | 0.73 (0.36; 1.50) | 1.04 (0.39; 2.77) | 0.99 (0.65; 1.52) | 1.00 (0.49; 2.07) | 0.95 (0.39; 2.30) | **PD + rtPA (IVit)** | . | 0.89 (0.47;1.68) | . | . | 0.38 (0.13;1.07) | . |  |
|  | 0.75 (0.22; 2.59) | 1.07 (0.25; 4.64) | 1.02 (0.31; 3.41) | 1.03 (0.34; 3.17) | 0.98 (0.41; 2.32) | 1.03 (0.30; 3.55) | **PPV + rtPA (IVit) + Gas** | . | . | . | . | . |  |
|  | 0.57 (0.27; 1.20) | 0.81 (0.34; 1.92) | 0.77 (0.44; 1.35) | 0.78 (0.36; 1.69) | 0.74 (0.29; 1.84) | 0.78 (0.45; 1.33) | 0.75 (0.21; 2.67) | **PPV + rtPA (SRet)** | . | 1.03 (0.43;2.45) | . | 1.00 (0.42;2.36) |  |
|  | 0.33 (0.11; 0.97) | 0.46 (0.12; 1.73) | 0.44 (0.18; 1.06) | 0.45 (0.15; 1.33) | 0.42 (0.13; 1.42) | 0.45 (0.17; 1.18) | 0.43 (0.10; 1.92) | 0.57 (0.20; 1.63) | **PPV + rtPA (SRet) + AntiVEGF** | . | . | . |  |
|  | 0.57 (0.23; 1.37) | 0.80 (0.34; 1.91) | 0.77 (0.35; 1.69) | 0.78 (0.32; 1.87) | 0.73 (0.28; 1.95) | 0.77 (0.35; 1.71) | 0.75 (0.20; 2.77) | 1.00 (0.49; 2.02) | 1.73 (0.53; 5.63) | **PPV + rtPA (SRet) + AntiVEGF + Gas** | 1.06 (0.42;2.65) | . |  |
|  | 0.58 (0.29; 1.13) | 0.82 (0.30; 2.23) | 0.78 (0.42; 1.45) | 0.79 (0.42; 1.47) | 0.74 (0.37; 1.51) | 0.79 (0.41; 1.52) | 0.76 (0.25; 2.33) | 1.01 (0.50; 2.03) | 1.76 (0.60; 5.16) | 1.02 (0.49; 2.11) | **PPV + rtPA (SRet) + Gas** | . |  |
|  | 0.54 (0.26; 1.13) | 0.77 (0.27; 2.21) | 0.73 (0.38; 1.42) | 0.74 (0.32; 1.71) | 0.70 (0.26; 1.88) | 0.74 (0.36; 1.51) | 0.72 (0.19; 2.66) | 0.95 (0.49; 1.83) | 1.66 (0.55; 4.96) | 0.96 (0.39; 2.34) | 0.94 (0.42; 2.08) | **Vitrectomy** |  |

**eTable 4.** Direct and indirect estimates comparing the security of different treatments in terms of retinal detachment. AntiVEGF: Anti-vascular endothelial growth factor; ARCPGT: autologous retinal pigmentary retinal pigment epithelium-choroid patch graft transplantations; PD: pneumatic displacement; PPV: pars plana vitrectomy; rtPA: recombinant tissue plasminogen activator; SRet: subretinal; IVit: intravitreal injection.

| **INDIRECT EVIDENCE** | **AntiVEGF** | . | 0.18 (0.02;1.68) | 0.56 (0.12;2.66) | 0.41 (0.09;1.94) | 1.08 (0.12;9.81) | . | . | . | . | . | . | 0.39 (0.04;3.55) | . | . | . | 0.55 (0.12;2.62) | . | . | 0.29 (0.03;2.61) | **DIRECT EVIDENCE** |
| --- | --- | --- | --- | --- | --- | --- | --- | --- | --- | --- | --- | --- | --- | --- | --- | --- | --- | --- | --- | --- | --- |
|  | 0.03 (0.00;0.39) | **ARCPGT** | . | . | . | . | . | . | . | . | . | . | . | 8.73 (0.95;80.03) | . | 3.44 (0.37;31.52) | . | . | . | . |  |
|  | 0.16 (0.02;1.09) | 4.93 (0.25; 96.99) | **Observation** | 1.45 (0.16;13.40) | . | . | . | . | . | . | . | . | . | . | . | . | 6.18 (0.67;56.79) | . | . | . |  |
|  | 0.31 (0.09;1.06) | 9.83 (0.84;115.18) | 1.99 (0.28; 14.27) | **PD** | 6.27 (0.68;57.55) | . | . | 1.42 (0.30;6.76) | 1.08 (0.12;9.92) | . | . | . | . | 1.88 (0.20;17.17) | 0.59 (0.06;5.34) | . | 4.25 (0.46;38.89) | . | . | 0.25 (0.03;2.24) |  |
|  | 0.82 (0.22;3.08) | 26.05 (1.79;378.39) | 5.28 (0.59; 47.21) | 2.65 (0.62; 11.27) | **PD + AntiVEGF** | . | . | . | . | . | . | . | . | . | . | . | 0.31 (0.03;2.80) | . | . | . |  |
|  | 0.50 (0.09;2.89) | 15.91 (0.98;259.07) | 3.23 (0.26; 40.18) | 1.62 (0.24; 10.93) | 0.61 (0.07;5.00) | **PD + AntiVEGF + rtPA** | . | . | 2.08 (0.23;18.92) | . | 0.38 (0.04;3.57) | . | . | . | . | . | . | . | . | . |  |
|  | 1.39 (0.12; 16.31) | 44.14 (1.73;1123.34) | 8.95 (0.50;160.09) | 4.49 (0.44; 45.83) | 1.69 (0.13; 22.41) | 2.77 (0.15; 49.99) | **PD + AntiVEGF + rtPA (IVit)** | . | . | . | . | . | . | . | 0.11 (0.01;1.01) | . | . | 1.30 (0.14;11.86) | . | . |  |
|  | 0.44 (0.06;3.20) | 13.93 (0.75;257.03) | 2.82 (0.23; 34.86) | 1.42 (0.30;6.76) | 0.53 (0.06;4.50) | 0.88 (0.07; 10.34) | 0.32 (0.02;5.19) | **PD + rtPA** | . | . | . | . | . | . | . | . | . | . | . | . |  |
|  | 0.48 (0.10;2.36) | 15.34 (1.44;162.99) | 3.11 (0.31; 31.10) | 1.56 (0.36;6.82) | 0.59 (0.09;3.80) | 0.96 (0.17;5.56) | 0.35 (0.02;4.93) | 1.10 (0.13;9.45) | **PD + rtPA (IVit)** | . | . | . | . | 0.58 (0.16;2.06) | . | . | . | . | . | 1.61 (0.17;14.88) |  |
|  | 0.37 (0.03;5.04) | 11.89 (0.43;329.30) | 2.41 (0.12; 47.60) | 1.21 (0.09; 16.84) | 0.46 (0.03;6.90) | 0.75 (0.04; 15.53) | 0.27 (0.01;6.70) | 0.85 (0.04; 18.25) | 0.78 (0.05; 13.20) | **PD + rtPA (SRet)** | . | . | . | . | . | . | 1.58 (0.17;14.54) | . | . | . |  |
|  | 0.19 (0.01;3.26) | 5.96 (0.17;215.28) | 1.21 (0.04; 35.59) | 0.61 (0.03; 11.63) | 0.23 (0.01;4.99) | 0.37 (0.04;3.57) | 0.14 (0.00;5.28) | 0.43 (0.02; 12.11) | 0.39 (0.02; 6.75) | 0.50 (0.01; 21.93) | **PD + rtPA + PTD** | . | . | . | . | . | . | . | . | . |  |
|  | 0.28 (0.02;4.66) | 8.99 (0.42;193.04) | 1.82 (0.07; 46.34) | 0.91 (0.06; 13.80) | 0.35 (0.02;6.58) | 0.57 (0.03; 11.20) | 0.20 (0.01;6.63) | 0.65 (0.03; 14.79) | 0.59 (0.05;7.31) | 0.76 (0.02; 27.92) | 1.51 (0.04; 63.48) | **PPV + AM** | . | 1.00 (0.11;9.15) | . | . | . | . | . | . |  |
|  | 0.39 (0.04;3.55) | 12.36 (0.43;353.68) | 2.51 (0.13; 47.76) | 1.26 (0.10; 15.74) | 0.47 (0.04;6.23) | 0.78 (0.05; 13.03) | 0.28 (0.01;7.65) | 0.89 (0.05; 17.32) | 0.81 (0.05; 12.23) | 1.04 (0.03; 31.46) | 2.07 (0.06; 76.50) | 1.37 (0.04; 48.64) | **PPV + rtPA (IVit) + Gas + AntiVEGF** | . | . | . | . | . | . | . |  |
|  | 0.28 (0.05;1.57) | 8.99 (1.08; 75.08) | 1.82 (0.17; 19.30) | 0.91 (0.19;4.39) | 0.35 (0.05;2.42) | 0.57 (0.08;4.19) | 0.20 (0.01;3.00) | 0.65 (0.07;5.92) | 0.59 (0.17;1.97) | 0.76 (0.04; 13.07) | 1.51 (0.07; 30.73) | 1.00 (0.11;9.15) | 0.73 (0.04; 11.92) | **PPV + rtPA (SRet)** | . | 0.39 (0.04;3.58) | . | . | . | . |  |
|  | 0.17 (0.02;1.52) | 5.31 (0.25;113.24) | 1.08 (0.07; 15.73) | 0.54 (0.08;3.79) | 0.20 (0.02;2.12) | 0.33 (0.02;4.80) | 0.12 (0.02;0.84) | 0.38 (0.03;4.63) | 0.35 (0.03;3.77) | 0.45 (0.02; 10.09) | 0.89 (0.03; 29.17) | 0.59 (0.02; 15.89) | 0.43 (0.02;9.77) | 0.59 (0.05;6.76) | **PPV + rtPA (SRet) + AntiVEGF** | . | . | . | . | . |  |
|  | 0.11 (0.01;0.78) | 3.33 (0.40; 27.84) | 0.68 (0.05;8.52) | 0.34 (0.05;2.43) | 0.13 (0.01;1.14) | 0.21 (0.02;2.33) | 0.08 (0.00;1.27) | 0.24 (0.02;2.96) | 0.22 (0.03;1.58) | 0.28 (0.02;4.89) | 0.56 (0.02; 15.10) | 0.37 (0.02;6.43) | 0.27 (0.01;5.34) | 0.37 (0.06;2.24) | 0.63 (0.04;8.88) | **PPV + rtPA (SRet) + AntiVEGF + Gas** | 6.00 (0.65;55.00) | . | . | . |  |
|  | 0.59 (0.15;2.30) | 18.83 (1.59;223.12) | 3.82 (0.52; 28.06) | 1.92 (0.46;7.92) | 0.72 (0.15;3.46) | 1.18 (0.15;9.38) | 0.43 (0.04;4.36) | 1.35 (0.16; 11.17) | 1.23 (0.21;7.18) | 1.58 (0.17; 14.54) | 3.16 (0.15; 67.26) | 2.09 (0.12; 36.09) | 1.52 (0.11; 20.32) | 2.09 (0.35; 12.54) | 3.55 (0.40; 31.73) | 5.65 (0.93; 34.28) | **PPV + rtPA (SRet) + Gas** | 2.60 (0.28;24.04) | . | . |  |
|  | 1.67 (0.18; 15.89) | 53.10 (2.47;1141.94) | 10.77 (0.73;159.57) | 5.40 (0.60; 48.57) | 2.04 (0.19; 22.10) | 3.34 (0.22; 51.07) | 1.20 (0.17;8.42) | 3.81 (0.26; 56.48) | 3.46 (0.29; 41.72) | 4.46 (0.23; 85.89) | 8.90 (0.26;306.08) | 5.90 (0.21;168.59) | 4.30 (0.18;100.63) | 5.90 (0.48; 73.15) | 10.00 (0.98;102.02) | 15.92 (1.19;213.12) | 2.82 (0.40; 19.94) | **PPV + rtPA (SRet) + Gas + AntiVEGF** | . | . |  |
|  | 0.51 (0.03;8.37) | 16.29 (0.49;538.02) | 3.30 (0.12; 87.62) | 1.66 (0.10; 26.30) | 0.63 (0.03; 12.28) | 1.02 (0.05; 22.41) | 0.37 (0.01; 12.79) | 1.17 (0.05; 28.01) | 1.06 (0.07; 16.83) | 1.37 (0.03; 54.98) | 2.73 (0.06;124.60) | 1.81 (0.05; 70.08) | 1.32 (0.04; 46.31) | 1.81 (0.10; 33.23) | 3.07 (0.11; 87.14) | 4.88 (0.20;119.73) | 0.86 (0.05; 16.57) | 0.31 (0.01;9.47) | **PPV + rtPA + Gas** | 0.58 (0.06;5.31) |  |
|  | 0.30 (0.05;1.65) | 9.50 (0.63;142.96) | 1.93 (0.17; 21.70) | 0.97 (0.18;5.09) | 0.36 (0.05;2.69) | 0.60 (0.07;5.15) | 0.22 (0.01;3.45) | 0.68 (0.07;6.68) | 0.62 (0.12;3.26) | 0.80 (0.04; 15.39) | 1.59 (0.07; 35.96) | 1.06 (0.06; 19.44) | 0.77 (0.05; 12.52) | 1.06 (0.16;7.01) | 1.79 (0.14; 22.10) | 2.85 (0.28; 28.81) | 0.50 (0.07;3.58) | 0.18 (0.01;2.47) | 0.58 (0.06;5.31) | **Vitrectomy** |  |

**eTable 5.** Direct and indirect estimates comparing the security of different treatments in terms of vitreous hemorraghe. AntiVEGF: Anti-vascular endothelial growth factor; ARCPGT: autologous retinal pigmentary retinal pigment epithelium-choroid patch graft transplantations; PD: pneumatic displacement; PPV: pars plana vitrectomy; rtPA: recombinant tissue plasminogen activator; SRet: subretinal; IVit: intravitreal injection.

| **INDIRECT EVIDENCE** | **AntiVEGF** | . | 0.11 (0.03;0.35) | 0.69 (0.29;1.60) | . | 1.08 (0.32;3.60) | . | . | . | . | . | . | . | 0.28 (0.08;0.92) | 0.12 (0.04;0.40) | **DIRECT EVIDENCE** |
| --- | --- | --- | --- | --- | --- | --- | --- | --- | --- | --- | --- | --- | --- | --- | --- | --- |
|  | 0.39 (0.10; 1.53) | **ARCPGT** | . | . | . | . | . | . | . | . | 2.91 (0.88;9.60) | . | . | 0.64 (0.19;2.10) | . |  |
|  | 0.15 (0.07; 0.32) | 0.39 (0.10; 1.54) | **PD** | 4.88 (1.44; 16.57) | . | . | 7.94 (2.35; 26.89) | 3.25 (0.96; 11.02) | . | 0.73 (0.22;2.44) | 5.62 (1.66; 19.07) | 0.59 (0.18;1.96) | . | . | 1.16 (0.35;3.84) |  |
|  | 0.90 (0.44; 1.85) | 2.33 (0.54;10.14) | 5.95 (2.73;12.97) | **PD + AntiVEGF** | . | . | 0.29 (0.09;0.98) | . | . | . | . | . | . | . | . |  |
|  | 0.21 (0.04; 1.12) | 0.55 (0.09; 3.24) | 1.41 (0.29; 6.76) | 0.24 (0.04; 1.30) | **PD + AntiVEGF + rtPA** | . | . | 2.08 (0.63;6.85) | . | . | . | . | . | . | . |  |
|  | 0.47 (0.17; 1.29) | 1.21 (0.23; 6.30) | 3.09 (1.02; 9.30) | 0.52 (0.16; 1.68) | 2.19 (0.34;14.19) | **PD + AntiVEGF + rtPA (IVit)** | . | . | . | . | . | 1.00 (0.30;3.30) | . | . | . |  |
|  | 0.56 (0.19; 1.62) | 1.45 (0.29; 7.29) | 3.70 (1.44; 9.53) | 0.62 (0.24; 1.60) | 2.63 (0.43;16.04) | 1.20 (0.30; 4.74) | **PD + rtPA** | . | . | . | . | . | . | . | . |  |
|  | 0.44 (0.14; 1.40) | 1.15 (0.31; 4.24) | 2.92 (1.06; 8.09) | 0.49 (0.15; 1.66) | 2.08 (0.63; 6.85) | 0.95 (0.23; 3.99) | 0.79 (0.20; 3.08) | **PD + rtPA (IVit)** | . | . | 2.40 (1.21;4.79) | . | . | . | . |  |
|  | 5.40 (1.09;26.82) | 13.96 (2.66;73.33) | 35.64 (7.69; 165.17) | 5.99 (1.14;31.53) | 25.31 (4.10; 156.35) | 11.55 (1.86;71.77) | 9.63 (1.63;56.71) | 12.18 (3.08;48.24) | **PPV + AM + SO** | . | 0.20 (0.06;0.66) | . | . | . | . |  |
|  | 0.11 (0.03; 0.46) | 0.29 (0.05; 1.77) | 0.73 (0.22; 2.44) | 0.12 (0.03; 0.52) | 0.52 (0.07; 3.75) | 0.24 (0.05; 1.21) | 0.20 (0.04; 0.91) | 0.25 (0.05; 1.21) | 0.02 (0.00; 0.14) | **PPV + rtPA (IVit)** | . | . | . | . | . |  |
|  | 1.08 (0.37; 3.14) | 2.79 (0.88; 8.82) | 7.13 (2.73;18.62) | 1.20 (0.38; 3.79) | 5.06 (1.28;19.99) | 2.31 (0.58; 9.19) | 1.93 (0.52; 7.14) | 2.44 (1.23; 4.82) | 0.20 (0.06; 0.66) | 9.78 (2.09;45.85) | **PPV + rtPA (SRet)** | . | . | 0.22 (0.07;0.72) | . |  |
|  | 0.21 (0.07; 0.62) | 0.53 (0.10; 2.76) | 1.36 (0.49; 3.74) | 0.23 (0.07; 0.75) | 0.96 (0.15; 6.08) | 0.44 (0.16; 1.21) | 0.37 (0.10; 1.41) | 0.46 (0.11; 1.89) | 0.04 (0.01; 0.23) | 1.86 (0.38; 9.03) | 0.19 (0.05; 0.74) | **PPV + rtPA (SRet) + AntiVEGF** | . | . | . |  |
|  | 0.49 (0.10; 2.41) | 1.27 (0.24; 6.89) | 3.25 (0.63;16.80) | 0.55 (0.10; 2.98) | 2.31 (0.29;18.22) | 1.05 (0.17; 6.70) | 0.88 (0.14; 5.52) | 1.11 (0.21; 6.00) | 0.09 (0.01; 0.67) | 4.47 (0.58;34.31) | 0.46 (0.09; 2.23) | 2.40 (0.37;15.49) | **PPV + rtPA (SRet) + Gas** | 0.52 (0.15;1.79) | . |  |
|  | 0.26 (0.09; 0.70) | 0.66 (0.21; 2.09) | 1.69 (0.57; 4.99) | 0.28 (0.09; 0.91) | 1.20 (0.23; 6.29) | 0.55 (0.14; 2.17) | 0.46 (0.12; 1.78) | 0.58 (0.18; 1.82) | 0.05 (0.01; 0.23) | 2.32 (0.46;11.77) | 0.24 (0.09; 0.64) | 1.25 (0.31; 5.05) | 0.52 (0.15; 1.79) | **PPV + rtPA (SRet) + Gas + AntiVEGF** | . |  |
|  | 0.15 (0.05; 0.44) | 0.38 (0.07; 2.03) | 0.97 (0.32; 2.92) | 0.16 (0.05; 0.55) | 0.69 (0.11; 4.51) | 0.31 (0.08; 1.32) | 0.26 (0.07; 1.06) | 0.33 (0.08; 1.42) | 0.03 (0.00; 0.17) | 1.33 (0.26; 6.84) | 0.14 (0.03; 0.55) | 0.72 (0.17; 3.00) | 0.30 (0.05; 1.95) | 0.57 (0.14; 2.37) | **Vitrectomy** |  |

**eTable 6.** Direct and indirect estimates comparing the security of different treatments in terms of recurrent SMH. AntiVEGF: Anti-vascular endothelial growth factor; ARCPGT: autologous retinal pigmentary retinal pigment epithelium-choroid patch graft transplantations; PD: pneumatic displacement; PPV: pars plana vitrectomy; rtPA: recombinant tissue plasminogen activator; SRet: subretinal; IVit: intravitreal injection.

| **INDIRECT EVIDENCE** | **AntiVEGF** | . | 1.16 (0.22;6.16) | 1.02 (0.31;3.33) | 1.35 (0.41;4.39) | . | . | . | . | . | . | . | 0.86 (0.16;4.56) | **DIRECT EVIDENCE** |
| --- | --- | --- | --- | --- | --- | --- | --- | --- | --- | --- | --- | --- | --- | --- |
|  | 0.17 (0.01; 4.75) | **ARCPGT** | . | . | . | . | . | . | 0.97 (0.18;5.22) | . | 2.45 (0.46;13.17) | . | . |  |
|  | 1.16 (0.22; 6.16) | 6.97 (0.24;198.14) | **PD** | . | . | . | 1.43 (0.44;4.69) | . | . | 0.33 (0.06;1.74) | . | . | 0.74 (0.14;3.92) |  |
|  | 0.84 (0.27; 2.64) | 5.03 (0.15;172.99) | 0.72 (0.10; 5.45) | **PD + AntiVEGF** | 2.60 (0.49;13.91) | . | . | . | . | . | . | . | . |  |
|  | 1.39 (0.44; 4.36) | 8.34 (0.24;286.57) | 1.20 (0.16; 9.03) | 1.66 (0.42; 6.51) | **PD + AntiVEGF + rtPA** | . | . | . | . | . | . | . | . |  |
|  | 0.15 (0.01; 2.63) | 0.87 (0.01;52.58) | 0.13 (0.01; 1.33) | 0.17 (0.01; 3.90) | 0.10 (0.00; 2.35) | **PD + AntiVEGF + rtPA (IVit)** | . | . | . | 2.60 (0.49;13.83) | . | . | . |  |
|  | 1.66 (0.21;12.89) | 9.97 (0.29;347.83) | 1.43 (0.44; 4.69) | 1.98 (0.19;20.69) | 1.20 (0.11;12.46) | 11.44 (0.81;161.61) | **PD + rtPA** | . | . | . | . | . | . |  |
|  | 0.09 (0.00; 2.98) | 0.51 (0.07; 4.02) | 0.07 (0.00; 2.56) | 0.10 (0.00; 4.25) | 0.06 (0.00; 2.56) | 0.59 (0.01;41.96) | 0.05 (0.00; 2.17) | **PD + rtPA (IVit)** | 1.89 (0.58;6.16) | . | . | . | . |  |
|  | 0.16 (0.01; 4.59) | 0.97 (0.18; 5.22) | 0.14 (0.00; 3.94) | 0.19 (0.01; 6.61) | 0.12 (0.00; 3.98) | 1.11 (0.02;66.95) | 0.10 (0.00; 3.38) | 1.89 (0.58; 6.16) | **PPV + rtPA (SRet)** | . | 2.53 (0.48;13.48) | . | . |  |
|  | 0.38 (0.04; 4.02) | 2.27 (0.05;95.74) | 0.33 (0.06; 1.74) | 0.45 (0.03; 6.23) | 0.27 (0.02; 3.75) | 2.60 (0.49;13.83) | 0.23 (0.03; 1.77) | 4.41 (0.09;222.78) | 2.34 (0.06;98.38) | **PPV + rtPA (SRet) + AntiVEGF** | . | . | . |  |
|  | 0.41 (0.02; 7.42) | 2.45 (0.46;13.17) | 0.35 (0.02; 6.38) | 0.49 (0.02;10.99) | 0.29 (0.01; 6.62) | 2.82 (0.07;118.60) | 0.25 (0.01; 5.63) | 4.77 (0.62;37.04) | 2.53 (0.48;13.48) | 1.08 (0.04;30.76) | **PPV + rtPA (SRet) + AntiVEGF + Gas** | 0.40 (0.07;2.13) | . |  |
|  | 0.16 (0.02; 1.74) | 0.98 (0.09;10.52) | 0.14 (0.01; 1.50) | 0.20 (0.01; 2.70) | 0.12 (0.01; 1.62) | 1.13 (0.04;31.94) | 0.10 (0.01; 1.39) | 1.91 (0.14;26.92) | 1.01 (0.09;10.79) | 0.43 (0.02; 7.85) | 0.40 (0.07; 2.13) | **PPV + rtPA (SRet) + Gas** | 5.25 (0.98;27.99) |  |
|  | 0.86 (0.16; 4.56) | 5.15 (0.28;93.97) | 0.74 (0.14; 3.92) | 1.03 (0.14; 7.75) | 0.62 (0.08; 4.67) | 5.92 (0.33;107.02) | 0.52 (0.07; 4.01) | 10.03 (0.44;229.52) | 5.32 (0.29;96.46) | 2.28 (0.21;24.20) | 2.10 (0.20;22.41) | 5.25 (0.98;27.99) | **Vitrectomy** |  |
